# Western diet induces iron-dependent enteric neurodegeneration via ferroptosis

**DOI:** 10.1172/JCI196113

**Published:** 2026-04-21

**Authors:** Arun Balasubramaniam, Dmitrii Pavlov, Yunpeng Du, Jeremy Reeves, Alan Harzman, Yunshan Liu, Francesca Cingolani, Xinxu Yuan, Jay M. Patel, Simon Musyoka Mwangi, Peijian He, C. Michael Hart, Wenhui Hu, Fievos L. Christofi, Shanthi Srinivasan

**Affiliations:** 1 Division of Digestive Diseases, Emory University School of Medicine, Atlanta, Georgia, USA.; 2 Atlanta VA Health Care System, Atlanta, Georgia, USA.; 3 Department of Anesthesiology, and; 4 Department of Surgery, The Ohio State University, Columbus, Ohio, USA.; 5 Department of Neuroscience and Anatomy, Virginia Commonwealth University, Richmond, Virginia, USA.; 6 Department of Orthopaedics, and; 7 Division of Pulmonary, Allergy, Critical Care and Sleep Medicine, Emory University School of Medicine, Atlanta, Georgia, USA.

**Keywords:** Gastroenterology, Neuroscience, Neurodegeneration, Obesity

## Abstract

The Western diets (WD), high in saturated fats such as palmitic acid (PA), promotes enteric neurodegeneration and motility disorders. Using murine models, in vitro systems, and human myenteric ganglia, we investigated whether a WD and PA drive iron-dependent ferroptotic injury in the enteric nervous system (ENS). Mice were fed a control diet (CD) or a WD for 12 weeks, with or without systemic AAV9-MaCPNS2 delivery of Nfe2l2 to enteric neurons. Colonic motility was assessed by a bead expulsion assay. We assessed ferroptosis using convergent readouts including iron dysregulation (transferrin receptor 1 [TfR1], ferritin heavy chain 1 [FTH1], labile and mitochondrial iron [Fe^2+^]), lipid peroxidation (C11-BODIPY and 4-hydroxynonenal [4-HNE]), glutathione peroxidase 4 (GPX4) suppression, and pharmacologic inhibition by ferrostatin 1 (Fer-1) in primary enteric neurons, murine myenteric plexuses, and human networks of myenteric ganglia (nhMPG). WD-fed mice exhibited delayed colonic transit, increased TfR1 and FTH1, and vulnerability of nNOS neurons; these changes were reversed by nuclear factor erythroid 2–related factor 2; (Nfe2l2, also known as Nrf2) overexpression. RNA-seq of PA-treated immortalized murine fetal enteric neurons (IM-FENs) revealed disrupted neurotransmitter signaling, reduced mitochondrial and antioxidant programs, and increased iron import and lipid peroxidation signatures. PA increased labile Fe^2+^, mitochondrial ROS, membrane depolarization, Ca^2+^ dysregulation, 4-HNE, and mitoferrin 2 (Mfrn2), whereas Fer-1 preserved mitochondrial integrity, viability, and ENS function. In human nhMPG, PA induced enteric neuronal iron loading and ferroptosis, supporting the translational relevance to diet-associated enteric neuropathy.

## Abstract 2

Western diet triggers iron-driven neuronal death in the gut. Targeting ferroptosis restores enteric function, revealing a potential therapy for diet-induced motility disorders.

## Graphical abstract

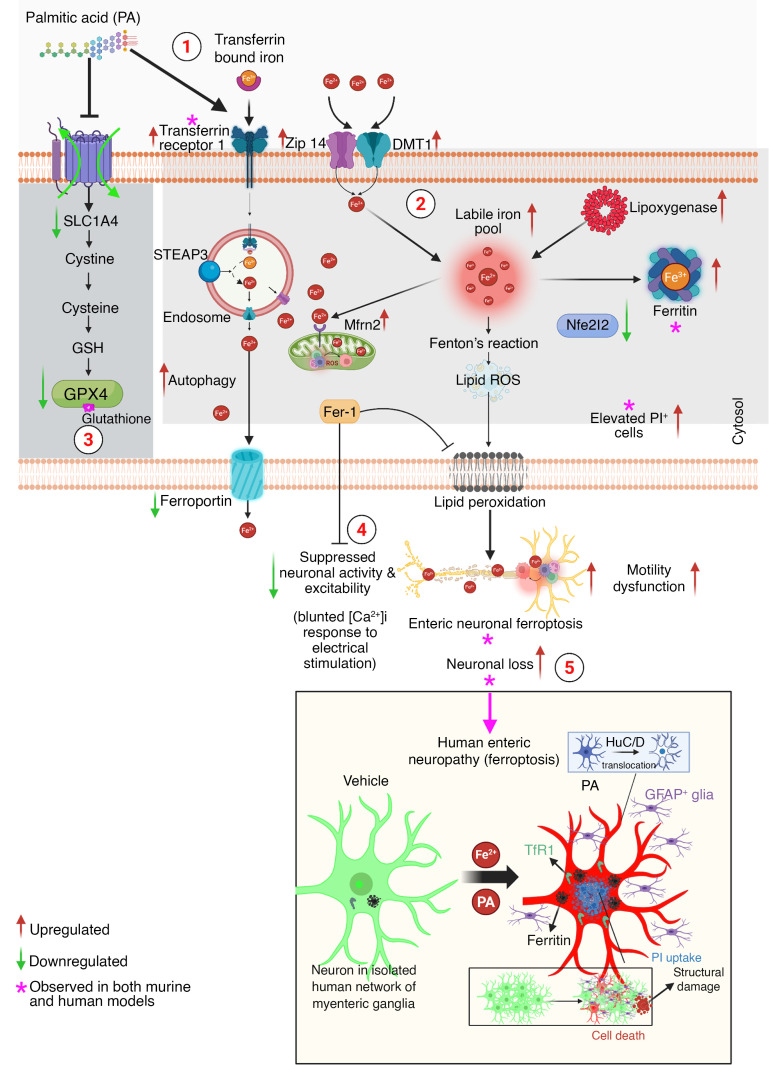

## Introduction

The enteric nervous system (ENS), a complex and autonomous network of neurons and glia embedded within the gastrointestinal (GI) tract, is essential for regulating gut motility, secretion, immune responses, and barrier function (1). Often referred to as the “second brain” (2), the ENS operates independently of the central nervous system (CNS), yet remains tightly integrated with systemic physiological cues (1, 3). Notably, its neuronal populations are largely postmitotic, and exhibit limited regenerative capacity, rendering them particularly vulnerable to chronic metabolic insults (4, 5). In parallel with the global rise in obesity and metabolic syndrome, GI symptoms such as constipation, altered motility, bloating, and discomfort are increasingly prevalent in affected individuals (6). Despite the high burden of GI dysfunction in these populations, the mechanisms linking diet-induced metabolic stress to enteric neuronal injury are not well understood. Studies suggest that disruptions in neuronal homeostasis within the myenteric plexus may underlie these symptoms (7), but the molecular underpinnings remain largely unexplored. Among dietary components, saturated fatty acids (SFAs), particularly palmitic acid (PA), are consistently associated with adverse metabolic and inflammatory outcomes (8). PA is not only a key contributor to lipotoxicity in peripheral tissues, such as liver and adipose tissue, but also induces oxidative stress and cell death in central and peripheral neurons (9, 10). Here we studied the role of iron overload in Western diet/PA–induced (WD/PA-induced) enteric neurodegeneration with a focus on ferroptosis. WD/PA disrupts ENS function, but the regulated death mechanism is unclear. We focused on ferroptosis,since it is an iron-driven lipid peroxidation program, and on nuclear factor erythroid 2–related factor 2 (Nfe2l2, also known as Nrf2) because it controls antioxidant and lipid-peroxide detoxification pathways that restrain ferroptotic susceptibility.

A growing body of research implicates ferroptosis, a form of regulated cell death distinct from apoptosis and necrosis, as a key mechanism of metabolic and neurodegenerative diseases (11). Ferroptosis is characterized by iron accumulation, lipid peroxidation, depletion of glutathione (GSH), and inactivation of glutathione peroxidase 4 (GPX4) (12). Mitochondria dysfunction (13), redox imbalance, and failure to resolve ROS contribute to this cascade, ultimately leading to loss of membrane integrity and cell death (11). Although ferroptosis has been documented in the CNS (14), its contribution to enteric neurodegeneration under high-fat dietary conditions has not, to our knowledge, been investigated. Moreover, enteric neurons are not the only ENS-resident cells susceptible to lipid stress. Enteric glial cells, which play critical roles in neuroprotection, neurotransmission, and inflammation, may also respond to oxidative injury and contribute to disease progression (15). Nfe2l2 serves as a central regulator of antioxidant defenses and cytoprotective gene expression. By activating antioxidant response elements (AREs), Nfe2l2 orchestrates cellular defense programs against oxidative and electrophilic stress (16). Prior studies have shown that loss of Nfe2l2 sensitizes neurons to ferroptosis (17), whereas pharmacologic or genetic activation of this pathway confers protection in various cell types (18, 19). However, whether Nfe2l2 plays a similarly protective role in the ENS, particularly under conditions of chronic lipid and saturated fatty acid (SFA) overload, has not, to our knowledge, been determined.

In this study, we tested the hypothesis that WD and SFAs such as PA cause intestinal ENS neuropathy and disruption of motility by induction of ferroptosis in mice and humans and identified the underlying cellular and molecular mechanisms. We also explored whether Nfe2l2, a central regulator with antioxidant properties, could protect against damage of the ENS under conditions of chronic lipid overload. A comprehensive investigation characterized the effects of PA on enteric neurons using immortalized murine fetal enteric neurons (IM-FENs), primary enteric neuronal cultures, in vivo dietary models, and freshly isolated intact human neural networks of myenteric ganglia (nhMPG) from patients who had undergone colectomy. In designing these experiments, we specifically focused on dietary lipid composition rather than obesity per se as the primary driver of ferroptotic injury.

We found that PA triggered a ferroptosis-like injury program in the ENS, marked by increased iron import, reduced iron export, elevated labile iron and mitochondrial ROS, GPX4 suppression, lipid peroxidation, and neuronal dysfunction. Transcriptomic changes suggested altered neuronal identity and pathways linked to calcium and synaptic transmission supported by disruption in neuronal excitability and Ca^2+^signaling. In vivo, adeno-associated virus–mediated (AAV-mediated) Nfe2l2 delivery to the myenteric plexus restored antioxidant signaling and improved motility in WD-fed mice, identifying Nfe2l2 as a therapeutic leverage point. Human nhMPG networks showed conserved PA-associated neuronal iron dysregulation, neuronal loss, and glial activation, and our studies identified ferroptosis as an important translational mechanism of diet-related enteric neurodegeneration.

## Results

### PA induces ferroptosis and iron accumulation in enteric neurons.

**Figure 1:**
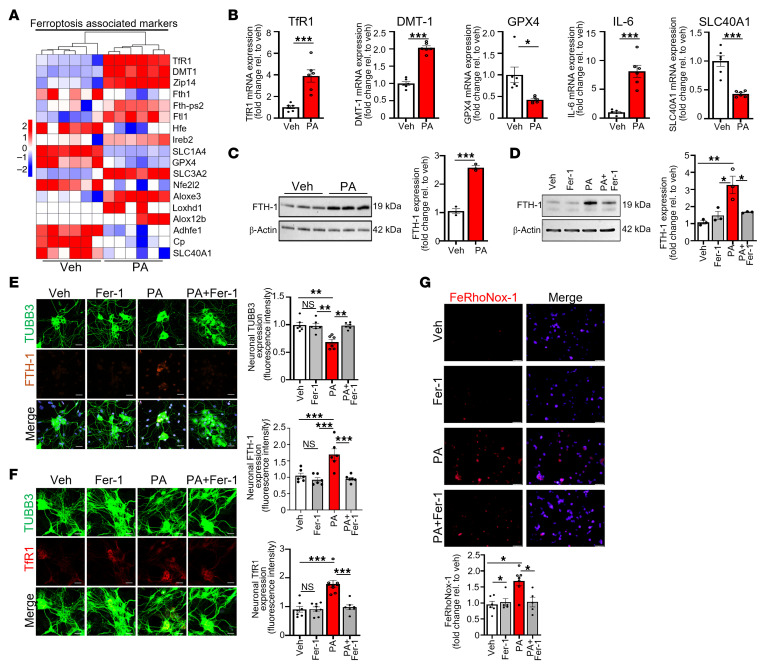
PA induces ferroptosis-associated iron accumulation in enteric neuronal cells. IM-FEN and primary enteric neurons were treated with vehicle (Veh), PA (0.5 mM), Fer-1 (10 μM), or PA+Fer-1 for 24 hours to assess ferroptosis-related iron dysregulation. (**A**) Heatmap of RNA-seq data showing differential expression of ferroptosis- and iron-regulatory genes in enteric neuron cell lines treated with vehicle or PA. (**B**) qRT-PCR analysis of *TfR1*, *DMT1*, *GPX4*, *IL6*, and *SLC40A1* mRNA levels in enteric neuron cell lines treated with vehicle or PA, normalized to *Hprt1*. (**C** and **D**) Western blot analysis of FTH1 protein levels in IM-FEN cells treated with vehicle, PA, Fer-1, or PA+Fer-1; β-actin served as a loading control. (**E**) Immunofluorescence staining for TUBB3 (green) and FTH1 (red) in primary enteric neurons treated with vehicle, PA, or PA+Fer-1. (**F**) Immunofluorescence staining for TUBB3 (green) and TfR1 (red) in primary enteric neurons treated as in **E**, showing that PA-induced TfR1 upregulation was reversed by Fer-1. (**G**) FeRhoNox-1 staining for labile Fe²^+^ (red) with DAPI (blue) in primary enteric neurons treated as in **E**, showing that PA-induced iron accumulation was blocked by Fer-1. Histograms represent the fold change in signal intensity relative to vehicle. Scale bars: 50 μm. Data represents 3 independent experiments. *P < 0.05, **P < 0.01, and ***P < 0.001, by unpaired, 2-tailed *t* test (**B** and **C**) and 1-way ANOVA with Tukey’s multiple-comparison test (**D**–**G**).

To investigate whether PA triggers ferroptotic stress in the ENS, we first evaluated the transcriptional and protein-level changes associated with iron metabolism and ferroptosis in murine enteric neurons. We used PA at 0.5 mM, consistent with our prior work showing that this dose induces enteric neuronal degeneration (20). Also, PA is a major circulating fatty acid, and plasma measurements show that PA concentrations vary widely, from approximately 0.3 mmol/L up to 4 mmol/L, placing 0.5 mM within the physiological range (21). Bulk RNA-seq of IM-FEN cells treated with PA (0.5 mM, 24 hours) revealed a marked upregulation of transferrin receptor 1 (*TfR1*), divalent metal transporter 1 (*DMT1*), and SLC39A14 (*ZIP14*), genes encoding major iron influx transporters (Figure 1A). Simultaneously, *SLC40A1* (ferroportin), which facilitates cellular iron export, was downregulated, indicating a transcriptional shift toward iron retention. These changes suggest that PA promotes intracellular iron loading, a key trigger of ferroptosis.

Quantitative real-time PCR (qRT-PCR) validated these RNA-seq findings, confirming elevated *TfR1* (*P* < 0.001), *DMT1* (*P* < 0.001), and the proinflammatory cytokine *IL6* (*P* < 0.001) as well as reduced *GPX4* (*P* < 0.05) and *SLC40A1* (*P* < 0.001) (Figure 1B). Western blot analysis demonstrated increased protein levels of FTH1 (*P* < 0.001), which stores excess iron (22, 23), and immunofluorescence of primary enteric neurons corroborated enhanced expression of FTH1 (*P* < 0.001) and TfR1 (*P* < 0.001) and reduced expression of the enteric neuronal marker class III β-tubulin (TUBB3) (*P* < 0.01) (Figure 1, C–F). These results collectively indicate that enteric neurons responded to PA by activating iron uptake and storage mechanisms — hallmarks of ferroptotic priming. To determine whether this transcriptional shift results in functional iron overload, we assessed labile Fe²^+^ using FeRhoNox-1 staining. PA increased cytosolic Fe²^+^ (*P* < 0.05) in IM-FEN cells, whereas cotreatment with the ferroptosis inhibitor ferrostatin 1 (Fer-1) reversed this accumulation (*P* < 0.05; Figure 1G). These findings confirm that PA-induced ferroptosis in enteric neurons is initiated through iron dysregulation and is preventable through pharmacological inhibition.

### Ferroptotic stress impairs neuronal identity via oxidative damage and lipid peroxidation.

**Figure 2:**
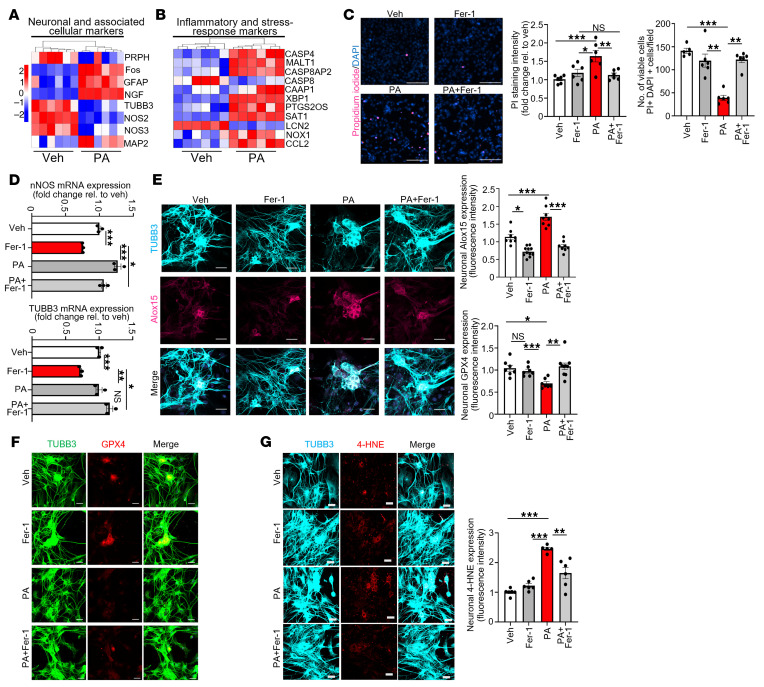
PA triggers ferroptotic stress and loss of enteric neuronal identity via lipid peroxidation. IM-FENs and primary enteric neurons were treated with vehicle, PA (0.5 mM), Fer-1 (10 μM), or PA+Fer-1 for 24 hours to assess ferroptosis-associated cell death, lipid peroxidation, and neuronal identity loss. (**A** and **B**) Heatmaps of RNA-seq data showing differential expression of neuronal and associated cellular markers (**A**) and inflammatory and stress-response markers (**B**) in enteric neurons treated with vehicle or PA. (**C**) Representative images of enteric neurons stained with PI (magenta) and DAPI (blue). Histograms show quantification of PI^+^ nuclei (cell death) and total viable cell counts, indicating PA-induced cytotoxicity reversed by Fer-1. (**D**) qRT-PCR analysis of *nNOS* and *TUBB3* mRNA expression in primary enteric neurons treated as in **C**, normalized to 18s rRNA. (**E**–**G**) Primary enteric neurons were treated as described above. (**E**) Immunofluorescence staining for TUBB3 (cyan) and ALOX15 (magenta). (**F**) Immunofluorescence staining for TUBB3 (green) and GPX4 (red) showing that loss of GPX4 with PA rescued by Fer-1. (**G**) Immunofluorescence staining for TUBB3 (cyan) and 4-HNE (red) showing PA-induced lipid peroxidation that was reversed by Fer-1. Histograms represent the fold change in fluorescence intensity relative to vehicle. Scale bars: 50 μm. Data represent 3 independent experiments. *P < 0.05, **P < 0.01, ***P < 0.001, by 1-way ANOVA with Tukey’s multiple-comparison test (**C**–**G**) and 1-way ANOVA with Tukey’s multiple-comparison test (**D**).

In addition to iron accumulation, we observed substantial neuronal degeneration in PA-treated IM-FEN cells. Bulk RNA-seq revealed that neuronal markers, including *TUBB3* and peripherin (*PRPH*), were downregulated, while oxidative stress–related genes were upregulated (Figure 2, A and B). These transcriptional changes are consistent with reduced expression of neuronal markers and increased expression of stress-related genes, suggesting a shift in molecular signatures under ferroptotic stress. PA exposure led to a dramatic increase in propidium iodide (PI) (*P* < 0.001) incorporation in IM-FEN cells, indicating plasma membrane rupture and cell death. Fer-1 reduced PI uptake (*P* < 0.01), reinforcing the ferroptotic nature of this injury (Figure 2C). Immunofluorescence analysis of primary enteric neurons showed that PA led to elevated levels of Alox15 (*P* < 0.001), a lipid-peroxidizing enzyme that amplifies ferroptotic damage, and concurrently decreased GPX4 (*P* < 0.05), the central antioxidant enzyme preventing lipid peroxide accumulation (12, 24) (Figure 2, E and F). Lipid peroxidation was confirmed by increased 4-hydroxynonenal (4-HNE) (*P* < 0.001) staining in neuronal soma and neurites (Figure 2G). Fer-1 rescued GPX4 expression (*P* < 0.01) and suppressed both 4-HNE accumulation (*P* < 0.01) and loss of neuronal marker expression (nNOS, *P* < 0.05; TUBB3, *P* < 0.05) (Figure 2, D, F, and G). Together, these findings support a model in which PA-induced iron accumulation drives lipid peroxidation, oxidative damage, and loss of enteric neurons through ferroptosis.

### PA impairs mitochondrial integrity and triggers mitochondrial ferroptosis.

**Figure 3:**
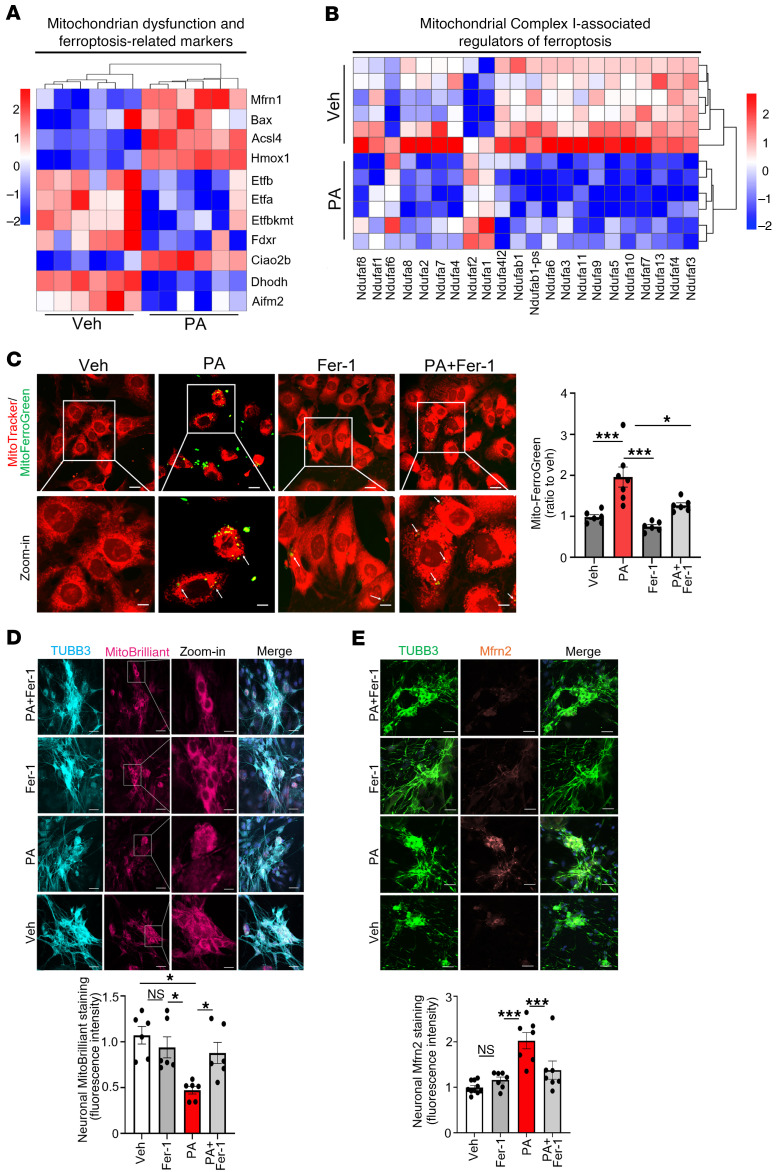
PA drives mitochondrial ferroptosis via ROS accumulation, mitochondrial disruption, and Mfrn2 upregulation. IM-FEN and primary enteric neurons were treated with vehicle, PA, 0.5 mM, Fer-1, 10 μM, or PA+Fer-1 for 24 hours to examine mitochondrial oxidative stress and ferroptosis-related mitochondrial changes. (**A**) Heatmap of mitochondrial dysfunction and ferroptosis-related markers in enteric neurons treated with vehicle or PA. (**B**) Heatmap of mitochondrial complex I–associated regulators of ferroptosis in the same conditions. (**C**) MitoFerroGreen (green) and MitoTracker Red (red) staining in IM-FEN cells showing increased mitochondria-associated labile Fe²^+^ after PA treatment, which is partially reduced by co-treatment with Fer-1; histogram shows fold change in MitoFerroGreen fluorescence colocalized with MitoTracker relative to vehicle. (**D**) Immunofluorescence staining of primary enteric neurons for TUBB3 (cyan) and MitoBrilliant 646 (magenta) with magnified insets showing PA-induced mitochondrial disruption, which was rescued by Fer-1. (**E**) Immunofluorescence staining of primary enteric neurons for TUBB3 (green) and Mfrn2 (brown) showing increased Mfrn2 expression with PA, which was reversed by Fer-1. Histograms in **D** and **E** represent the fold change in fluorescence intensity relative to vehicle. Scale bars: 50 μm. Data represent 3 independent experiments. *P < 0.05, **P < 0.01, and ***P < 0.001, by 1-way ANOVA with Tukey’s multiple-comparison test (**C**–**E**).

To identify components of the ferroptotic stress response, we analyzed gene expression signatures in PA-treated IM-FEN cells. Transcriptomic profiling revealed upregulation of multiple heat shock proteins (HSPs), including Hspa1a, Hspa5, Hsp90aa1, Hspa8, and Hspb1 (Supplemental Figure 2A; supplemental material available online with this article; https://doi.org/10.1172/JCI196113DS1). These molecular chaperones play central roles in protein folding, proteostasis, and the management of cellular stress, including stabilization of mitochondrial and cytosolic proteins under oxidative damage (25). HSPs also indirectly modulate ferroptosis by maintaining mitochondrial function and redox homeostasis (26). Most genes encoding subunits of mitochondrial complex I (NADH:ubiquinone oxidoreductase), including Ndufaf3, Ndufaf4, Ndufab1, Ndufaf7, Ndufa10, and Ndufa4l2, were downregulated (Figure 3B). This suppression suggests mitochondrial dysfunction and impaired electron transport capacity in response to metabolic stress. Given the central role of complex I in initiating oxidative phosphorylation, reduced expression of its components may reflect a collapse in mitochondrial bioenergetics and an adaptive attempt to limit further ROS production under conditions of ferroptotic stress.

Supporting this, PA also reduced the expression of dihydroorotate dehydrogenase (Dhodh) and Aifm2 (also known as FSP1), two critical suppressors of ferroptosis that detoxify lipid peroxides via mitochondrial and plasma membrane CoQ (27, 28) (Figure 3A). These transcriptomic changes were functionally validated by MitoSOX Red staining, which revealed elevated mitochondrial ROS levels in PA-treated IM-FEN cells (*P* < 0.001), and these changes were attenuated by the ferroptosis inhibitor Fer-1 (*P* < 0.01) (Supplemental Figure 9F). In addition, MitoFerroGreen imaging (colabeled with MitoTracker) revealed an increase in mitochondrial labile Fe^2+^ following PA exposure, which was reduced by Fer-1 (Figure 3C), directly linking PA to mitochondrial iron loading during ferroptotic stress. Consistent with these findings, MitoBrilliant 646 imaging showed fragmented mitochondrial networks in primary enteric neurons, consistent with organelle depolarization and dysfunction (*P* < 0.01; Figure 3D). Expression of mitoferrin 2 (Mfrn2), a mitochondrial iron importer (29), was also increased (*P* < 0.001; Figure 3E), suggesting enhanced mitochondrial iron accumulation — a known amplifier of ferroptosis via Fenton chemistry.

Together, these findings indicate that PA triggered ferroptosis in enteric neurons by disrupting mitochondrial respiration, suppressing key ferroptosis inhibitors (FSP1, DHODH), and initiating oxidative and proteotoxic stress responses (via upregulation of HSPs). These transcriptional and functional changes converged on a mitochondria-centered ferroptotic pathway characterized by elevated ROS, iron dysregulation, and mitochondrial injury, which could be partially rescued by ferroptosis inhibition.

### Ferroptosis inhibition separates acute physiological effects from chronic ferroptotic effects of PA on enteric neuronal Ca²^+^ signaling.

**Figure 4:**
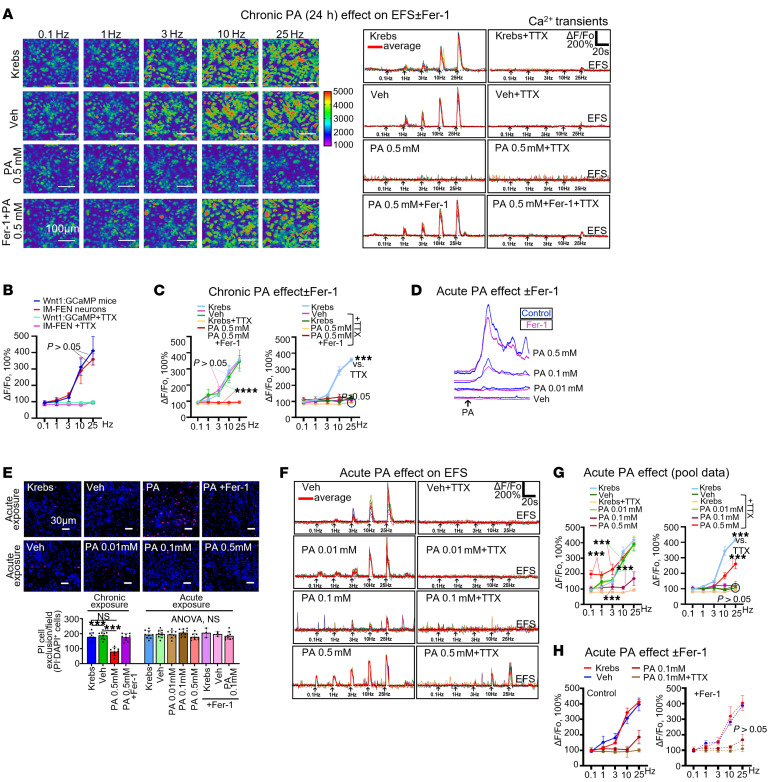
Ferroptotic injury in response to chronic, but not acute, exposure to 0.5 mM PA is sensitive to the ferroptosis inhibitor Fer-1 in IM-FEN enteric neurons. (**A**) Chronic PA exposure abolished Ca^2+^ responses in IM-FEN in response to EFS. A representative example of a frequency-dependent EFS Ca^2+^response is shown in IM-FEN; cells were loaded with the Fluo-4 Ca^2+^ indicator. The Ca^2+^transients are shown in the panel on the right for the various treatments. PA abolished responses and pretreatment with Fer-1 preserved Ca^2+^ signaling. TTX eliminated all EFS activity. (**B**) Identical frequency response Ca^2+^curves were obtained in mouse IM-FEN neurons and neurons in LMMP mouse preparations in Wnt1:GCaMP Ca^2+^reporter mice. (**C**) Pooled data show that chronic exposure (24 hours) to 0.5 mM PA abolished the EFS response in IM-FEN neurons. Fer-1 prevented the chronic effect of PA. EFS responses were blocked by TTX and therefore involved neuronal Na_v_ channels and nerve conduction. (**D**) Acute PA exposure led to direct concentration-dependent increases in neuronal Ca^2+^response from 0.01–0.5 mM. Responses were not sensitive to Fer-1. (**E**) Acute PA exposure (0.01–0.5 mM) did not cause significant neuronal cell death, whereas chronic PA exposure (0.5 mM) caused significant cell death in the IM-FEN population. PI exclusion assay results are shown. (**F**) Differential effects of acute exposure to different concentrations of PA on neuronal activity. PA (0.1 mM) reduced Ca^2+^responses. PA (0.5 mM) augmented EFS responses at lower frequencies of stimulation, and the responses were only partially sensitive to TTX. (**G**) Pooled data for acute PA effects show that PA (0.1 mM) was sufficient to nearly abolish EFS responses, whereas PA (0.5 mM) enhanced responses at low-to-intermediate frequencies, did not block the EFS response, and was associated with a TTX-insensitive component; EFS responses were normally abolished by TTX. (**H**) Fer-1 had no effect on frequency-dependent responses during acute PA exposure (pooled data). *P < 0.05, **P < 0.01, and ***P < 0.001, by 2-way ANOVA for statistical comparisons between curves. ΔF/F_0_, change in fluorescence intensity relative to baseline fluorescence (F_0_).

We next used Ca²^+^ imaging of IM-FEN enteric neurons to distinguish acute physiological effects of PA from chronic ferroptotic injury (Figure 4). Electrical field stimulation (EFS) evoked robust, frequency-dependent Ca²^+^ transients in Fluo-4–loaded IM-FEN cells, and the EFS frequency-response curve closely matched that recorded in neurons of intact LMMP preparations from Wnt1:GCaMP reporter mice, indicating that IM-FEN faithfully reproduced native enteric neuronal activity. Tetrodotoxin (TTX) abolished EFS-evoked Ca²^+^ responses, confirming that these signals depended on Na_v_-mediated action potential conduction (Figure 4, A–C). Chronic exposure to 0.5 mM PA (24 hours) markedly suppressed EFS-evoked Ca²^+^responses and functionally silenced neurons, whereas pretreatment with the ferroptosis inhibitor Fer-1 preserved the EFS frequency-response relationship despite chronic PA exposure (Figure 4, A–C).

Acute PA exposure produced a distinct, nonlethal profile. Short-term perfusion with PA alone (2–10 minutes) induced agonist Ca²^+^transients that were not affected by Fer-1 (Figure 4D) and did not cause measurable cell death by either PI or trypan blue exclusion, in contrast to chronic 0.5 mM PA exposure, which killed more than 50% of IM-FEN neurons in a Fer-1–sensitive manner (Figure 4E and Supplemental Figure 9E). Acute PA also modulated EFS responses in a concentration-dependent manner: 0.1 mM PA strongly inhibited EFS-evoked Ca²^+^ signals, whereas 0.5 mM PA enhanced responses at low-intermediate stimulation frequencies and revealed a TTX-insensitive component (Figure 4, F–H), suggesting recruitment of a TTX-resistant conductance. Fer-1 did not alter these acute effects (Figure 4H). Together, these data identify 2 mechanistically distinct phases of PA action in enteric neurons: a rapid, reversible, Fer-1–insensitive modulation of Ca²^+^ signaling and a delayed, Fer-1–sensitive ferroptotic phase characterized by loss of neuronal activity and cell death.

### PA suppresses phosphorylated Nfe2l2, and AAV-mediated Nfe2l2 overexpression restores redox balance in vivo.

**Figure 5:**
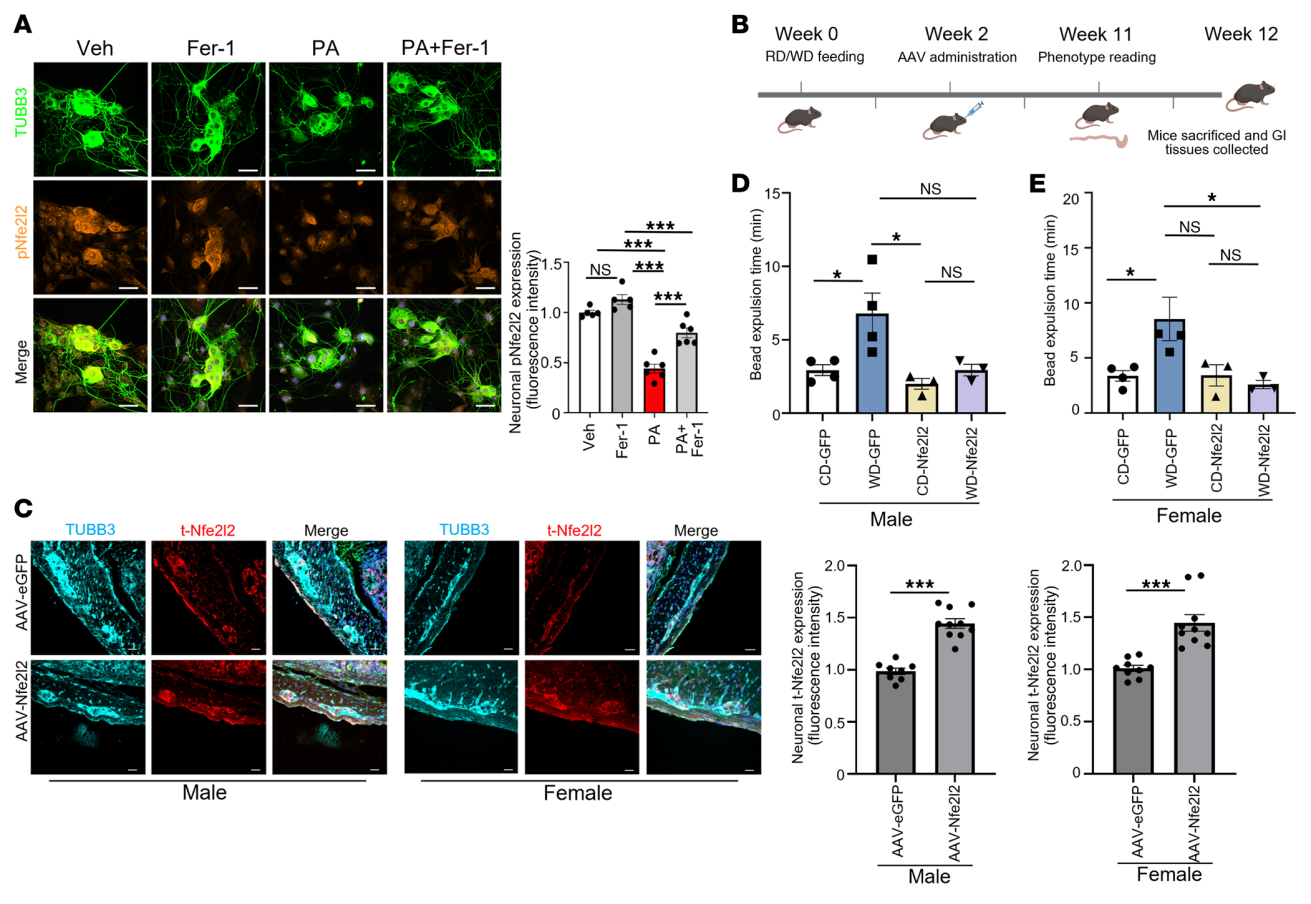
PA suppresses p-Nfe2l2 in enteric neurons, while AAV-mediated Nfe2l2 overexpression restores redox signaling in vivo. To assess the effect of palmitate on Nfe2l2 signaling and the therapeutic potential of AAV-mediated Nfe2l2 overexpression, we performed in vitro and in vivo analyses in enteric neurons and colon tissues. (**A**) Immunofluorescence staining of primary enteric neurons for TUBB3 (green) and p-Nfe2l2 (orange) showing reduced p-Nfe2l2 expression with PA treatment (0.5 mM, 24 hours), rescued by Fer-1 (10 μM). Data represent 3 independent experiments. (**B**) Experimental timeline for in vivo study: male and female mice were fed a CD or a WD for 12 weeks, received retro-orbital AAV-EGFP or AAV-Nfe2l2 at week 2, and were phenotyped at week 11 before tissue collection. (**C**) Immunofluorescence staining of colon sections from CD-fed male and female mice treated with AAV-EGFP or AAV-Nfe2l2, costained for TUBB3 (cyan) and t-Nfe2l2 (red). Merged images show enhanced neuronal Nfe2l2 expression in AAV-Nfe2l2-treated mice. Histogram shows the fold change in t-Nfe2l2 fluorescence intensity within TUBB3^+^ neurons. (**D** and **E**) GI motility measured by bead expulsion time in male and female. *n* = 4 mice for AAV-EGFP groups; *n* = 3 mice for AAV-Nfe2l2 groups. Scale bars: 50 μm. Quantification histograms represent the fold change relative to vehicle or AAV-EGFP group as appropriate. *P < 0.05 and ***P < 0.001, by 1-way ANOVA with Tukey’s multiple-comparison test (**A**), unpaired, 2-tailed *t* test (**C**), and 2-way ANOVA with Tukey’s multiple-comparison test (**D** and **E**).

To determine whether PA disrupts endogenous antioxidant signaling, we assessed phosphorylated Nfe2l2 (p-Nfe2l2) levels in primary enteric neurons. PA treatment reduced nuclear p-Nfe2l2 expression (*P* < 0.001), indicating impaired activation of this redox-responsive transcription factor (30). Bulk RNA-seq further confirmed that *Nfe2l2* mRNA levels were downregulated by PA treatment in IM-FEN cells (Figure 1A). Cotreatment with Fer-1 restored p-Nfe2l2 levels (*P* < 0.001), suggesting that ferroptotic stress directly suppressed Nfe2l2 signaling (Figure 5A). Transcriptomic profiling revealed that PA altered key regulators of lipid metabolism, inflammation, and GI motility. Genes such as *Srebf2*, *Insig1*, *Insig2* (31), and *Nr3c1* (32) were upregulated, and *Ucp2* (33) was downregulated (Supplemental Figure 2B), suggesting a shift toward enhanced lipid and steroid regulatory signaling alongside compensatory and impaired mitochondrial lipid utilization. Concurrently, PA upregulated inflammatory mediators (*Tlr4* [ref. 34], *Myd88*, *Nfkb1* [ref. 35]) and motility-related neuromodulators (*Gabra1* [ref. 36], *Adra2a* [ref. 37], *Cnr1* [ref. 38], *Nts* [ref. 39]) (Supplemental Figure 2C), indicating activation of neuroimmune pathways and inhibitory neurotransmission that may collectively disrupt enteric neuronal function and GI transit. To assess the functional relevance of Nfe2l2 in vivo, we used the AAV-MaCPNS2 capsid, which efficiently transduces peripheral neurons, including enteric neurons, to drive Nfe2l2 expression in adult mice (Figure 5B). Notably, AAV-MaCPNS2 is not selective for myenteric neurons over other peripheral ganglia. We selected a 12-week feeding paradigm because our prior work established this as the earliest time point at which enteric neurodegeneration and delayed colonic transit are detected (40). To confirm successful AAV-mediated gene delivery, we assessed transduction efficiency 1 week after AAV injection (week 3 of the 12-week experiment). EGFP fluorescence was observed in the colonic myenteric plexus by whole-mount confocal imaging, and qRT-PCR of colon tissue enriched for the myenteric plexus confirmed an increase in *Nfe2l2* mRNA expression in AAV-Nfe2l2–treated mice compared with AAV-EGFP controls (Supplemental Figure 3, A and B). At the experimental endpoint (week 12), immunofluorescence staining of colon tissue sections showed sustained upregulation of total Nfe2l2 (t-Nfe212) and p-Nfe2l2 protein levels in enteric neurons, with strong colocalization with the neuronal marker TUBB3 and elevated t-Nfe2l2 and p-Nfe2l2 fluorescence intensity in the AAV-Nfe2l2 group (*P* < 0.001; Figure 5C and Supplemental Figure 13). AAV-mediated overexpression of Nfe2l2 improved colonic motility in WD-fed mice, as assessed by bead expulsion time. WD feeding delayed motility in both sexes compared with controls (*P* < 0.05). Nfe2l2 overexpression prevented the delayed motility induced by a WD in both male and female mice (Figure 5, D and E). These results support a functional role for Nfe2l2 in restoring GI motility under WD-induced metabolic stress. Collectively, these findings demonstrate that WD/PA suppressed Nfe2l2 signaling in enteric neurons, contributing to ferroptosis and motility impairment. AAV-mediated overexpression of Nfe2l2 reestablished redox homeostasis and improved GI function in the context of WD-induced metabolic dysfunction.

### A WD induces ferroptotic signaling and compromises the ENS.

**Figure 6:**
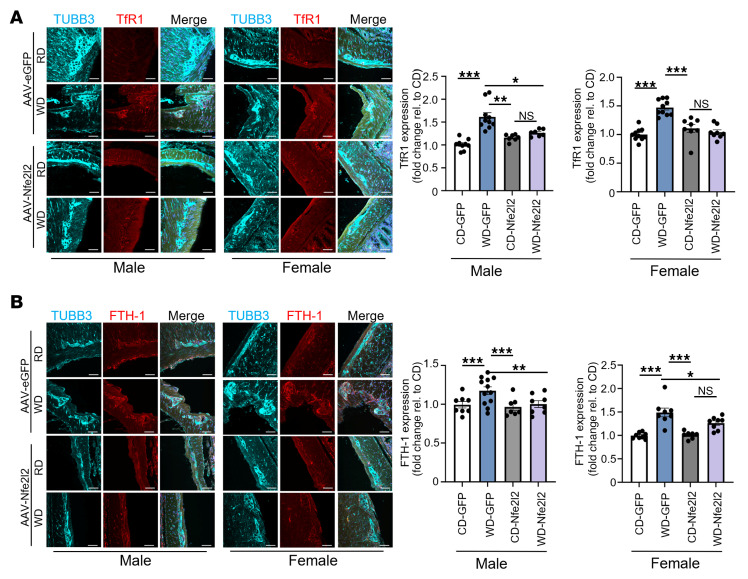
A WD increases transferrin receptor and ferritin levels in myenteric ganglia, mitigated by Nfe2l2 overexpression. (**A**) Immunofluorescence staining of colonic myenteric ganglia for TUBB3 (cyan) and TfR1 (red) in CD-fed and WD-fed mice treated with AAV-EGFP or AAV-Nfe2l2. Histogram shows the fold change in TfR1 fluorescence intensity relative to CD AAV-EGFP (Veh). (**B**) Immunofluorescence staining for TUBB3 (cyan) and FTH1 (red) in the same groups. Histogram shows the fold change in FTH1 fluorescence intensity relative to CD AAV-EGFP–treated mice (Veh). A total of 28 mice were analyzed: *n* = 4 per group for CD and WD AAV-EGFP–treated mice, and *n* = 3 per group for CD and WD AAV-Nfe2l2–treated mice. For each mouse, 6–10 randomly selected myenteric ganglia were imaged and quantified. Scale bars: 50 μm. *P < 0.05, **P < 0.01, and ***P < 0.001, by 2-way ANOVA with Tukey’s multiple-comparison test.

To assess the long-term effects of a WD on enteric neuronal ferroptosis, we analyzed colonic tissues from mice fed either a control diet (CD) or a WD for 12 weeks. AAV vectors (EGFP or Nfe2l2) were administered at week 2 to enable targeted modulation of antioxidant signaling in enteric neurons. WD-fed mice exhibited the characteristic pattern of progressive weight gain over time compared with CD-fed controls, confirming progressive diet-induced obesity (Supplemental Figure 2, D and E). Immunofluorescence analysis revealed that WD exposure notably increased expression of TfR1 (*P* < 0.001) and ferritin heavy chain 1 (FTH1) (*P* < 0.001) within myenteric ganglia compared with CD-fed controls (Figure 6, A and B), indicating chronic iron accumulation and sustained ferroptotic stress. These changes mirror those observed in PA-treated enteric neurons (Figure 1) and suggest that dietary lipotoxicity activated ferroptotic programs in vivo. Notably, AAV-Nfe2l2 overexpression markedly suppressed both TfR1 (male, *P* < 0.05; female, *P* < 0.001) and FTH1 (male, *P* < 0.01; female, *P* < 0.05) expression, reinforcing the role of Nfe2l2 in regulating neuronal iron metabolism and mitigating ferroptotic signaling.

Concurrently, we observed suppression of key neuron- and redox-associated markers in WD-fed animals. Neuronal NOS (nNOS) — a critical enzyme for nitrergic neurotransmission and gut motility — was significantly downregulated (male, *P* < 0.05; female, *P* < 0.01) in WD-fed mice, indicating neuronal dysfunction (Supplemental Figure 4). Collectively, these findings demonstrate that chronic consumption of a WD exacerbates iron overload and ferroptosis-associated molecular injury in the ENS, while simultaneously impairing nitrergic signaling and antioxidant defense. Importantly, AAV-Nfe2l2 gene therapy both limited ferroptotic stress and partially restored neuronal function, highlighting its translational potential as a neuroprotective strategy in the context of metabolic stress–induced enteric neuropathy.

To assess whether ferroptotic stress markers localize to specific enteric neuronal subtypes, we colabeled myenteric ganglia for TUBB3 with nNOS, choline acetyltransferase (ChAT), or tyrosine hydroxylase (TH), together with 4-HNE and dynamin-related protein 1 (DRP1) (Supplemental Figures 10–12). A WD increased 4-HNE and DRP1 signals across all 3 neuronal populations, without a consistent subtype hierarchy. The clearest subtype-associated separation in vivo was observed in female ChAT^+^ neurons. In parallel, PA exposure in primary ENS cultures induced a comparable 4-HNE and DRP1 stress signature across nNOS^+^, ChAT^+^, and TH^+^ neurons (Supplemental Figure 8C). Fer-1 attenuated these PA-induced changes, supporting lipid peroxidation as a central component of the neuronal stress response. Together, these data indicate that WD and PA engage broadly shared ferroptotic stress across myenteric neuronal subtypes, while subtype-selective vulnerability likely reflects downstream sensitivity rather than differential induction of these markers.

### PA-associated ferroptosis modulates transcripts in synaptic and calcium signaling pathways in enteric neurons.

To assess how PA-associated ferroptosis might influence neuronal signaling programs, we performed bulk RNA-seq on IM-FEN cells treated with PA or vehicle. Transcriptomics analysis revealed that genes annotated to calcium signaling and synaptic neurotransmission pathways were modulated following PA treatment (Supplemental Figure 1, A–D). The purinergic receptor P2rx6, a key mediator of calcium influx (41), was downregulated, suggesting potential alterations in calcium handling under oxidative stress. Presynaptic genes essential for vesicle docking and release, including *Rab3a* (42) and *Stx1b* (43), also showed reduced expression, and glutamatergic signaling components such as *Grm3* and *Grm5* (44) were downregulated.

These transcriptomic changes preceded overt cell death and likely represent early stress-responsive remodeling of neuronal signaling pathways during ferroptotic stress. We interpret these findings as hypothesis-generating signatures that point to potential alterations in calcium handling and synaptic programs, rather than definitive evidence of functional impairment. Indeed, the calcium imaging studies described earlier (Figure 4) support the hypothesis that PA-induced transcriptional changes may underlie functional impairment in the ENS and motility. Future studies are needed to test this hypothesis.

### Human myenteric neurons exhibit conserved ferroptotic responses to PA.

To determine whether PA-induced ferroptotic mechanisms observed in murine models are conserved in the human ENS, we freshly isolated networks of intact nhMPG from 14 surgical intestinal specimens from patients undergoing elective colectomy. These neural networks, an in vitro model of the human ENS, were treated with PA (0.5 mM for 24 hours) and analyzed using high-resolution confocal imaging to evaluate ferroptosis.

Patient metadata are summarized in Supplemental Table 4, including sex, age, BMI (mean, 33.18 ± 6.74), diabetes status, clinical lab values (iron indices, lipid profile, hemoglobin A1c [HbA1c], C-reactive protein [CRP]), medications, and surgical indications. Patients had diverse metabolic and inflammatory profiles. Some were obese, had type 2 diabetes (T2D), or exhibited impaired fasting glucose (IFG); others had normal metabolic panels. A subset showed altered systemic iron metabolism, e.g., 1 patient with high ferritin levels, another with elevated transferrin saturation, and a few had elevated CRP levels, indicating low-grade inflammation. Despite this variability, all nhMPG preparations exhibited PA-induced ferroptotic phenotypes, underscoring the generalizability of this mechanism in the human colon.

After standardization of the isolation protocols (Supplemental Figure 5A), nhMPG networks were maintained under optimized conditions in organotypic culture medium in a humidified 5% CO_2_ chamber to permit treatment with PA or other agents, thereby preserving ganglionic integrity and functionality. Networks remained suspended in medium during PA exposure, minimizing any glial/cell differentiation unrelated to PA induction. Transmitted light imaging showed well-preserved architecture and diverse morphologies across patients, with clearly delineated ganglia and neurite projections (Supplemental Figure 5B).

**Figure 7:**
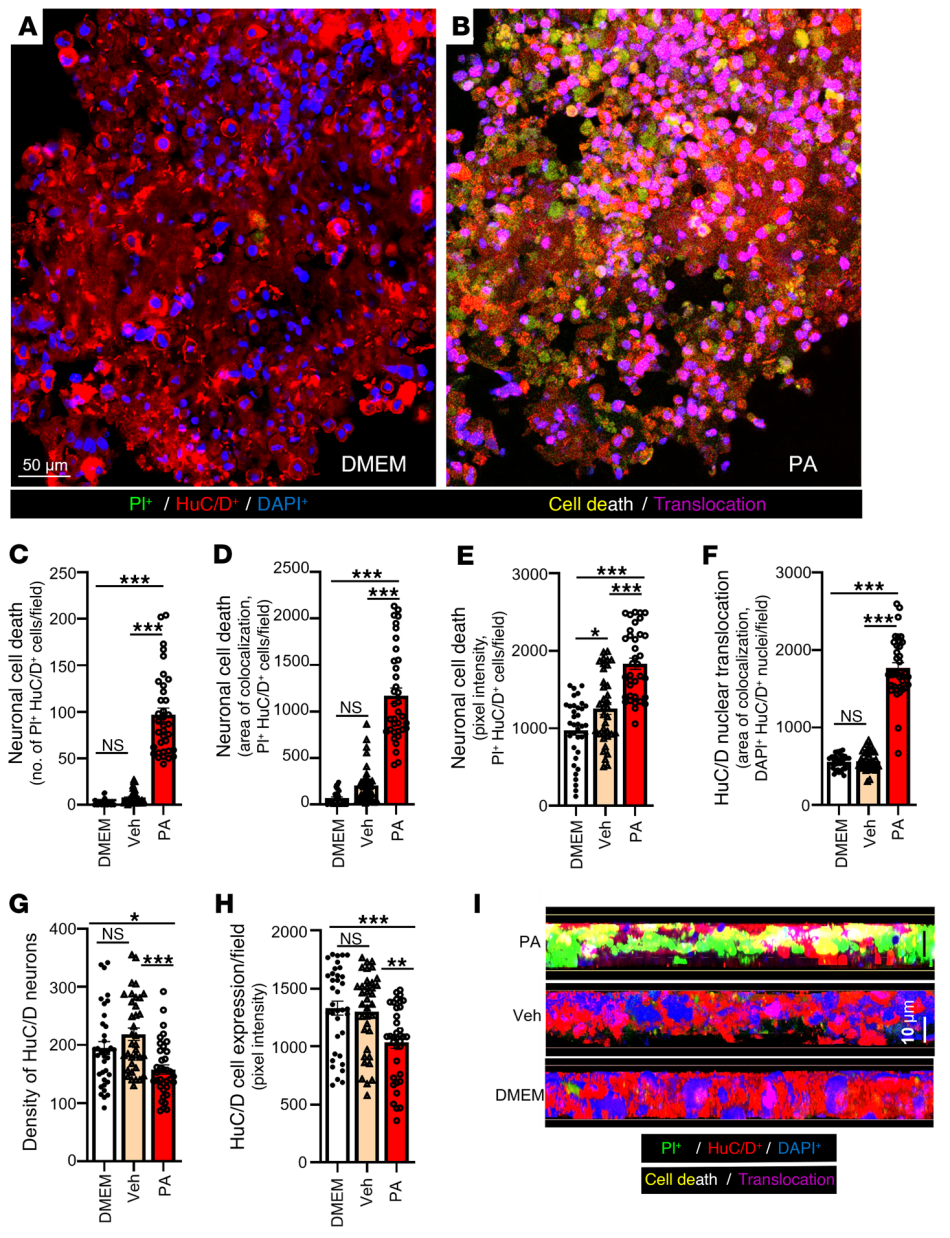
PA increases cell death in isolated nhMPG from colectomy surgical specimens. nhMPG were isolated from colectomy surgical specimens and treated with PA or vehicle (DMEM) ex vivo. (**A** and **B**) PA (0.5 mM) increased PI fluorescence in HuC/D^+^ nuclei/field (counterstained with DAPI) compared with DMEM in nhMPG. (**C**–**E**) PA showed significant increases in PI^+^ neurons/field, PI intensity in neurons/field, and the PI^+^ area colocalized in each neuron. (**F**) PA increased the nuclear translocation of HuC/D protein, a sign of stress on the neurons. Neuronal translocation was analyzed by colocalization of HuC/D^+^ immunoreactivity and DAPI in nuclei. (**G**) PA also decreased the density of neurons as well as the intensity of HuC/D immunoreactivity (**H**) in neurons. (**I**) Cross-sections of *Z*-stack images were used to further illustrate the effect of PA on increasing neuronal cell death and nuclear translocation of HuC/D. NIS Elements colocalization software was used to quantify cell death, translocation, TfR1 activation, and FTH1 activation in 18 μm thick confocal *Z*-stacks captured at 0.5 μm optical sections (*n* = 36 different networks analyzed for each treatment). Data were analyzed from gut surgical specimens procured from 3 patients for each parameter; 12 *Z*-stacks in different networks of ganglia (nhMPG networks) in each patient were used for colocalization analysis and statistics. Data indicate the mean ± SEM. *P < 0.05, **P < 0.01, and ***P < 0.001, by 1-way ANOVA with Tukey’s multiple-comparison test. Scale bars: 50 μm (**A** and **B**); 10 μm (**I**).

Upon PA exposure, we observed widespread cell death in pan-neuronal RNA-binding proteins HuC and HuD (HuC/D^+^ neurons), marked by robust PI uptake compared with vehicle-treated controls (Figure 7, A–D). Quantitative analysis revealed a 12.1-fold increase in PI^+^/HuC/D^+^ nuclei per field, a 5.5-fold increase in colocalization area, and a 1.46-fold increase in PI fluorescence intensity (*P* < 0.001; Figure 7E). PA also triggered nuclear translocation of HuC/D (Figure 7F), a known stress marker, and caused a 28% reduction in neuronal density (*P* < 0.001; Figure 7G). Notably, while HuC/D fluorescence intensity declined slightly in some fields (Figure 7H), this may reflect complete loss of heavily damaged neurons. *Z*-stack cross-sections demonstrated pronounced architectural disruption and fragmented nuclei (Figure 7I), mirroring murine data and validating the ferroptotic phenotype in human tissue (Supplemental Figure 14).

### TfR1 is upregulated in neuronal and non-neuronal compartments of human nhMPG networks following PA, FAC, and LPS exposure.

**Figure 8:**
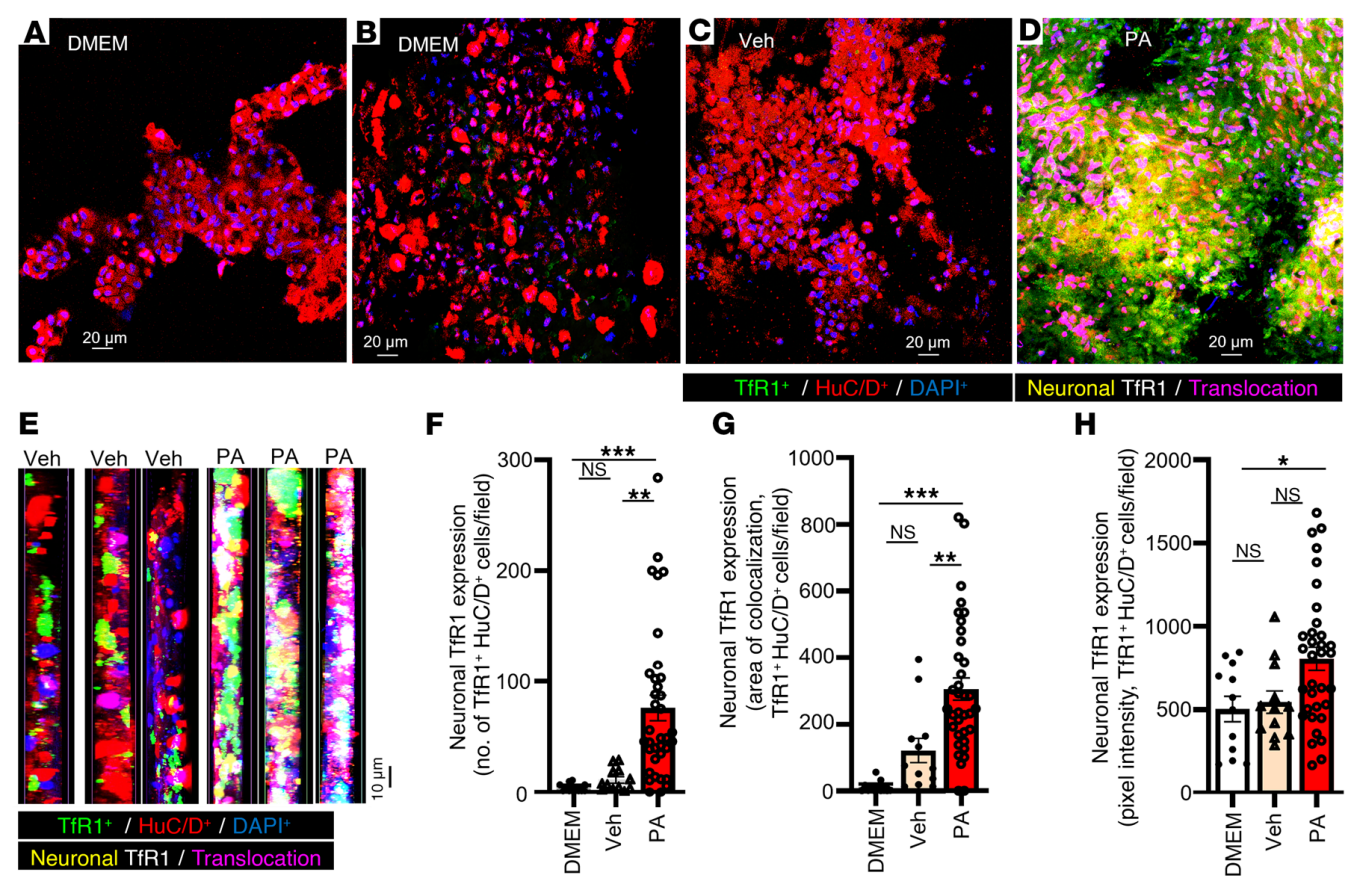
PA induction of TfR1 expression in human myenteric neurons of nhMPG networks. nhMPG isolated from surgical specimens were treated ex vivo with PA, DMEM, or vehicle control to assess neuronal TfR1 expression. (**A**–**E**) PA increased TfR1 expression in HuC/D^+^ neurons of nhMPG networks, compared with DMEM or vehicle control. (**E**) Images shown are cross-sections of *Z*-stack images through the networks of ganglia to further illustrate neuronal TfR1. (**F**–**H**) PA caused an increase in the number of neurons/fields that expressed TfR1, the area of colocalization of TfR1, and HuC/D^+^ neurons/field, and it increased TfR1 expression (pixel intensity/field). Data indicate the mean ± SEM. *P < 0.05, **P < 0.01, and ***P < 0.001, by 1-way ANOVA with Tukey’s multiple-comparison test. Scale bars: 20 μm (A–D) and 10 μm (**E**).

Given the centrality of iron in ferroptosis, we next analyzed a prominent marker of ferroptosis, TfR1,which transports iron into cells, resulting in elevated labile iron levels that can potentially cause toxicity (45). TfR1 and colabeling studies evaluated the effect of PA on TfR1 expression in human enteric ganglia. PA increased TfR1 expression in HuC/D^+^ neurons of nhMPG networks compared with DMEM or vehicle control (with BSA; Figure 8, A–E); Data analysis showed that PA caused an increase in the number of neurons/field that expressed TfR1 (Figure 8F; *P* < 0.01), and it increased the area of colocalization of TfR1 and HuC/D^+^ neurons/field (Figure 8G; *P* < 0.01). The expression of TfR1 (pixel intensity) for TfR1 immunoreactivity in neurons/field was also increased by PA treatment (Figure 8H; *P* < 0.05, Supplemental Figure 15).

To contextualize this response, we compared PA with known ferroptosis inducers such as ferric ammonium citrate (FAC), a strong stimulus for induction of ferroptosis (46), and observed a huge upregulation of TfR1 in neurons of nhMPG networks (Supplemental Figure 6, A–D). The bacterial membrane toxin LPS (1 μg/mL) also caused TfR1 upregulation in enteric neurons (Supplemental Figure 6, E–G). We conducted secondary data analysis to compare PA-, LPS-, and FAC-induced TfR1 expression in nhMPG networks. Data are summarized in Supplemental Figure 6, H and I. A concentration of 0.5 mM PA (*P* < 0.05) or 1 μg/mL LPS induction (*P* < 0.05) for 24 hours caused the same level of upregulation in neuronal TfR1 expression levels/field (*P >* 0.05 for the difference). FAC caused a several-fold higher level of TfR1 expression in neurons/field compared with LPS or PA (Supplemental Figure 6H). PA induced TFR1 expression in nhMPG networks in approximately 20% of the neuronal population induced with FAC (FAC >> PA, *P* < 0.001). In contrast, a secondary analysis of non-neuronal TfR1 expression (based on indirect analysis of TfR1 expression in areas not colabeled with the neuronal marker HuC/D) showed that PA induction of TfR1 in non-neuronal cells >> FAC (*P* < 0.001), and differences were observed for both total area/field labeled for TfR1 (Supplemental Figure 6I) or the number of different areas/field labeled for TfR1 (*P* < 0.001, Supplemental Figure 6J) immunoreactivity. We found that pixel intensity was marginally but significantly higher with FAC > PA (*P* < 0.001, Supplemental Figure 6K).

### PA induction of FTH1 expression in human myenteric neurons of nhMPG networks.

**Figure 9:**
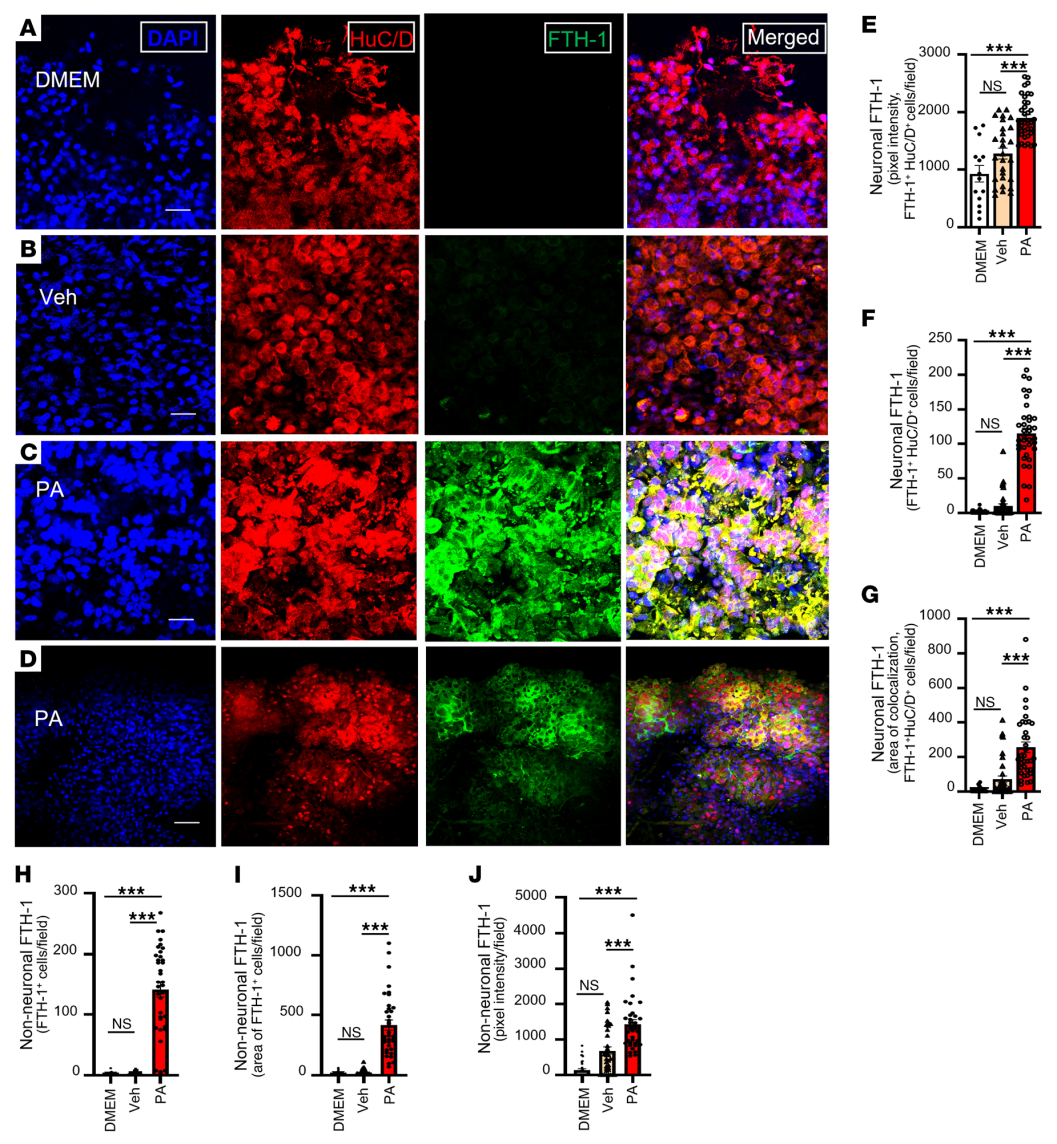
PA induction of FTH1 expression in human myenteric neurons of nhMPG networks. (**A**–**D**) nhMPG networks were exposed to DMEM, vehicle, or palmitate for 24 hours and stained for FTH1. (**E**–**G**) Summarized pooled data showing significant upregulation of FTH1 in neurons with an increase in the number of HuC/D^+^ neurons expressing FTH1, an increase in expression/neuron (intensity), and an associated increase in the area of colocalization of FTH1 with HuC/D^+^ neurons. (**H**–**J**) Secondary analysis showed that non-neuronal FTH1 levels were also significantly elevated in nhMPG networks, suggesting that FTH1 upregulation was not restricted to enteric neurons in response to PA. Data indicate the mean ± SEM. ***P < 0.001, by 1-way ANOVA with Tukey’s multiple-comparison test.

To further assess iron-handling responses in the human ENS under lipid stress, we measured the expression of FTH1, the primary intracellular iron storage protein and a key ferroptosis marker. Following 24 hours of PA treatment, we found that FTH1 was robustly upregulated in human nhMPG networks. Immunofluorescence analysis revealed an 11.3-fold increase in the number of FTH1^+^/HuC/D^+^ neurons per field and a 1.5-fold increase in average signal intensity within these neurons (*P* < 0.001; Figure 9, A–G). Morphologically, FTH1 localized prominently within the somatic cytoplasm and proximal neurites of stressed neurons.

Importantly, FTH1 expression was also elevated in non-neuronal regions of the network, including glial-like territories not colabeled with HuC/D. Quantification showed an increase in FTH1 signal area, number of labeled regions, and intensity across these compartments (*P* < 0.001; Figure 9, H–J), indicating that the iron sequestration response was not restricted to neurons but extended throughout the ENS microenvironment. This widespread FTH1 upregulation may represent an adaptive buffering mechanism against rising intracellular labile iron (Fe^2+^) but also reflects ongoing iron dysregulation and ferroptotic pressure. These findings parallel our murine data, reinforcing FTH1 as a robust marker of ferroptotic stress in both neurons and glia (Supplemental Figure 16).

### PA-induced disruption of morphology and activation of glial fibrillary acidic protein in nhMPG networks.

In addition to the neuronal ferroptosis seen in all 14 patients, we sought to determine whether glial cells and network architecture are affected by PA treatment. Although most nhMPG networks retained structural integrity, 2 of the 14 patient-derived preparations exhibited overt ganglionic disorganization and morphological collapse following PA exposure. Costaining with HuC/D, DAPI, and PI revealed loss of organized ganglionic boundaries, fragmented neuronal nuclei, and diffused PI signal, indicative of both necrotic and ferroptotic processes (Supplemental Figure 7, A and B). This observation suggests that ferroptosis may progress in some cases from isolated cellular injury to structural breakdown of the entire ganglionic unit.

To evaluate glial stress responses, we analyzed expression levels of glial fibrillary acidic protein (GFAP), a hallmark of reactive gliosis. GFAP is typically absent or minimally expressed in healthy human enteric glia but is strongly induced under inflammatory or degenerative conditions. Following PA treatment, GFAP was clearly upregulated in enteric glial regions of the nhMPG (*P* < 0.001; Supplemental Figure 7C), indicating activation of gliotic remodeling programs in response to lipid-induced stress. Together, these findings demonstrate that ferroptotic injury in the human ENS extended beyond neuronal death to include glial reactivity and, in more severe cases, network-level disruption. This dual cellular involvement highlights the broader pathogenic effect of saturated lipid overload on ENS integrity and function.

## Discussion

**Figure 10:**
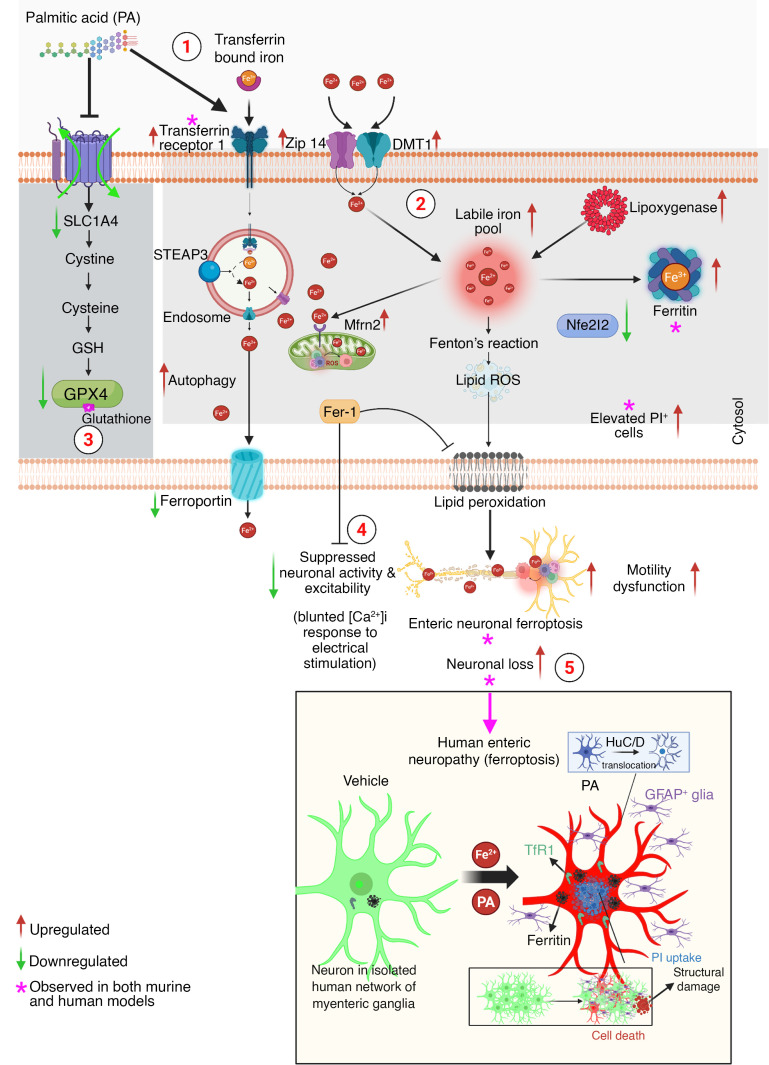
PA induces ferroptotic enteric neurodegeneration through iron accumulation, oxidative stress, and mitochondrial dysfunction in murine and human models. Schematic summary of the proposed mechanism by which chronic PA, a major dietary SFA, drives ferroptotic injury in enteric neurons, integrating findings from murine models and nhMPG. Steps: (i) Chronic PA exposure. (ii) Increased iron uptake and trafficking through upregulation of TfR1, DMT1, and ZIP14, expanding the intracellular labile Fe^2+^ pool and promoting iron retention. (iii) Impaired antioxidant defense via suppression of cystine transport and the GSH/GPX4 axis, which further sensitizes neurons to oxidative injury. (iv) Functional ENS impairment, shown as suppressed neuronal activity and excitability [blunted intracellular free calcium ([Ca^2+^]i) response to electrical stimulation]. (v) Convergent iron-driven oxidative stress and lipid peroxidation culminate in enteric neuronal ferroptosis and cell death, leading to neuronal loss and motility dysfunction. Fer-1 inhibits lipid peroxidation and preserves neuronal activity and excitability, mitigating ferroptotic cell death. Created with BioRender (2025).

This study defines PA- and WD-induced ferroptosis as a principal, conserved mechanism of enteric neurodegeneration, with direct relevance to GI dysfunction observed in metabolic diseases such as diet-induced obesity. By leveraging murine models, in vitro enteric neuronal cultures, and nhMPG, we delineated a ferroptotic cascade characterized by iron dyshomeostasis, mitochondrial dysfunction, lipid peroxidation, and neuronal degeneration culminating in neurodegeneration of the ENS (Figure 10). While ferroptosis is well characterized in the CNS, particularly in stroke (47) and neurodegeneration models (48), its role in the ENS has been largely unexplored. The importance of our study lies in filling this critical knowledge gap: identifying ferroptosis as a major pathophysiological mechanism of neurodegeneration in the ENS and establishing direct links between dietary lipid stress (PA and WD) and ferroptotic injury. Given the ENS’s pivotal role in regulating GI motility and homeostasis and the increasing incidence of GI complications in metabolic diseases, we believe these findings have substantial clinical and translational relevance. We therefore examined mice after 12 weeks on a WD, when colonic neurodegeneration and motility delay are established, to isolate diet-driven ferroptotic mechanisms. In vivo, dietary PA was absorbed and metabolized, and the WD-induced enteric neurodegeneration likely reflects the combined actions of multiple circulating lipids and metabolites rather than PA alone. Thus, direct PA exposure in vitro served as a reductionist model to define neuron-intrinsic ferroptotic mechanisms of WD-associated SFAs, whereas WD feeding established in vivo relevance in the full metabolic and microbial context.

We observed that core ferroptotic processes such as iron overload, mitochondrial dysfunction, lipid peroxidation, and antioxidant failure were conserved between the CNS and ENS, confirming ferroptosis as a broadly conserved mechanism of neuronal injury. Consistent with its well-established cytoprotective role in CNS disorders, FTH1 is traditionally considered cytoprotective by buffering labile Fe²^+^ (49); our findings in the ENS highlight a more nuanced and context-specific regulation of FTH1 in the setting of PA-induced ferroptosis. Although canonical *FTH1* mRNA expression was modestly downregulated in our model, FTH1 protein levels were robustly elevated. This apparent disconnect may be explained by upregulation of *FTH1-ps2*, a pseudogene known to act as a competing endogenous RNA (ceRNA), stabilizing *FTH1* mRNA by sequestering inhibitory miRNAs (50). In addition, iron-regulatory proteins (IRPs) may facilitate translational derepression of FTH1 under iron overload conditions (51). Therefore, the observed FTH1 protein upregulation likely reflects a stress-adaptive response to excess iron rather than a protective mechanism sufficient to prevent ferroptosis. Notably, ferritin induction does not universally confer ferroptosis resistance, and under certain conditions, ferritin instability or ferritinophagy can paradoxically exacerbate oxidative stress. Thus, FTH1 upregulation in our model may have signaled ferroptotic stress without necessarily abrogating it. Beyond its role in lipotoxicity and ferroptosis, PA also serves as a substrate for S-palmitoylation, a reversible lipid modification that regulates the localization and function of many neuronal proteins. Although palmitoylation was not assessed here, PA-driven changes in protein palmitoylation may represent an additional mechanism contributing to ENS dysfunction in high-fat states and will require dedicated investigation.

Prior studies have reported that enteric glia outnumber neurons by approximately 7:1, providing important structural context (52). In our experimental models, enteric glia were highly susceptible to ferroptotic stress, as evidenced by TfR1 upregulation and de novo GFAP expression following PA exposure. Glial ferroptosis likely amplifies neuronal injury, highlighting neuron-glia crosstalk as a critical driver of ferroptotic damage in the ENS. In epilepsy, reactive astrocytes can modulate neuronal ferroptosis through chemokines; for example, astrocyte-derived CXCL10 acting via CXCR3 promotes neuronal ferroptosis (53). These CNS data support the broader concept that diverse reactive glial states, rather than discrete A1/A2 categories, can shape neuronal vulnerability to ferroptosis, a framework that is likely to extend to enteric glia as well (54). Astrocytes in the setting of inflammation can trigger ferroptosis in neurons by releasing chemokines and cytokines like IL-6, which in turn can induce the expression of proteins like ferroportin in neurons, leading to ferroptosis. In addition, astrocytes through Nfe2l2 can counter neuronal ferroptosis by releasing exosomes (55). The interaction between glia and neurons in the enteric nervous system needs to be further examined. Interestingly, although Nfe2l2 is canonically known to transcriptionally activate FTH1 (56), we observed a reduction in FTH1 protein levels upon Nfe2l2 overexpression during WD exposure. This does not imply direct repression of ferritin genes, but rather suggests that Nfe2l2 activation limited the upstream ferroptotic stimuli, namely iron influx and oxidative stress, that drive compensatory FTH1 induction. By restoring iron and redox homeostasis, Nfe2l2 indirectly reduced the cellular need for ferritin-based iron sequestration. This context-dependent regulation reinforces Nfe2l2’s role in suppressing ferroptotic signaling and highlights its potential therapeutic relevance in the ENS.

Our study provides several key findings: (a) identification of PA and WD as potent inducers of ferroptosis-driven enteric neurodegeneration in both the murine and human ENS; (b) demonstration that Nfe2l2 activation restores redox balance, suppresses iron burden, preserves nitrergic neuronal identity (nNOS), and improves GI motility under WD stress. Consistent with this finding, p-Nfe2l2 immunostaining in colonic myenteric neurons confirmed effective pathway activation after AAV-Nfe2l2 administration, and subtype-specific analyses showed broad induction of mitochondrial and ferroptotic stress across nNOS^+^, ChAT^+^, and TH^+^ neurons; and (c) cross-species validation that human nhMPG networks had conserved ferroptotic signatures, including neuronal death, TfR1/FTH1 upregulation, HuC/D nuclear translocation, and glial activation. Dose-response experiments in nhMPG further showed graded induction of neuronal TfR1 and FTH1, together with progressive neuronal loss after PA exposure, indicating that SFA-driven iron loading and ferroptotic vulnerability were quantitatively conserved in human enteric neurons. Furthermore, we identified enhanced mitochondrial iron import via upregulation of Mfrn1 and Mfrn2 in enteric neurons, a distinct mechanism contributing to ferroptotic vulnerability through mitochondrial Fe²^+^ accumulation and oxidative stress.

Mechanistically, bulk RNA-seq of IM-FEN neurons exposed to PA revealed upregulation of iron importers (TfR1, DMT1, ZIP14) and downregulation of ferroportin (SLC40A1), leading to labile Fe^2+^ accumulation, as visualized by FeRhoNox-1 fluorescence. Despite FTH1 upregulation, iron buffering was insufficient to prevent lipid peroxidation, as evidenced by increased 4-HNE levels. PA treatment downregulated mitochondrial complex I subunits (Ndufa2, Ndufa4, Ndufa3) and antioxidant enzymes (Dhodh, FSP1), leading to elevated mitochondrial ROS production and disrupted membrane potential. Upregulation of Mfrn1 and Mfrn2 further facilitated mitochondrial iron import, exacerbating oxidative stress and ferroptotic injury. ER stress genes (*Hspa1a*, *Hspa5*, *Hsph1*) were concurrently induced, supporting a model of multiorganelle dysfunction. This pattern supports a biphasic response in which PA initially induced adaptive stress programs, including HSP pathways, but sustained exposure overwhelmed GPX4- and Nfe2l2-dependent defenses so that residual protection was insufficient to prevent the progression of ferroptosis. A complementary PA time course (4, 8, 12, and 24 hours) in enteric neurons showed that early induction of TfR1 and IL-6, together with loss of FSP1 and GPX4, preceded major loss of viability, indicating that a 24-hour PA exposure captured a mechanistically relevant ferroptotic endpoint rather than late, nonspecific cell death. The integration of MitoFerroGreen/MitoTracker imaging, C11-BODIPY lipid peroxidation assays, and 4-HNE/DRP1 immunostaining links these transcriptional changes to mitochondrial Fe²^+^ loading, membrane damage, and structural stress, while Ca²^+^ imaging shows that chronic PA exposure converts early, reversible changes in excitability into a delayed, ferroptosis-associated loss of neuronal function. In vivo, AAV9-mediated overexpression of Nfe2l2 suppressed WD-induced iron overload, preserved nNOS^+^ neuronal populations, and restored the colonic transit time, demonstrating the therapeutic potential of enhancing antioxidant defenses. These protective effects are supported by increased p-Nfe2l2 staining in distal colonic myenteric neurons after AAV-Nfe2l2 treatment. Human nhMPG networks from diverse patients recapitulated murine findings, with PA-induced neuronal death, nuclear HuC/D translocation, TfR1/FTH1 upregulation, and glial GFAP induction, underscoring the clinical relevance of ferroptosis in human enteric neuropathy. Together, the murine and human datasets converge on a coherent ferroptotic signature that spans iron import, lipid peroxidation, mitochondrial injury, transcriptional remodeling, and functional neuronal failure.

In this study, bead expulsion testing and all histological and molecular analyses were performed on distal colonic segments, aligning functional and mechanistic readouts while acknowledging that mitochondrial and ferroptotic changes may vary along the GI tract and will require future regional mapping. Although our murine and ex vivo models recapitulated key features of ferroptosis, in vivo complexity involving microbiota, immune modulation, and epithelial interactions remains to be explored. In this context, WD-driven dyslipidemia, low-grade mucosal inflammation, immune cell and glial activation, and microbiota-derived metabolites are best viewed as converging inputs that collectively amplify oxidative and iron stress within the ENS rather than as isolated parallel pathways. Prior work in this WD model has already shown broad shifts in triglyceride species, bile acids, and microbial products, and these additional metabolites may further modulate ferroptotic susceptibility and will require targeted metabolomics in future studies (40). In addition, future studies will investigate how fat malabsorption and disrupted fluid drainage in the GI tract influence ferroptotic susceptibility and contribute to ENS dysfunction. Although our WD and human nhMPG data show glial iron loading, GFAP induction, and network disruption, the current study does not dissect causal neuron-glia interactions in ferroptosis or motility, which will require glia-targeted ferroptosis manipulation, neuron-glia coculture systems, and spatial transcriptomics in WD-fed mice. Longitudinal human sampling, metabolic phenotyping, and bulk and single-cell transcriptomics of purified nhMPG networks will be essential to dissect cell-type–specific vulnerabilities. Electrophysiological recordings may further elucidate functional impairments within the enteric network during ferroptotic stress.

In conclusion, this study identifies ferroptosis as a principal, conserved yet distinct mechanism of enteric neurodegeneration triggered by PA- and WD-induced lipid stress leading to abnormal motility. We demonstrated that dietary lipotoxicity disrupted iron homeostasis, impaired mitochondrial antioxidant defenses, promoted lipid peroxidation, and induced enteric neuronal and glial death in the murine and human ENS. Enhanced mitochondrial iron loading and glial ferroptotic susceptibility emerge as unique amplifiers of injury in the ENS. Therapeutic activation of Nfe2l2 via AAV gene therapy represents a promising strategy to mitigate ferroptotic injury and preserve GI function. These rescue experiments indicate that enhancing Nfe2l2 signaling is sufficient to reverse key WD-induced ferroptotic changes, but do not imply that Nfe2l2 downregulation is the sole mediator of pathology. We believe these findings provide critical mechanistic insight into enteric neuropathy in metabolic diseases and establish ferroptosis as a novel, translationally relevant and potential therapeutic target in human ENS disorders associated with abnormal motility.

## Methods

### Sex as a biological variable.

Experiments included male and female mice, and sex-stratified analyses were performed where indicated. Human colectomy-derived myenteric ganglia networks included donors of both sexes; sex-stratified analyses were not performed for human tissues.

### Animals and diets.

Male and female C57BL/6J mice (6 weeks old; The Jackson Laboratory) were randomized to receive either a CD (TD.140305, 16.9% kcal from fat) or a WD (TD.140304 34.5% kcal from fat) for 12 weeks (Teklad Diets, Envigo). Body weight was monitored weekly. At week 2, mice received a single retro-orbital i.v. injection of the AAV-MaCPNS2 capsid packaged with either pAAV-CMV-EGFP or pAAV-CMV-Nfe2l2-P2A/EGFP using a 3-plasmid transfection system (VectorBuilder), delivering either 5 × 10¹¹ or 1 × 10^13^ viral genomes per mouse. Mice were euthanized at week 12, and distal colonic segments corresponding to the region assessed by the bead assay were collected for downstream molecular and histological analyses.

### Colonic transit measurement.

Distal colonic transit was assessed at week 11 using the bead expulsion test (34) briefly described in Supplemental Methods.

### Primary enteric neuronal cell isolation and culture.

Myenteric neurons were isolated from the intestines of male and female 8- to 12-week-old C57BL/6J mice, following previously established protocols (20). Cells were plated on Matrigel-coated chamber slides or 6-well plates and treated for 24 hours with vehicle (BSA), Fer-1 (10 μM), PA (0.5 mM), or PA plus Fer-1 (PA+Fer-1) (see Supplemental Methods for details).

### Culture and treatment of the murine enteric neuronal cell line.

IM-FEN cells (57) were maintained and differentiated under established conditions and then treated for 24 hours with vehicle (BSA), Fer-1 (10 μM), PA (0.5 mM), or PA+Fer-1 (see Supplemental Methods for details).

### Ca^2+^ imaging.

Ca^2+^imaging on EFS was done in the IM-FEN enteric neuronal cell line. The suitability of responses was confirmed in Wnt-1^Cre2^:GCaMP5g-tdT Ca^2+^ reporter mice (58). Fluo-4/AM loading and imaging was described previously (59). Cells or longitudinal muscle–myenteric plexus preparation (LMMP) preparations were perfused with oxygenated Krebs solution containing vehicle, PA (0.01–0.5 mM), Fer-1, or TTX, and time-series fluorescence was recorded during EFS across a range of frequencies (see Supplemental Methods for details).

### Immunofluorescence staining and imaging of myenteric neurons.

Primary enteric neuronal cells were fixed for 20 minutes at room temperature (RT) in 4% paraformaldehyde in PBS and permeabilized at 4°C for 15 minutes with 0.3% Triton-X 100. The cells were then blocked with 5% BSA in PBS for 1 hour and incubated overnight at 4°C with gentle shaking. A list of primary antibodies and their working dilutions is provided in Supplemental Table 1. After overnight incubation, the cells were incubated for 1 hour at RT with a secondary antibody (Supplemental Table 2). Nuclei were labeled with DAPI (Molecular Probes). Cells were then mounted in ProLong Gold antifade mounting medium (Invitrogen, Thermo Fisher Scientific) and visualized using an Olympus IX51 microscope (Olympus) equipped with cellSens Standard 1.12 imaging software for fluorescence imaging or a Nikon A1R equipped with NIS Elements software for confocal imaging. At least 5 fields were examined per group, and all experiments were replicated at least 3 independent times.

### FeRhoNox-1 staining.

IM-FEN enteric neuronal cells were cultured for 24 hours with PA (PA, 0.5 mM) or vehicle, with or without Fer-1. To assess labile Fe^2+^ levels, cells were incubated with 5 μM FeRhoNox-1 (Goryo Chemical) for 30 minutes at 39°C in the dark. Following incubation, cells were washed with PBS to remove unbound FeRhoNox-1. Fluorescence was captured using a Cytation C10 imaging system (Agilent Technologies), and intensity was quantified using ImageJ (NIH) to determine labile Fe^2+^ levels.

### PI staining.

Cell viability was evaluated using the ReadyProbes Cell Viability Imaging Kit (Blue/Red, catalog R37108, Thermo Fisher Scientific) following 24-hour treatments with PA (0.5 mM; MilliporeSigma), vehicle (10% BSA; MilliporeSigma), and Fer-1 (10 μM) or a combination of PA+Fer-1 (see Supplemental Methods for details).

### IHC staining and imaging of myenteric ganglia.

Paraffin-embedded intestinal sections (10 μm) were deparaffinized, rehydrated, and subjected to heat-mediated antigen retrieval. Sections were blocked in 5% BSA and incubated overnight at 4°C with primary antibodies (Supplemental Table 1), followed by fluorophore-conjugated secondary antibodies for 1 hour at RT (Supplemental Table 2). Nuclei were counterstained with DAPI, and slides were mounted with antifade medium (see Supplemental Methods for details). Fluorescence images were acquired using a Nikon A1R confocal microscope (Nikon Instruments) with NIS Elements software. From each mouse, 6–10 randomly selected myenteric ganglia were imaged for quantification. Within each experiment, control and treated sections were imaged in the same session using identical confocal acquisition settings (laser lines, laser power, detector gain, offset, pinhole, scan speed, frame size, and zoom). Image analysis was performed using ImageJ software (NIH). The neuronal area, demarcated by TUBB3 immunoreactivity, was defined as the region of interest (ROI), and fluorescence intensity of the target protein was quantified within this ROI. For each marker, a single intensity threshold and analysis pipeline were defined a priori and applied uniformly to all images from that experiment. Data were normalized to the MFI of the vehicle-treated control group and expressed as fold change in relative neuronal gene expression.

### Human tissue collection and nhMPG isolation.

The human tissue collection and nhMPG isolation procedure and timeline are illustrated in Supplemental Figure 5A, and networks of myenteric ganglia are shown in Supplemental Figure 5B. The isolation technique was revised from Grundmann et al. (60), and nhMPG networks were shown to contain viable neurons and glia in electrophysiological recordings in our laboratory (61) (see Supplemental Methods for details).

### Ex vivo treatments and immunostaining of nhMPG.

nhMPG were isolated from surgical colonic specimens and kept in DMEM/F12 medium for the duration of the study. To assess ferroptotic stress and neuronal injury, isolated nhMPG tissues were treated ex vivo for 24 hours at 37°C in a humidified 5% CO_2_ incubator with either PA 0.25 mM, 0.5 mM; MilliporeSigma, ferric ammonium citrate (FAC) (100 μM; MilliporeSigma), or LPS (1 μg/mL; MilliporeSigma). Vehicle-treated controls received DMEM or 10% BSA in DMEM.

### qRT-PCR and bulk RNA-seq.

Total RNA was isolated and reverse transcribed for TaqMan-based qRT-PCR. Relative gene expression was calculated by the 2^–ΔΔCt^ method with normalization to 18S rRNA or HPRT1 (the assay probe IDs are listed in Supplemental Table 3). For bulk RNA-seq, IM-FEN cells were treated with vehicle or PA (0.5 mM) for 24 hours (*n* = 6 per group) and processed for transcriptomic profiling by Novogene (see Supplemental Methods for details).

### Western blotting.

Enteric neuronal lysates were analyzed by SDS-PAGE and immunoblotting using standard procedures. Membranes were probed for FTH1 with β-actin as a loading control, and band intensities were quantified by densitometry (see Supplemental Methods for details).

### Measurement of mitochondrial integrity using MitoBrilliant 646.

To assess mitochondrial membrane integrity, primary enteric neuronal cells were treated with vehicle, Fer-1, or PA (0.5 mM), or a combination of PA+Fer-1 for 24 hours at 37°C in a 4-well chamber. After treatment, cells were incubated with 100 nM MitoBrilliant 646 (catalog 7700, Bio-Techne) for 1 hour at 37°C in the dark. Cells were then fixed with ice-cold 4% PFA for 20 minutes and washed with PBS. Following fixation, cells were immunostained with the neuronal marker TUBB3 (catalog TUJ-0020, Aves Labs) and counterstained with DAPI (Molecular Probes). After final PBS washes, cells were mounted, and slides were imaged using an Olympus IX51 microscope (Olympus).

### Mitochondrial iron loading.

Mitochondrial iron loading and mitochondrial mass were assessed in IM-FEN neurons using MitoFerroGreen (Dojindo, M489), together with MitoTracker Red CMXRos (Thermo Fisher Scientific, M7512). Differentiated IM-FEN cells were treated with vehicle or PA, with or without Fer-1, washed with prewarmed HBSS, and incubated with 5 μM MitoFerroGreen and 100 nM MitoTracker Red CMXRos in HBSS for 30 minutes at 37°C in the dark. Cells were then washed, maintained in fresh HBSS, and imaged live by confocal microscopy using appropriate excitation and emission settings for each dye. Regions of interest were drawn around neuronal cell bodies, background was subtracted, and mean fluorescence intensities were quantified using ImageJ/Fiji. Mitochondrial iron loading was expressed as MitoFerroGreen intensity normalized to MitoTracker Red CMXRos signal or to vehicle controls.

### Volumetric analysis of confocal Z-stacks from human myenteric ganglia.

Volumetric analysis of *Z*-stacks obtained from human networks of myenteric ganglia (nhMPG) was conducted to quantify neuronal viability, ferroptosis marker expression, and nuclear translocation. Confocal images were acquired using a Nikon A1R confocal microscope, with *Z*-stacks spanning approximately 18 μm in depth at 0.5 μm intervals (see Supplemental Methods for details).

### Statistics.

All data are presented as the mean ± SEM. Statistical analyses were performed using GraphPad Prism, version 10.0 (GraphPad Software). Depending on the experimental design, comparisons between 2 groups were conducted using unpaired, 2-tailed *t* tests. Experiments involving more than 2 groups or more than 1factor (e.g., diet, sex, AAV treatment, or PA/Fer-1) were analyzed using 1- or 2-way ANOVA models that included the appropriate interaction term. When ANOVA indicated a significant mean or interaction effect, Tukey’s post hoc test was used to control type 1 errors across multiple comparisons. A *P* value of less than 0.05 was considered statistically significant.

### Study approval.

All animal procedures were reviewed and approved by the IACUC of the Atlanta VA Health Care System (Decatur, Georgia, USA) and were conducted in accordance with Animal Research: Reporting of In Vivo Experiments (ARRIVE) and NIH guidelines. Human colonic tissues were obtained from patients undergoing elective colectomy under protocols approved by The Ohio State University IRB (Columbus, Ohio, USA) (OSU-IRB nos. 2020H0273 and 2024H0125). Written informed consent was obtained from all patients prior to tissue collection.

### Data availability.

The RNA-seq dataset generated and analyzed in this study has been deposited in the NCBI Gene Expression Omnibus (GEO) database (GEO GSE298679). Values for all data points in graphs are reported in the Supporting Data Values file. All other data supporting the findings of this study are available within the article and its supplemental material or from the corresponding author upon reasonable request.

## Author contributions

AB conceived the project, designed and performed the in vitro and in vivo animal experiments, analyzed data, and wrote the manuscript. DP and FLC performed the human nhMPG arm of the study, including tissue procurement, ex vivo treatments, imaging, and quantitative analysis. YD assisted with human nhMPG isolation, immunofluorescence, and data acquisition. JR and AH provided surgical samples and clinical metadata. YL, FC, and SMM assisted with the design and execution of mouse experiments and molecular assays, and SMM edited the manuscript. PH and CMH contributed expertise in ferroptosis mechanisms and data interpretation. WH and XY provided guidance on AAV construct design and AAV-based gene delivery experiments. JMP assisted with confocal imaging and image analysis. FLC supervised human studies. FLC and DP contributed to data interpretation and manuscript submission. SS supervised the overall study, secured funding, contributed to the experimental designs, and edited the manuscript. All authors reviewed and approved the final version of the manuscript. AB and DP contributed equally to this work. Co–first authorship order was determined by mutual agreement among the co–first authors in consultation with the senior authors, and was based on overall contributions to study conception, experimentation, analysis, and manuscript preparation.

## Conflict of interest

The authors have declared that no conflict of interest exists.

## Funding support

This work is the result of NIH funding, in part, and is subject to the NIH Public Access Policy. Through acceptance of this federal funding, the NIH has been given a right to make the work publicly available in PubMed Central.

- NIH grant R01 DK080684 (to SS).
- NIH grant RO1 DK125809 (to FLC).
- VA Merit Award 2I01BX000136 (to SS).
- Mini-sabbatical award from the American Neurogastroenterology and Motility Society (ANMS) (to AB) to support training in the laboratory of Fievos L. Christofi at The Ohio State University, focused on the isolation and imaging of human networks of myenteric ganglia.

## Supplementary Material

Supplemental data

Unedited blot and gel images

Supporting data values

## Version 1 04/21/2026

In-Press Preview

## Version 2 06/01/2026

Electronic publication
