# Supplementary material for: Western diet induces iron-dependent enteric neurodegeneration via ferroptosis
Source: J Clin Invest. 2026 Apr 21;136(11):e196113. doi: 10.1172/JCI196113 (PMC13221235; doi:10.1172/JCI196113)
Supplement: Supplemental data [file jci-136-196113-s206.pdf]

Supplemental Information

Western Diet Induces Iron-Dependent Enteric Neurodegeneration via Ferroptosis: Mechanistic Insights from Murine and Human Models

Arun Balasubramaniam[1,2†], Dmitrii Pavlov[4†], Yunpeng Du[4], Jeremy Reeves[4], Alan Harzman[5], Yunshan Liu[1,2], Francesca Cingolani[1,2], Xinxu Yuan[6], Jay M. Patel[2,7], Simon Musyoka Mwangi[1,2], Peijian He[1], C. Michael Hart[2,3], Wenhui Hu[6], Fievos Christofi[4], Shanthi Srinivasan[1,2*]

[1]Division of Digestive Diseases, Emory University School of Medicine, Atlanta, GA, USA. [2]Atlanta VA Health Care System, Atlanta, GA, USA.

[3]Division of Pulmonary, Allergy, Critical Care and Sleep Medicine, Emory University School of Medicine, Atlanta, GA, USA.

[4]Department of Anesthesiology, The Ohio State University, Columbus, OH, USA.

[5]Department of Surgery, The Ohio State University, Columbus, OH, USA.

[6]Department of Neuroscience and Anatomy, Virginia Commonwealth University, Richmond, VA, USA.

[7]Department of Orthopaedics, Emory University School of Medicine, Atlanta, GA, USA

Running title: Ferroptosis in the ENS

*Corresponding author email address: ssrini2@emory.edu (S.S)

†: Co-first authors contributed equally to this work

Materials and Methods

Colonic Transit Measurement

A 3 mm glass bead was gently inserted 2 cm into the distal colon under brief isoflurane anesthesia with lubricant applied to minimize pain. Mice were placed individually in clean cages, and the time from bead insertion to bead expulsion was recorded.

Primary Enteric Neuronal Cell Isolation and Culture

The isolated cells were suspended in Neurobasal A Medium (Cat. #10-888-022, Gibco, Grand Island, NY, USA) enriched with 2 mM L-glutamine (Cat. #25-030-081, Gibco), B27 supplement (Cat. #A3582801, Gibco), penicillin/streptomycin (Cat. #15-140-122, Gibco), 10 ng/mL glial cell line-derived neurotrophic factor (GDNF; Cat. #200-37, Shenandoah Biotechnology, Warwick, PA, USA), and 1% fetal bovine serum (FBS; Cat. #S10650H, Atlanta Biologicals, Atlanta, GA, USA). Cells were plated into Matrigel-coated 4-well chamber slides at a density of 1×10^4 cells per well and 6-well plates at 1×10^5 cells per well. After five days of incubation, once the cultures reached confluency, the medium was replaced, and treatments were initiated. Enteric neuronal cells were exposed to vehicle (10% BSA), Fer-1 (Fer-1, 10 µM, Cat. #SML0583, Sigma-Aldrich, St. Louis, MO, USA), palmitic acid (PA, 0.5 mM; Cat. #P5585, Sigma-Aldrich), or a combined treatment of PA+Fer-1 for 24 h.

Culture and Treatment of Mouse Enteric Neuronal Cell Line

IM-FEN were seeded in modified N2 medium supplemented with glial cell line-derived neurotrophic factor (GDNF; Shenandoah Biotechnology, Warwick, PA, USA), 10% fetal bovine serum (FBS; Atlanta Biologicals, Flowery Branch, GA, USA), and 20 U/mL recombinant mouse interferon-γ (Chemicon, Cat. #IF005, Temecula, CA, USA). Cells were cultured at 33°C in a humidified incubator with 5% CO_2. After 24 h, the culture medium was replaced with Neurobasal-A medium (Gibco) supplemented with B-27 Plus Supplement (Gibco), 1 mM L-glutamine (Gibco), 1% FBS (Atlanta Biologicals), and GDNF (Shenandoah Biotechnology). Cells were then transferred to 39°C to promote differentiation. After reaching confluency, treatments were performed for 24 h using Fer-1 (Fer-1, 10 µM; Sigma-Aldrich), palmitic acid (PA, 0.5 mM; Sigma-Aldrich), a combination of PA and Fer-1 (PA+Fer-1), or vehicle control (10% bovine serum albumin, BSA; Sigma-Aldrich).

Palmitic acid Preparation

Palmitic acid (PA; Sigma, P5585, cell culture grade) was prepared by dissolving PA in 200 proof ethanol at 37°C to generate a 250 mM stock solution, which was then added dropwise to a 10 percent (wt/vol) fatty acid free BSA solution (Sigma, A8806) prepared in sterile deionized water at 37°C with constant mixing to yield a 6 mM palmitate BSA stock. This stock was filter sterilized, aliquoted, protected from light, stored at −20°C, and diluted into prewarmed culture medium.

Ca^{2+} imaging

IM-FEN were plated at 2 × 10^5 cells per 30 mm dish on number 0 coverslips and grown to confluency, incubated with 2 μM Fluo-4 AM (Invitrogen, Eugene, OR) for 30 min at 37°C in 5%CO_2 followed by 30 min de-esterification. Ca2+ imaging was carried out on an upright Nikon Eclipse FN1 microscope with a 20× water-immersion objective using a standard Fluo-4/GCaMP filter set. LMMP (0.8×0.8cm²) was pinned over a Sylgard-embedded glass support for visualization/imaging. Time-series images of Fluo-4 or GCaMP5g fluorescence were acquired at 7 frames/s using an ANDOR iXon Ultra 897 EMCCD camera (Andor, Belfast, UK) controlled by NIS Elements software (Nikon). Preparations were perfused at 4 ml/min with oxygenated Krebs solution containing vehicle, BSA (10%), PA (0.01, 0.1, 0.5 mM), Fer-1 (10 μM), or TTX (1 μM). Acute PA exposure was monitored for 10-12 min; TTX was preincubated for 10 min, and Fer-1 for 24 h before imaging. Perfusate temperature was maintained at 36.5±0.5°C using an in-line heater (Warner Instruments, Hamden, CT). EFS was delivered via a Grass S88 stimulator (Grass Medical Instruments, Quincy, MA) using 0.1ms pulses at 40 V for 5 s at 0.5-25 Hz with 3 min inter- stimulus intervals. To reduce muscle contractions in LMMP, tissues were treated with atropine (10μM) and nicardipine (3μM). EFS was applied using a field-stimulation chamber (RC-49MFSH, Warner Instruments).

Propidium Iodide Staining

Two drops each of NucBlue Live reagent (Hoechst 33342) and propidium iodide (PI) were added directly to 1 mL of cell culture medium, and cells were incubated under standard culture conditions. After staining, excess dye was removed by washing with PBS. Confocal images were acquired using a Cytation C10 imaging system (Agilent Technologies), and the number of PI-positive cells was quantified to determine treatment-induced cell death.

Lipid peroxidation assay (C11-BODIPY 581/591)

Ferroptotic lipid peroxidation was quantified using the oxidation-sensitive probe C11-BODIPY 581/591 (Thermo Fisher Scientific, D3861). IM-FEN or primary ENS cultures were treated with vehicle or palmitic acid with or without Fer-1 (10 μM) or DFO (50 μM), or with DFO alone, for the indicated times. Cells were washed once with warm HBSS or phenol red free medium and incubated with 1 μM C11-BODIPY diluted in HBSS for 20-30 min at 37°C in the dark. After dye loading, cells were washed, maintained in fresh HBSS, and imaged live by confocal microscopy with sequential acquisition of the reduced (red, ~590 nm) and oxidized (green, ~510 nm) emission channels. Lipid peroxidation was expressed as the ratio of oxidized to reduced C11-BODIPY signal (green/red) or normalized to vehicle controls, providing a quantitative index of PA-induced lipid oxidation and its inhibition by Fer-1 or DFO.

Cell viability assay (CellTiter-Glo ATP)

Cellular ATP content was used as a surrogate for viability using the CellTiter-Glo Luminescent Cell Viability Assay (Promega) according to the manufacturer's instructions. IM-FEN cells were plated in white, opaque 96-well plates at 5000 cells/well and allowed to adhere and/or differentiate as described above, then treated with vehicle or palmitic acid (0.01-4mM) with or without Ferrostatin-1 for the indicated durations. At the end of 24h treatment, plates were equilibrated to RT for 10 min, an equal volume of CellTiter-Glo reagent was added directly to each well, and contents were mixed on an orbital shaker for 2 minutes to induce cell lysis. After a 10-

min incubation at RT to stabilize the luminescent signal, ATP-dependent luminescence was measured on a plate reader. Background signal from cell-free wells was subtracted, and values were normalized to vehicle-treated controls from the same plate to yield relative ATP levels.

Trypan Blue cell viability assay

Trypan Blue (0.4% Gibco, Thermo Fisher Scientific; Cat. No. 15250061) was added directly to the neuronal culture medium at a final concentration of 0.1%. Cultures were incubated for 3 min at room temperature, gently rinsed with warm medium to remove excess dye, and immediately imaged. Quantification was performed by counting dye-positive (non-viable) and dye-negative (viable) cells within fields of view.

Assay for Mitochondrial Reactive Oxygen Species (ROS)

IM-FEN cells were seeded into 6-well culture plates and grown to approximately 80% confluency and were then treated with vehicle control (BSA 10%), Ferrostatin-1 (Fer-1; 10 μM; Sigma-Aldrich), palmitic acid (PA; 0.5 mM; Sigma-Aldrich), or a combination of PA and Fer-1 (PA+Fer-1) for 24 h. Following treatment, cells were incubated with 5 μM MitoSOX™ Red mitochondrial superoxide indicator (Cat. #M36008, ThermoFisher Scientific, Eugene, OR, USA) for 10 minutes in the dark at 39°C. After incubation, cells were washed with PBS and counterstained with DAPI (Molecular Probes) for 5 minutes. Cells were then washed twice with PBS and imaged using Cytation C10 imaging system (Agilent Technologies).

Immunohistochemistry (IHC) Staining and Imaging of Myenteric Ganglia

Paraffin-embedded intestinal tissue samples were sectioned at a thickness of 10 μm using a microtome and mounted onto glass microscope slides. Sections were deparaffinized in xylene and rehydrated through a descending ethanol gradient. Antigen retrieval was performed by heating the slides in antigen retrieval buffer (Cat. #ab93678, Abcam, Waltham, MA, USA) at 95 °C for 20 minutes. Following a gradual return to RT, sections were washed in phosphate-buffered saline (PBS) and blocked in 5% bovine serum albumin (BSA) prepared in PBS for 1 h at RT. Primary antibodies as indicated in Supplementary Table 1 were applied to the sections and incubated overnight at 4 °C with gentle rocking. The following day, slides were washed with PBS and incubated with appropriate fluorophore-conjugated secondary antibodies (Supplementary Table 2) for 1 h at RT. Nuclei were counterstained with DAPI (Molecular Probes), and slides were mounted using ProLong Gold Antifade Mountant (Invitrogen) before cover slipping.

Human Tissue Collection and nhMPG Isolation

Resected colon specimens were freshly dissected in cold oxygenated Krebs solution pinned to sylgard in a 200 mm culture dish. Following removal of serosal fat, the mucosa and submucosa were carefully micro-dissected away to expose the muscularis externa (ME). The circular and longitudinal muscle layers were trimmed gradually and carefully from both sides, to remove as much of the thicker circular muscle as possible without disturbing the networks of myenteric ganglia using Vannas scissors. The ME was then cut into ~0.2 × 0.5 cm² pieces and placed in 35 mm Petri dishes. Tissues were washed three times for 15 minutes each with sterile HBSS. Enzymatic digestion was performed using freshly prepared Liberase solution containing Liberase TH Research Grade (0.75 mg/mL: Cat. #05401151001, Roche, Indianapolis, IN, USA),

DNase I (0.1 mg/mL; Sigma-Aldrich, St. Louis, MO, USA), Amphotericin B (250 µg/mL; Cat. #2571510, Gibco, Grand Island, NY, USA), penicillin/streptomycin (Gibco), and DMEM/F12 (Cat. #2997898, Gibco). The solution was filtered through a 0.22 µm Millipore Express PES membrane (Cat. #SLGPR33RS, Merck Millipore, Burlington, MA, USA). Digestion was carried out at 37°C with 5% CO_2 for 18 h. Following digestion, tissue was gently triturated and rocked at 30 rpm for 1 h on a Corning LSE XL platform rocker (Corning Inc., Corning, NY, USA). Ganglia networks were collected using an Eppendorf pipettor and diluted in DMEM/F12 to reduce tissue density. Ganglia were visualized and harvested using a Zeiss Telaval 31 inverted microscope (Zeiss, Oberkochen, Germany) at 100× magnification.

Ex Vivo Treatments and Immunostaining of nhMPG

Following PA or PA+Fer-1 treatment, cell death was evaluated using the ReadyProbes Cell Viability Imaging Kit (Cat. #R37108, Invitrogen, Eugene, OR, USA), where live nhMPG were incubated with 70 µL of propidium iodide (PI) solution for 30 minutes and then washed four times with ice-cold PBS (pH 7.4). The tissue was fixed with 4% paraformaldehyde for 10 minutes and washed again. For immunofluorescence, tissues were blocked with 10% normal donkey serum (Jackson ImmunoResearch, West Grove, PA, USA) for 1 h and incubated with primary antibodies overnight at 4°C. The following day, tissues were washed and incubated with secondary antibodies and DAPI (Cat. #R37606, Invitrogen) for 2 h at RT. Samples were mounted and cover slipped with 22 × 22 mm glass coverslips (Cat. #1404-10, Globe Scientific, Mahwah, NJ, USA). Co-labeling of neuronal (HuC/D) and ferroptosis markers (FTH-1, TfR1) was performed in two-well chamber slides (Cat. #154461, ThermoScientific). Confocal imaging was conducted using a Nikon A1R confocal microscope (Nikon Instruments, Melville, NY, USA) equipped with a 40× Plan-Fluor oil DICH N2 objective (NA 1.3, WD 240 µm), and z-stack images (~18 µm total depth at 0.5 µm intervals) were acquired. A full list of antibodies used is provided in Supplementary Tables 1 and 2.

Quantitative Real-Time PCR (qRT-PCR) and Bulk RNA Sequencing

Total RNA was isolated using the illustra RNAspin Mini Kit (Cat. #25-0500-71, Sigma-Aldrich, St. Louis, MO, USA) following the manufacturer's protocol. Complementary DNA (cDNA) was synthesized from purified RNA using the SuperScript IV VILO Master Mix (Cat. #11756050, ThermoFisher Scientific, Waltham, MA, USA). Quantitative PCR reactions were prepared using TaqMan Gene Expression Master Mix (Cat. #4369016, ThermoFisher Scientific) and TaqMan` Gene Expression Assays (ThermoFisher Scientific) targeting specific mouse genes. PCR amplification and fluorescence detection were performed using the StepOnePlus Real-Time PCR System (Applied Biosystems, Foster City, CA, USA). The relative expression of neuronal genes, including nNOS, TUBB3, and Nfe2l2, was normalized to housekeeping genes 18S rRNA or HPRT1 using the $2^{-\Delta\Delta Ct}$ method. A full list of TaqMan assay probes used are provided in Supplementary Tables 3.

IM-FEN cells were cultured under standard conditions and treated with either vehicle (Veh) or 0.5 mM palmitic acid (PA; Sigma-Aldrich,) for 24 h. Total RNA was isolated, and a total of 12 RNA samples (n = 6 per group) were submitted to Novogene Corporation Inc. (Sacramento, CA, USA) for transcriptomic profiling using their Plant and Animal Eukaryotic mRNA-seq with Reference (WBI-Quantification) pipeline.

Western Blotting

Enteric neuronal cell lysates were prepared using 4× Laemmli Sample Buffer (Cat. #1610747, Bio-Rad, Hercules, CA, USA) supplemented with Complete Mini Protease Inhibitor Cocktail Tablets (Cat. #04693116001, Roche Diagnostics, Mannheim, Germany). Protein samples, along with Precision Plus Protein Dual Color Standards (Cat. #1610374, Bio-Rad), were separated by SDS-PAGE using 4-20% Criterion TGX Precast Midi Protein Gels (Cat. #5671093, Bio-Rad) following the manufacturer's instructions. Proteins were then transferred to Immuno-Blot PVDF membranes (Cat. #1620177, Bio-Rad) using standard wet transfer conditions. Membranes were blocked and then incubated overnight at 4°C with primary rabbit antibodies against FTH-1 (1:1000; Abcam, Cambridge, MA, USA) and mouse anti-β-actin (1:5000; Cell Signaling Technology, Danvers, MA, USA). Following PBS-T washes, membranes were treated with horseradish peroxidase (HRP)-linked secondary antibodies targeting either rabbit or mouse IgG (Cell Signaling Technology) at a 1:5000 dilution. Bands were visualized using chemiluminescence detection, and semi-quantitative band intensity analysis was performed using ImageJ software (National Institutes of Health, Bethesda, MD, USA).

Volumetric Analysis of Confocal Z-Stacks from Human Myenteric Ganglia

Within each experiment, all treatment groups were imaged using identical confocal acquisition parameters, and the same segmentation and thresholding settings were applied across all z-stacks in that dataset (i.e. laser lines, laser power, detector gain, offset, pinhole, scan speed, frame size, and zoom). Image quantification was performed using NIS Elements software (version AR 5.42.05, Nikon Instruments, Melville, NY, USA). Each treatment condition was tested using tissue from a minimum of three colectomy patients, and 12 individual z-stacks each representing an isolated ganglionic network. Therefore, for each treatment 36 z-stacks were analyzed. Analysis included total number and area of PI^+ and HuC/D^+ cells per field, co-localization area of PI and HuC/D, area of $DAPI^+$ nuclei, density and pixel intensity of HuC/D^+ neurons, and extent of nuclear translocation based on HuC/D-DAPI overlap. Expression of ferroptosis markers, including FTH1 and TfR1, was assessed specifically in HuC/D^+ neurons, with quantification based on number of co-labeled cells, signal intensity, and co-localized area.

Supplementary Figures

Supplementary Figure 1. Palmitic acid modulates genes involved in calcium signaling and neurotransmission in enteric neurons. (A-D) Heatmaps showing transcriptional changes in immortalized enteric neuronal cells (IM-FEN) treated with vehicle (Veh) or palmitic acid (PA, 0.5 mM) for 24 hours (n = 6 per group). Bulk RNA-seq was performed to assess global gene expression changes. (A) Calcium signaling-related genes including ion channels, calcium pumps, and calcium-binding proteins were significantly altered by PA, indicating disrupted intracellular calcium homeostasis. (B) Synaptic transmission-related genes including components of synaptic vesicles and exocytosis machinery were broadly downregulated following PA exposure, suggesting impaired synaptic function. (C) Genes involved in excitatory neurotransmission were reduced in PA-treated cells, consistent with suppression of excitatory signaling pathways. (D) Genes associated with inhibitory neurotransmission showed partial decreased expression, suggesting a broader disruption in the excitatory/inhibitory neuronal balance. Heatmap values represent $\log_2$-transformed, z-score normalized gene expression. Columns represent individual biological replicates, clustered to illustrate treatment-specific transcriptional signatures.

Supplementary Figure 2: Palmitic acid induces heat shock protein expression in enteric neurons and western diet increases body weight in mice. (A) Heatmap showing differential

expression of heat shock proteins (HSPs) in IM-FEN cells treated with vehicle (Veh) or palmitate (PA, 0.5 mM) for 24 hours (n = 6 per group). Genes include molecular chaperones and protein-folding regulators involved in cellular stress responses. Values are $\log_2$-transformed, z-score normalized counts. Hierarchical clustering reveals distinct separation between Veh- and PA-treated cells, with robust upregulation of HSPs following PA exposure. (B) Heatmap of lipid metabolism and obesity-related gene expression in IM-FEN cells treated with vehicle (Veh) or palmitic acid (PA, 0.5 mM) for 24 h. (C) Heatmap of inflammatory signaling and neurotransmission-associated motility regulators in the same conditions. (D-E) Body weight measurements of male (D) and female (E) mice over a 12-week period. Mice were fed control diet (CD) or Western diet (WD) and received AAV-eGFP or AAV-Nfe2l2 at week 2. WD-fed animals of both sexes gained significantly more weight over time compared to RD-fed controls, irrespective of AAV treatment. Data are presented as mean ± SEM; n = 4-6 mice per group.

Supplementary Figure 3. AAV-mediated overexpression of Nfe2l2 enhances neuronal Nfe2l2 expression in the colon. A) Quantitative RT-PCR analysis showing increased colonic Nfe2l2 mRNA expression in AAV-Nfe2l2-treated mice compared to AAV-eGFP controls. Data represent technical triplicates from male and female samples and confirm successful AAV overexpression. Data represent mean ± SEM. Statistical analysis was performed using t-test. *P< 0.05; ***P < 0.001; B) Representative whole-mount confocal images of the myenteric plexus displaying eGFP fluorescence in mice treated with AAV-eGFP (Control) or AAV-Nfe2l2. Images confirm effective viral transduction across the enteric neuronal network. Scale bar, 50 µm.

Supplementary Figure 4. AAV-Nfe2l2 preserves neuronal nitric oxide synthase (nNOS) expression in the colonic myenteric plexus of Western diet-fed mice. Representative immunofluorescence images of colon tissue sections from male and female mice fed control diet (CD) or Western diet (WD) for 12 weeks and treated with AAV-eGFP or AAV-Nfe2l2. Sections were co-stained for the pan-neuronal marker TUBB3 (cyan) and neuronal nitric oxide synthase (nNOS, red). Merged images highlight co-localization of nNOS within TUBB3+ neurons. AAV-Nfe2l2 administration preserved nNOS expression in WD-fed mice compared to AAV-eGFP controls. Quantification of the proportion of nNOS+ neurons among total TUBB3+ neurons across groups. A total of 28 mice were used: n = 4 mice per group for CD and WD AAV-eGFP, and n = 3 mice per group for CD and WD AAV-Nfe2l2. From each mouse, 6-10 randomly selected myenteric ganglia were imaged and analyzed. Data represent mean ± SEM. Statistical analysis was performed using two-way ANOVA. *P < 0.05; **P < 0.01; ***P < 0.001; ns = not significant. Scale bars, 50 µm.

Supplementary Figure 5. Logistics and timeline for procurement of human colon surgical specimens, isolation of human networks of ganglia and in vitro experiments with palmitic acid. (A) Schematic showing the timeline from patient consent to isolation of nhMPG networks and palmitic acid induction experiments. (B) Examples of nhMPG networks obtained after colectomy in human patients are shown with transmitted light imaging.

Supplementary Figure 6. Differential Induction of Neuronal and Non-Neuronal TfR1 Expression by PA, FAC, and LPS in Human nhMPG Networks. (A-E) Representative confocal images of human networks of myenteric ganglia (nhMPG) treated with ferric ammonium citrate (FAC, 100 µM) or lipopolysaccharide (LPS, 1 µg/mL) for 24 h. Sections were stained for DAPI (blue), the pan-neuronal marker HuC/D (red), and transferrin receptor 1 (TfR1, green). Merged images show co-localization of neuronal TfR1 (yellow). Representative confocal images of FAC-treatment, shown as z-stack projections from different fields of view, increased neuronal TfR1 expression (A-D), while LPS also induced TfR1 expression in enteric neurons (E). (F-G) Additional

examples of LPS-treated ganglia showing broad upregulation of TfR1 in both neuronal and non-neuronal regions. (H-K) Quantification of TfR1 expression across conditions. (H) Neuronal TfR1 expression/field was significantly increased by PA (0.5 mM) and LPS (1 µg/mL) to a similar extent, while FAC induced a markedly greater increase. (I) FAC induced neuronal TfR1 expression in a significantly larger number of HuC/D^{+} neurons per field compared to PA. (J) In contrast, non-neuronal TfR1 expression was significantly greater with PA than with FAC, based on both total area and number of distinct TfR1-positive regions per field. (K) Pixel intensity analysis revealed that FAC resulted in marginally but significantly higher TfR1 intensity than PA. All data were analyzed by one-way ANOVA followed by Tukey's post hoc test. Values represent mean ± SEM. *P < 0.05, **P < 0.01, ***P < 0.001. Quantification of TfR1 expression was performed using Nikon NIS-Elements co-localization module from 18 µm-thick z-stacks acquired at 0.5 µm intervals.

Supplementary Figure 7. Palmitic acid disrupts ganglionic morphology and induces glial activation in human nhMPG networks. (A-B) Representative confocal images showing structural abnormalities in human myenteric ganglia (nhMPG) following PA (0.5 mM, 24 h) treatment. Distortion or fragmentation of the ganglionic network was observed in a subset of patient samples. (C) PA treatment induced glial fibrillary acidic protein (GFAP) expression in HuC/D glial cells within the nhMPG, consistent with reactive gliosis. GFAP is not typically detected in healthy human enteric glia. Quantification was performed in ganglionic networks from a representative patient (n = 9 networks per condition). Values are expressed as mean ± SEM. *P < 0.05, **P < 0.01, ***P < 0.001.

Supplementary Figure 8. Palmitate induces lipid peroxidation and ferroptosis-dependent loss of nNOS, ChAT, and TH enteric neurons. (A) Cell viability of IM-FEN enteric neurons treated for 24 h with increasing concentrations of palmitate (PA, 0.01-4 mM) measured by CellTiter-Glo. 0.5 mM PA (red bar) was chosen for subsequent experiments as it reduced viability without causing overt cell loss. (B) (B) C11-BODIPY fluorescence in IM-FEN cells treated for 24 h with vehicle (Veh), PA (0.5 mM), ferrostatin-1 (Fer-1, 10 µM), PA + Fer-1, deferoxamine (DFO, 50 µM), or PA + DFO. PA increases lipid peroxidation (oxidized C11-BODIPY signal), which is attenuated by Fer-1 and DFO. Histogram shows normalized oxidized/reduced C11-BODIPY fluorescence relative to Veh. (C) Confocal images of primary enteric neuron networks treated with Veh, PA, Fer-1, or PA + Fer-1 for 24 h and stained for TUBB3 (green), nNOS (red), ChAT (cyan), and TH (magenta). Quantification of nNOS-, ChAT-, and TH-expressing neurons demonstrates loss of all three neuronal populations with PA, partially restored by Fer-1. Scale bars: 50 µm. Data represent mean ± SEM from three independent experiments; Values are expressed as mean ± SEM. *P < 0.05, **P < 0.01, ***P < 0.001.

Supplementary Figure 9. Time dependent induction of ferroptosis genes, loss of viability, and mitochondrial ROS during palmitate exposure in enteric neurons. (A-D) qRT-PCR analysis of ferroptosis-related genes transferrin receptor 1 (Tfr1), Il6, Gpx4, and Aifm2 in IM-FEN enteric neurons treated with vehicle (Veh) or palmitate (PA, 0.5 mM) for 0, 4, 12, and 24 h. Data are expressed as fold change in mRNA relative to time-matched Veh. (E) Trypan Blue cell viability assay of IM-FEN enteric neurons showing that acute PA exposure does not significantly reduce viability, whereas chronic PA markedly decreases viable neurons, an effect prevented by chronic Fer-1 co-treatment. (F) Representative images of MitoSOX Red (mitochondrial superoxide) and DAPI in IM-FEN neurons treated with Veh or PA for 24 h, with quantification of MitoSOX fluorescence intensity normalized to Veh, demonstrating increased mitochondrial ROS after PA exposure. Scale bars: 50 µm. Data represent mean ± SEM from three independent experiments; Values are expressed as mean ± SEM. *P < 0.05, **P < 0.01, ***P < 0.001; ns, not significant.

Supplementary Figure 10. Western diet increases Drp1 and 4-HNE in TH-positive myenteric neurons *in vivo*. Representative confocal images of distal colon myenteric plexus from mice fed control diet (CD) or Western diet (WD) for 12 weeks, stained for the pan-neuronal marker TUBB3 (green), tyrosine hydroxylase (TH, cyan), the mitochondrial fission protein Drp1 (magenta), and the lipid peroxidation marker 4-HNE (red). Dashed lines outline TH-positive neuronal cell bodies and processes. Merged images show increased Drp1 and 4-HNE within TH-positive neurons in Western diet exposed mice compared with controls, mapping mitochondrial stress and lipid peroxidation in this catecholaminergic subset. Bar graphs quantify TH-positive neuron density and Drp1 and 4-HNE fluorescence intensity within TH-positive neurons. Scale bars: 20 μm. Data represent mean ± SEM from n = 4 mice per group; Data represent mean ± SEM from three independent experiments; Values are expressed as mean ± SEM. *P < 0.05, **P < 0.01, ***P < 0.001; ns, not significant.

Supplementary Figure 11. Western diet increases Drp1 and 4-HNE in nNOS-positive myenteric neurons in vivo. Representative confocal images of distal colon myenteric plexus from mice fed control diet (CD) or Western diet (WD) for 12 weeks, stained for the pan-neuronal marker TUBB3 (green), neuronal nitric oxide synthase (nNOS, red), the mitochondrial fission protein Drp1 (magenta), and the lipid peroxidation marker 4-HNE (cyan). Dashed lines outline nNOS-positive neuronal cell bodies and processes. Merged images show increased Drp1 and 4-HNE signal within nNOS-positive neurons in Western diet exposed mice compared with controls, mapping mitochondrial stress and lipid peroxidation in this nitrergic subset. Bar graphs quantify nNOS-positive neuron density and Drp1 and 4-HNE fluorescence intensity within nNOS-positive neurons. Scale bars: 20 μm. Data represent mean ± SEM from n = 4 mice per group; Data represent mean ± SEM from three independent experiments; Values are expressed as mean ± SEM. *P < 0.05, **P < 0.01, ***P < 0.001.

Supplementary Figure 12. Western diet alters Drp1 and 4-HNE in ChAT-positive myenteric neurons in a sex-dependent manner. Representative confocal images of distal colon myenteric plexus from mice fed control diet (CD) or Western diet (WD) for 12 weeks, stained for the pan-neuronal marker TUBB3 (green), choline acetyltransferase (ChAT, red), the mitochondrial fission protein Drp1 (magenta), and the lipid peroxidation marker 4-HNE (cyan). Dashed lines outline ChAT-positive neuronal cell bodies and processes. Merged images show increased 4-HNE signal within ChAT-positive neurons in Western diet exposed mice compared with controls, consistent with enhanced lipid peroxidation. Quantification demonstrates that Drp1 intensity within ChAT-positive neurons is unchanged in males and reduced in females, whereas ChAT-positive neuron density in females is not significantly altered. Scale bars: 20 μm. Data represent mean ± SEM from three independent experiments; Values are expressed as mean ± SEM. *P < 0.05, **P < 0.01, ***P < 0.001.

Supplementary Figure 13. Western diet suppresses phospho-Nfe2l2 in myenteric neurons and AAV Nfe2l2 restores antioxidant signaling. Immunofluorescence staining of distal colon sections from male and female mice fed control diet or Western diet and treated with AAV-eGFP or AAV-Nfe2l2, co-stained for TUBB3 (cyan) and phospho Nfe2l2 (P-Nfe2l2, red). Merged images show reduced neuronal P Nfe2l2 signal in Western diet AAV eGFP groups and enhanced P-Nfe2l2 expression within $TUBB3^+$ neurons in Western diet AAV Nfe2l2 treated mice. Histograms show fold change in $TUBB3^+$ neuron density and P-Nfe2l2 fluorescence intensity within $TUBB3^+$ neurons, normalized to the control diet AAV eGFP group. Scale bars: 50 μm. Data represent mean ± SEM from n = 3-4 mice per group. Statistical analysis was performed using two-way ANOVA. *P < 0.05; **P < 0.01; ***P < 0.001; ns, not significant.

Supplementary Figure 14. Concentration-dependent effect of palmitic acid on induction of neuronal cell death in nhMPG networks. Networks of human myenteric ganglia (nhMPG)

isolated from GI surgical specimens were exposed in vitro to vehicle (Veh, DMEM, 0.25mM or 0.5mM PA concentration for 24 h). PA 0.25mM consistently mirrored vehicle responses and therefore did not trigger ferroptotic injury. PA had no effect on (A) HuC/D nuclear translocation (stress response in neurons), (B) neuronal cell death, PI nuclear staining) and (C) HuC/D cell expression. (D-F) In contrast, PA 0.5mM elicited robust cell-death and stress-response signals. Statistical comparisons were performed by one-way ANOVA followed by Tukey's post hoc test. Data are presented as mean ± SEM; *P < 0.05, **P < 0.01, ***P < 0.001; ns, not significant. Quantification of cell death and HuC/D translocation was conducted using Nikon NIS-Elements colocalization tools in 18-μm confocal z-stacks acquired at 0.5-μm intervals. A total of 36 ganglionic networks per treatment group were analyzed. Human colonic tissue was obtained from 3 human subjects for each treatment, with 12 z-stacks from distinct nhMPG networks per human subject used for all colocalization and statistical analyses.

Supplementary Figure 15. Concentration-dependent increase in neuronal TfR1 activation by palmitic acid (PA) in nhMPG networks. The effect of palmitic acid was restricted to 0.5mM concentration. A lower concentration of 0.25mM had no effect on TfR1 activation. Networks of human myenteric ganglia (nhMPG) isolated from colectomy specimens were exposed ex vivo to Veh, DMEM, PA 0.25mM or PA 0.5mM. PA (0.25mM) did not increase TfR1 expression in (A) the number of HuC/D$^+$TfR1$^+$ neurons, (B) TfR1$^+$ area of colocalization with HuC/D$^+$, and (C) the pixel intensity of neuronal TfR1 immunoreactivity. In contrast, PA 0.5mM produced consistent evidence of ferroptotic signaling, with (D) significant increases in HuC/D$^+$TfR1$^+$ neuronal counts, (E) TfR1$^+$ area per neuron and (F) neuronal TfR1 fluorescence intensity. Statistical comparisons were performed using one-way ANOVA followed by Tukey's post hoc test. Data are reported as mean ± SEM; *P < 0.05, **P < 0.01, ***P < 0.001; ns, not significant. Quantification of TfR1 expression was performed using Nikon NIS-Elements colocalization tools in 18-μm confocal z-stacks collected at 0.5-μm optical intervals. A total of 36 ganglionic networks per treatment group were analyzed. Human colonic tissue was obtained from 3 GI surgical cases, with 12 z-stacks from distinct nhMPG networks per human subject included in colocalization and statistical analyses.

Supplementary Figure 16. **Concentration-dependent Ferritin (FTH-1) ferroptotic responses to palmitic acid in human myenteric ganglia, with neuronal susceptibility occurring mainly at 0.5mM PA.** Networks of human myenteric ganglia (nhMPG) isolated from colectomy specimens were treated in vitro with vehicle, DMEM, PA 0.25mM or PA 0.5mM concentration. Ferritin (FTH-1) activation in neuronal (HuC/D$^+$) and non-neuronal (HuC/D$^-$) cell populations was analyzed for complementary quantitative parameters. (A) PA 0.25mM did not increase neuronal Ferritin in nhMPG networks compared to Veh, whereas upregulation occurred in comparison to the DMEM group. (B) In contrast, PA 0.5mM triggered a ferroptotic response, with significant increases in Ferritin (FTH-1) pixel intensity in HuC/D$^+$ neurons, number of Ferritin$^+$HuC/D$^+$ neurons, and Ferritin-HuC/D colocalized area, all reflecting robust neuronal FTH-1 upregulation. (C, D) In non-neuronal cells, PA 0.25mM or PA 0.5mM both elicited ferroptotic changes in comparison to Veh across all metrics: number of Ferritin$^+$ non-neuronal cells, non-neuronal area, and pixel intensity in non-neuronal Ferritin$^+$cells. Statistical comparisons were performed by one-way ANOVA followed by Tukey's post hoc test. Values are presented as mean ± SEM; *P < 0.05,**P < 0.01, ***P < 0.001; ns, not significant. Quantification of Ferritin expression and colocalization was performed using Nikon NIS-Elements on 18-μm confocal z-stacks acquired at 0.5-μm optical intervals. Thirty- six ganglionic networks per treatment group were analyzed. Colonic tissue was collected from 3 human donors, with 12 z-stacks from distinct nhMPG networks per human subject specimen included in all analyses.

Supplementary Table 1: List of Primary antibodies

	Gene	Host	Cat no.	Conc	Company	City	State	Country
1	TUBB3	MS	AB78078	1:400	Abcam	Waltham	MA	USA
2	TUBB3	RB	AB52623	1:400	Abcam	Waltham	MA	USA
3	TUBB3	CK	TUJ-0020	1:400	Aves Labs	Davis	CA	USA
4	p-NFE2L2	RB	PA5-67520	1:200	ThermoFisher	Rockford	IL	USA
5	4-HNE	MS	MA5-27570	1:200	ThermoFisher	Rockford	IL	USA
6	MFRN2	RB	BS-7157R	1:200	ThermoFisher	Rockford	IL	USA
7	FTH-1	RB	AB75973	1:200	Abcam	Waltham	MA	USA
8	TfR1	MS	MABC1765	1:200	Sigma-Aldrich	St. Louis	MO	USA
9	Huc/D	MS	A21271	1:200	Invitrogen	Carlsbad	CA	USA
10	Huc/D	RB	AB184267	1:250	Abcam	Waltham	MA	USA
11	ALOX15	RB	AB244205	1:200	Abcam	Waltham	MA	USA
12	GPX4	MS	67763-1-1G	1:200	Proteintech	Rosemont	IL	USA
13	t-NFE2L2	RB	PA5-27882	1:200	ThermoFisher	Rockford	IL	USA
14	nNOS	RB	AB76067	1:200	Abcam	Waltham	MA	USA
15	β -actin	MS	3700S	1:5000	Cell Signaling	Danvers	MA	USA
16	GFAP	CK	AB4674	1:250	Abcam	Waltham	MA	USA
17	Tyrosine Hydroxylase	GT	ab317795	1:200	Abcam	Waltham	MA	USA
18	DRP1	RB	ab184247	1:200	Abcam	Waltham	MA	USA
19	ChAT	MS	CL3173	1:200	ThermoFisher	Rockford	IL	USA
20	ChAT	GT	AB144P	1:200	Sigma-Aldrich	St. Louis	MO	USA
21	nNOS	GT	GTX89962	1:200	GeneTex	Irvine	CA	USA

Supplementary Table 2: List of Secondary antibodies

No	Antibody	Host	Cat no.	Conc	Company	Fluor - ophore	City	State	Country
1	anti-mouse, Alexa 568	DK	A11041	1:400	Fisher Scientific	568	Waltham	MA	USA
2	anti-rabbit, Alexa 568	DK	A10042	1:400	Fisher Scientific	568	Waltham	MA	USA
3	anti-chicken, Alexa 488	GT	A11039	1:400	Fisher Scientific	488	Waltham	MA	USA
4	anti-rabbit, Alexa 488	DK	A21206	1:400	Fisher Scientific	488	Waltham	MA	USA
5	anti-mouse, Alexa 488	DK	A21202	1:400	Fisher Scientific	488	Waltham	MA	USA
6	anti-rabbit, Alexa 568	GT	A-11011	1:200	ThermoFisher	568	Rockford	IL	USA
7	anti-chicken, Alexa 633	GT	A-21103	1:200	ThermoFisher	633	Rockford	IL	USA
8	Anti-Chicken IgY H&L (Alexa Fluor® 488)	GT	ab150169	1:200	Abcam	488	Waltham	MA	USA
9	anti-Goat - 405	Dk	A48259	1:200	ThermoFisher	405	Rockford	IL	USA
10	anti-Mouse-405	DK	A48257	1:200	ThermoFisher	405	Rockford	IL	USA
11	anti-Rabbit-680	DK	A32802	1:200	ThermoFisher	680	Rockford	IL	USA
12	anti-Goat - 546	DK	A-11056	1:200	ThermoFisher	546	Rockford	IL	USA
13	anti-Mouse-680	DK	A10038	1:200	ThermoFisher	680	Rockford	IL	USA

Supplementary Table 3: List of TaqMan probes

No	Gene	TaqMan Probe ID	Gene Name
1	nNOS	Mm01208059_m1	Nitric oxide synthase 1 (neuronal)
2	TUBB3	Mm00727586_s1	Tubulin beta 3 class III
3	Nfe2l2	Mm00477784_m1	Nuclear factor, erythroid 2-like 2 (Nrf2)
4	18s rRNA	Mm03928990_g1	18S ribosomal RNA
5	HPRT1	Mm00446968_m1	Hypoxanthine guanine phosphoribosyl transferase 1
6	IL-6	Mm00446190_m1	Interleukin 6
7	TfR1	Mm00441941_m1	Transferrin receptor 1
8	GPX4	Mm00515041_m1	Glutathione peroxidase 4
9	DMT1	Mm00435363_m1	Solute carrier family 11 member 2 (DMT1)
10	SLC40A1	Mm01254822_m1	Solute carrier family 40 member 1 (ferroportin)

Supplementary Table 4: Human subject information related to the surgical procedure, gut specimens, medications, incidents of diabetes, clinical lab analysis for DM, iron metabolism, lipid profile and CRP.

Patient ID	GI region	Type of surgery	Sex	BMI kg/m^2	Age	Race	Hgb A1C (date)	Ferritin (date)	Transferrin (date)	CRP (date)	Cholesterol (date)	HDL	LDL	VLDL	Triglycerids	Non-HDL cholesteril	DM	Medications
EN-F01	Ascending colon	Colectomy partial laparoscopic right	M	21.25	71	White	-	-	453.3 mg/d	-	170 mg/dl	34	123	-	114	130	no	MOM[4] 30 ml daily for constipation
EN-F02	Ascending colon	Colectomy partial laparoscopic right	M	21.4	59	White	6.2%	-	-	-	57 mg/dll	20	28	-	47	37	no	NA[5]
EN-F03	Ascending colon	Colectomy partial laparoscopic right	F	46.76	59	White	5.30%	-	-	73.69 mg/L	161 mg/dl	43	91	27	137	-	no	NA
EN-F04	Ascending colon	Colectomy partial laparoscopic right	F	29.33	58	White	4.80%	-	-	-	199 mg/dl	110	79	-	50	89	no	NA
EN-F05	Sigmoid colon	Colectomy partial open left	F	34.94	68	White	5.40%	10.2 ng/ml	360 mg/dl	3.66 mg/L	96 mg/dl	23	-	-	128	132	IFG[1]	metamucil for constipation
EN-F06	Sigmoid colon	Colectomy partial laparoscopic left	F	26.1	54	White	4.80%	-	-	241 mg/dl	219 mg/d	64	142	13	65	-	no	NA
EN-F07	Sigmoid colon	Colectomy partial laparoscopic left	M	30.48	44	White	4.90%	-	-	-	275 mg/dl	42	216	17	86	-	no	NA
EN-F08	Sigmoid colon	Colectomy partial robotic XI left	F	37.69	61	White	-	-	-	-	-	-	-	-	-	-	DM[3] type 2/ use of insulin	Miralax
EN-F09	Ascending colon	Colectomy partial robotic XI right	M	41.05	54	Black	5.50%	-	-	-	117 mg/d	48	57	12	62	-	IFG[1]	Miralax
EN-F10	Ascending colon	Colectomy partial laparoscopic right	M	28.43	78	White	-	-	-	-	132 mg/dl	40	68	24	120	92	no	on chemo with leucovorin, FOLFOX[2]
EN-F11	Sigmoid colon	Colectomy partial laparoscopic left	M	32.22	73	White	-	237.5 ng/ml	180 mg/dl	-	159 mg/dl	35	101	23.4	117	124	no	NA
EN-F12	Ascending colon	Colectomy partial robotic XI right	M	22.23	67	White	5.30%	30 ng/L	-	5.7 mg/L	241 mg/dl	43	165	33	164	_	no	NA
EN-F13	Sigmoid colon	Colectomy partial robotic XI left	F	35.2	67	White	-	-	-	-	119 mg/dl	38	58	-	114	-	no	NA
EN-F14	Sigmoid colon	Colectomy partial robotic XI left	M	35.27	75	White	7.20%	-	-	-	106 mg/dl	41	50	-	-	-	DM[3] type 2	NA

IFG[1] - impaired fasting glucose; FOLFOX[2] - fluorouracil and oxaliplatin; DM[3] mellitus; MOM[4] - magnesium hydroxide; NA[5] - not applicable.

Supplementary Figure 1

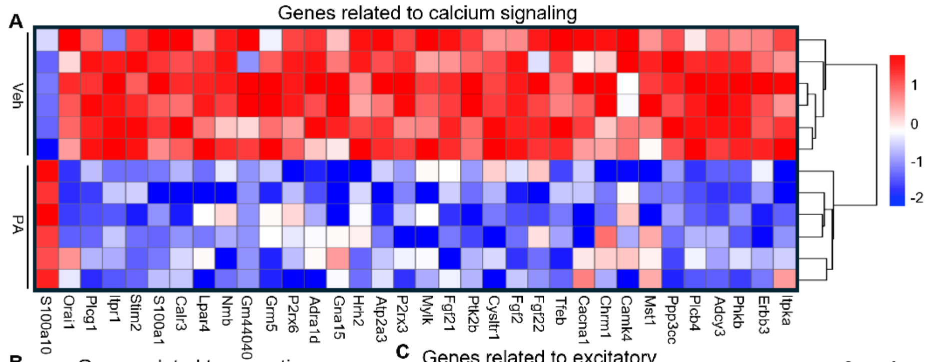

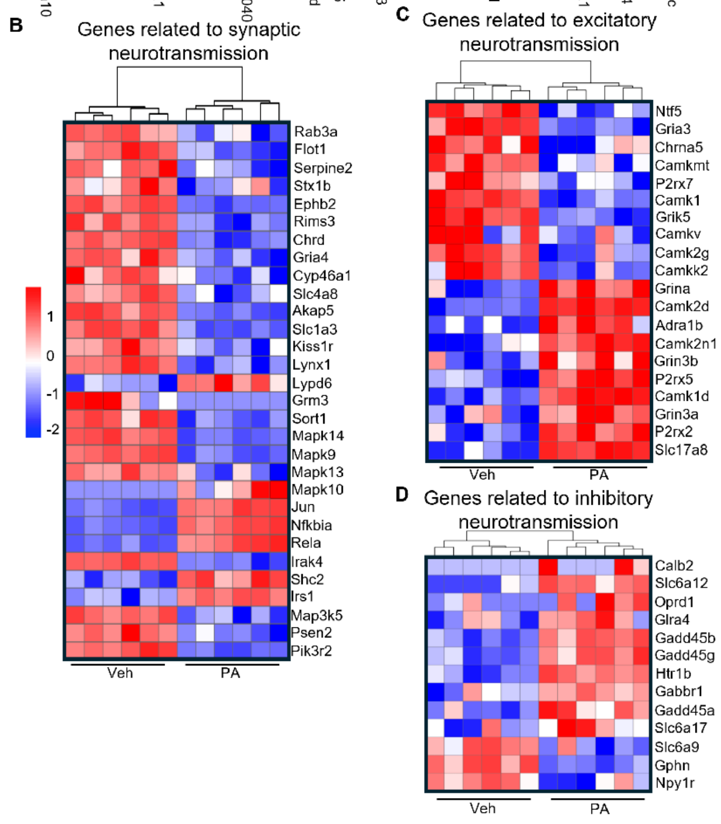

Supplementary Figure 1. Palmitic acid modulates genes involved in calcium signaling and neurotransmission in enteric neurons. (A-D) Heatmaps showing transcriptional changes in immortalized enteric neuronal cells (IM-FEN) treated with vehicle (Veh) or palmitic acid (PA, 0.5 mM) for 24 hours (n = 6 per group). Bulk RNA-seq was performed to assess global gene expression changes. (A) Calcium signaling-related genes including ion channels, calcium pumps, and calcium-binding proteins were significantly altered by PA, indicating disrupted intracellular calcium homeostasis. (B) Synaptic transmission-related genes including components of synaptic vesicles and exocytosis machinery were broadly downregulated following PA exposure, suggesting impaired synaptic function. (C) Genes involved in excitatory neurotransmission were reduced in PA-treated cells, consistent with suppression of excitatory signaling pathways. (D) Genes associated with inhibitory neurotransmission showed partial decreased expression, suggesting a broader disruption in the excitatory/inhibitory neuronal balance. Heatmap values represent log_2-transformed, z-score normalized gene expression. Columns represent individual biological replicates, clustered to illustrate treatment-specific transcriptional signatures.

Supplementary Figure 2

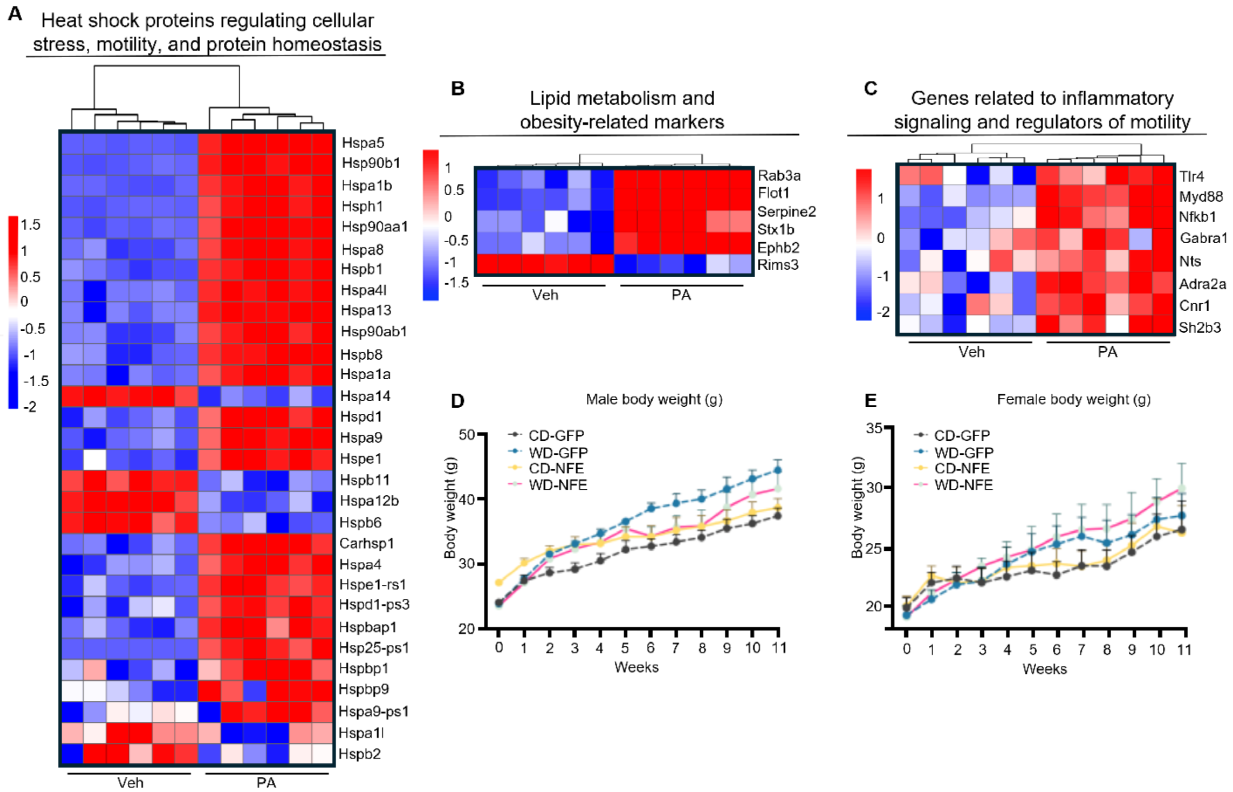

Supplementary Figure 2. Palmitic acid induces heat shock protein expression in enteric neurons and western diet increases body weight in mice. (A) Heatmap showing differential expression of heat shock proteins (HSPs) in IM-FEN cells treated with vehicle (Veh) or palmitate (PA, 0.5 mM) for 24 hours (n = 6 per group). Genes include molecular chaperones and protein- folding regulators involved in cellular stress responses. Values are $\log_2$-transformed, z-score normalized counts. Hierarchical clustering reveals distinct separation between Veh- and PA- treated cells, with robust upregulation of HSPs following PA exposure. (B) Heatmap of lipid metabolism and obesity-related gene expression in IM-FEN cells treated with vehicle (Veh) or palmitic acid (PA, 0.5 mM) for 24 h. (C) Heatmap of inflammatory signaling and neurotransmission-associated motility regulators in the same conditions. (D-E) Body weight measurements of male (D) and female (E) mice over a 12-week period. Mice were fed control diet (CD) or Western diet (WD) and received AAV-eGFP or AAV-Nfe2l2 at week 2. WD-fed animals of both sexes gained significantly more weight over time compared to RD-fed controls, irrespective of AAV treatment. Data are presented as mean ± SEM; n = 4-6 mice per group.

Supplementary Figure 3

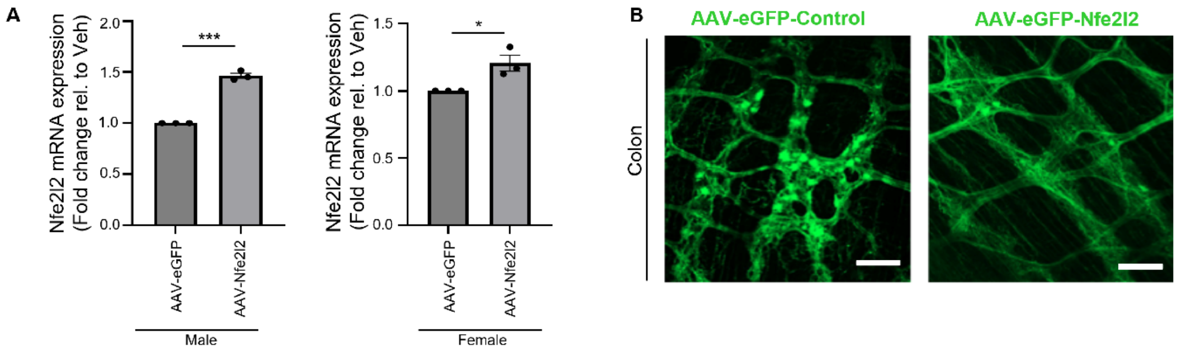

Supplementary Figure 3. AAV-mediated overexpression of Nfe2l2 enhances neuronal Nfe2l2 expression in the colon. A) Quantitative RT-PCR analysis showing increased colonic Nfe2l2 mRNA expression in AAV-Nfe2l2-treated mice compared to AAV-eGFP controls. Data represent technical triplicates from male and female samples and confirm successful AAV overexpression. Data represent mean ± SEM. Statistical analysis was performed using t-test. *P < 0.05; ***P < 0.001; B) Representative whole-mount confocal images of the myenteric plexus displaying eGFP fluorescence in mice treated with AAV-eGFP (Control) or AAV-Nfe2l2. Images confirm effective viral transduction across the enteric neuronal network. Scale bar, 50 µm.

Supplementary Figure 4

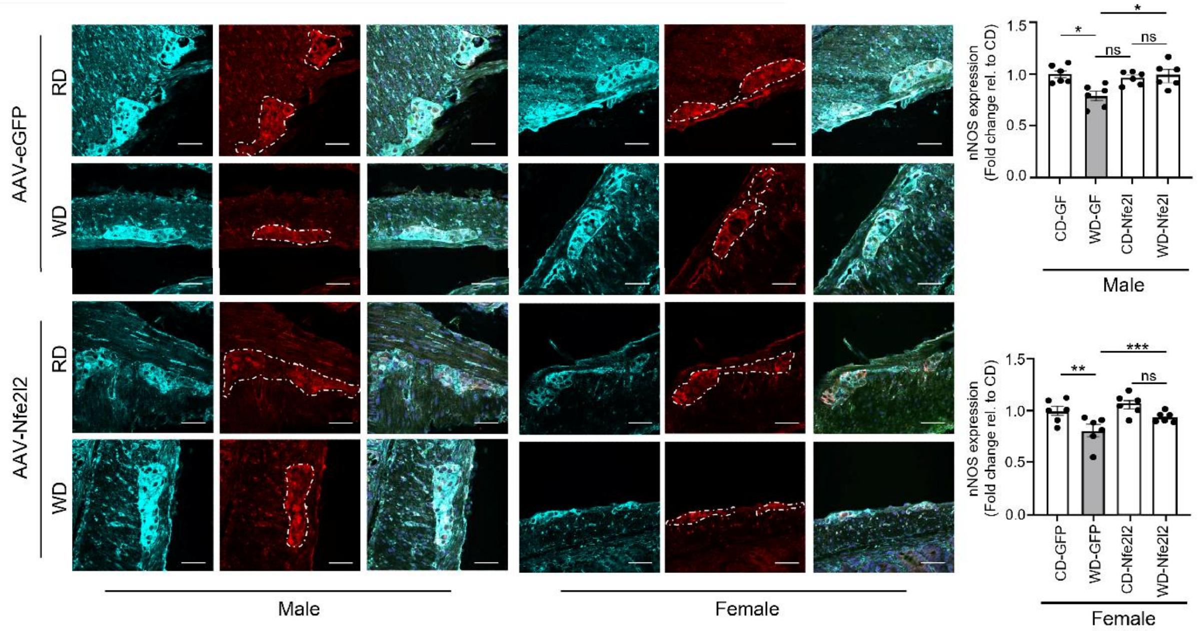

Supplementary Figure 4. AAV-Nfe2l2 preserves neuronal nitric oxide synthase (nNOS) expression in the colonic myenteric plexus of Western diet-fed mice. Representative immunofluorescence images of colon tissue sections from male and female mice fed control diet (CD) or Western diet (WD) for 12 weeks and treated with AAV-eGFP or AAV-Nfe2l2. Sections were co-stained for the pan-neuronal marker TUBB3 (cyan) and neuronal nitric oxide synthase (nNOS, red). Merged images highlight co-localization of nNOS within TUBB3+ neurons. AAV-Nfe2l2 administration preserved nNOS expression in WD-fed mice compared to AAV-eGFP controls. Quantification of the proportion of nNOS+ neurons among total TUBB3+ neurons across groups. A total of 28 mice were used: n = 4 mice per group for CD and WD AAV-eGFP, and n =3 mice per group for CD and WD AAV-Nfe2l2. From each mouse, 6-10 randomly selected myenteric ganglia were imaged and analyzed. Data represent mean ± SEM. Statistical analysis was performed using two-way ANOVA. *P < 0.05; **P < 0.01; ***P < 0.001; ns, not significant. Scale bars, 50 μm.

Supplementary Figure 5

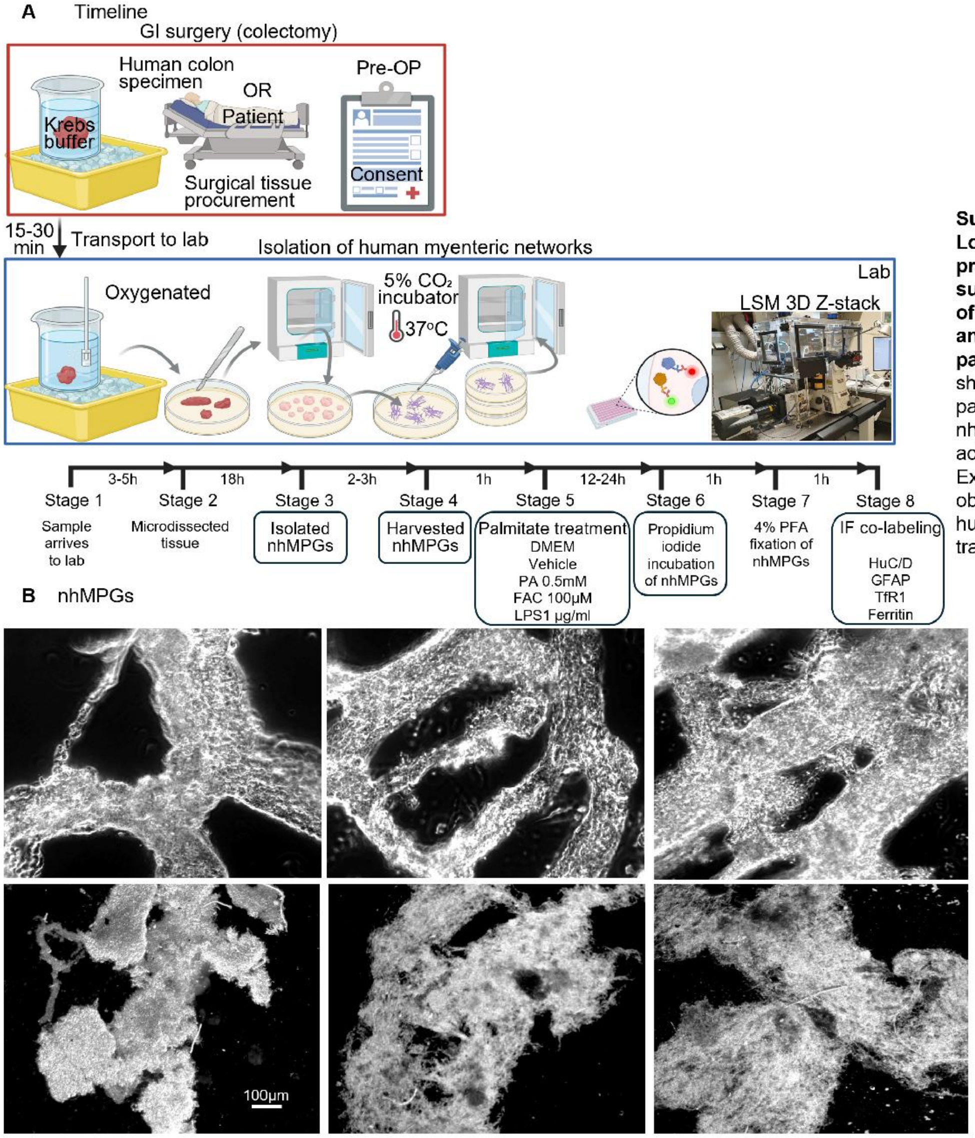

Supplementary Figure 5. Logistics and timeline for procurement of human colon surgical specimens, isolation of human networks of ganglia and in vitro experiments with palmitic acid. (A) Schematic showing the timeline from patient consent to isolation of nhMPG networks and palmitic acid induction experiments. (B) Examples of nhMPG networks obtained after colectomy in human patients are shown with transmitted light imaging.

Supplementary Figure 6

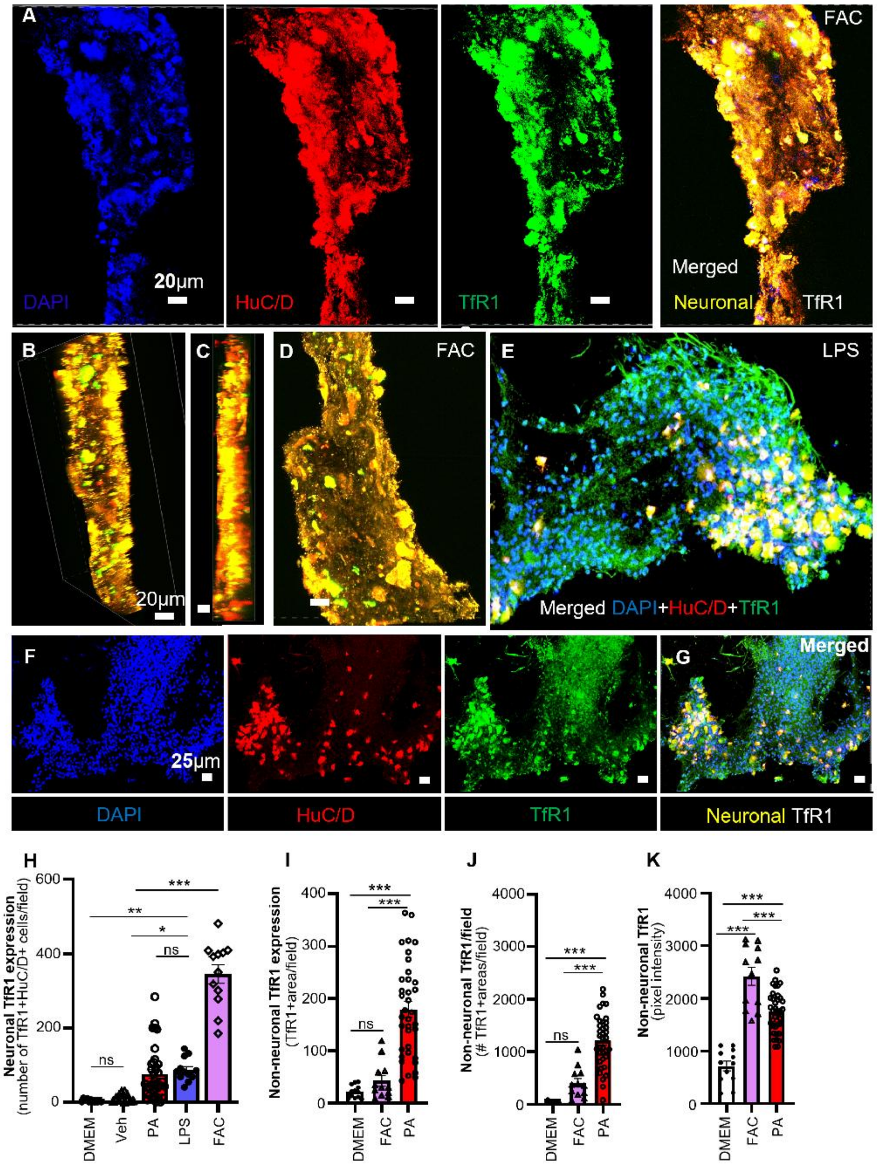

Supplementary Figure 6. Differential induction of neuronal and non-neuronal TfR1 expression by PA, FAC, and LPS in human nhMPG networks. (A-E) Representative confocal images of human networks of myenteric ganglia (nhMPG) treated with ferric ammonium citrate (FAC, 100 µM) or lipopolysaccharide (LPS, 1 µg/mL) for 24 h. Sections were stained for DAPI (blue), the pan-neuronal marker HuC/D (red), and transferrin receptor 1 (TfR1, green). Merged images show co-localization of neuronal TfR1 (yellow). Representative confocal images of FAC-treatment, shown as z-stack projections from different fields of view, increased neuronal TfR1 expression (A-D), while LPS also induced TfR1 expression in enteric neurons (E). (F-G) Additional examples of LPS-treated ganglia showing broad upregulation of TfR1 in both neuronal and non-neuronal regions. (H-K) Quantification of TfR1 expression across conditions. (H) Neuronal TfR1 expression/field was significantly increased by PA (0.5 mM) and LPS (1 µg/mL) to a similar extent, while FAC induced a markedly greater increase. (I) FAC induced neuronal TfR1 expression in a significantly larger number of HuC/D^{+} neurons per field compared to PA. (J) In contrast, non-neuronal TfR1 expression was significantly greater with PA than with FAC, based on both total area and number of distinct TfR1-positive regions per field. (K) Pixel intensity analysis revealed that FAC resulted in marginally but significantly higher TfR1 intensity than PA. All data were analyzed by one-way ANOVA followed by Tukey's post hoc test. Values represent mean ± SEM. *P < 0.05, **P < 0.01, ***P < 0.001. Quantification of TfR1 expression was performed using Nikon NIS-Elements co-localization module from 18 µm-thick z-stacks acquired at 0.5 µm intervals.

Supplementary Figure 7

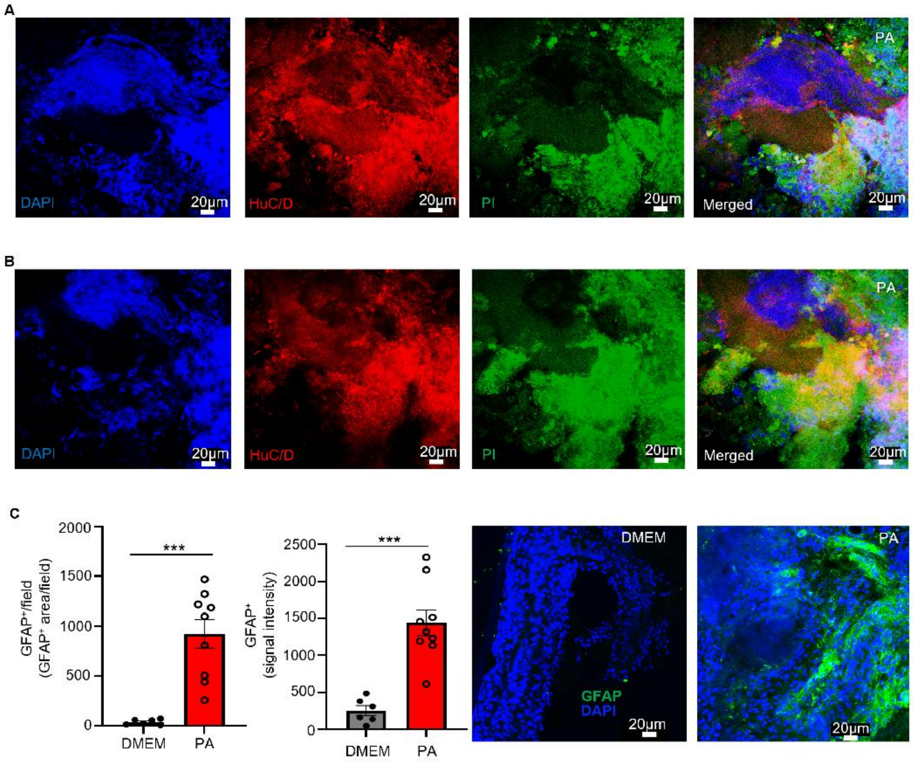

Supplementary Figure 7. Palmitic acid disrupts ganglionic morphology and induces glial activation in human nhMPG networks. (A-B) Representative confocal images showing structural abnormalities in human myenteric ganglia (nhMPG) following PA (0.5 mM, 24 h) treatment. Distortion or fragmentation of the ganglionic network was observed in a subset of patient samples. (C) PA treatment induced glial fibrillary acidic protein (GFAP) expression in HuC/D glial cells within the nhMPG, consistent with reactive gliosis. GFAP is not typically detected in healthy human enteric glia. Quantification was performed in ganglionic networks from a representative patient (n = 9 networks per condition). Values are expressed as mean ± SEM. $*P < 0.05$, $**P < 0.01$, $***P < 0.001$.

Supplementary Figure 8

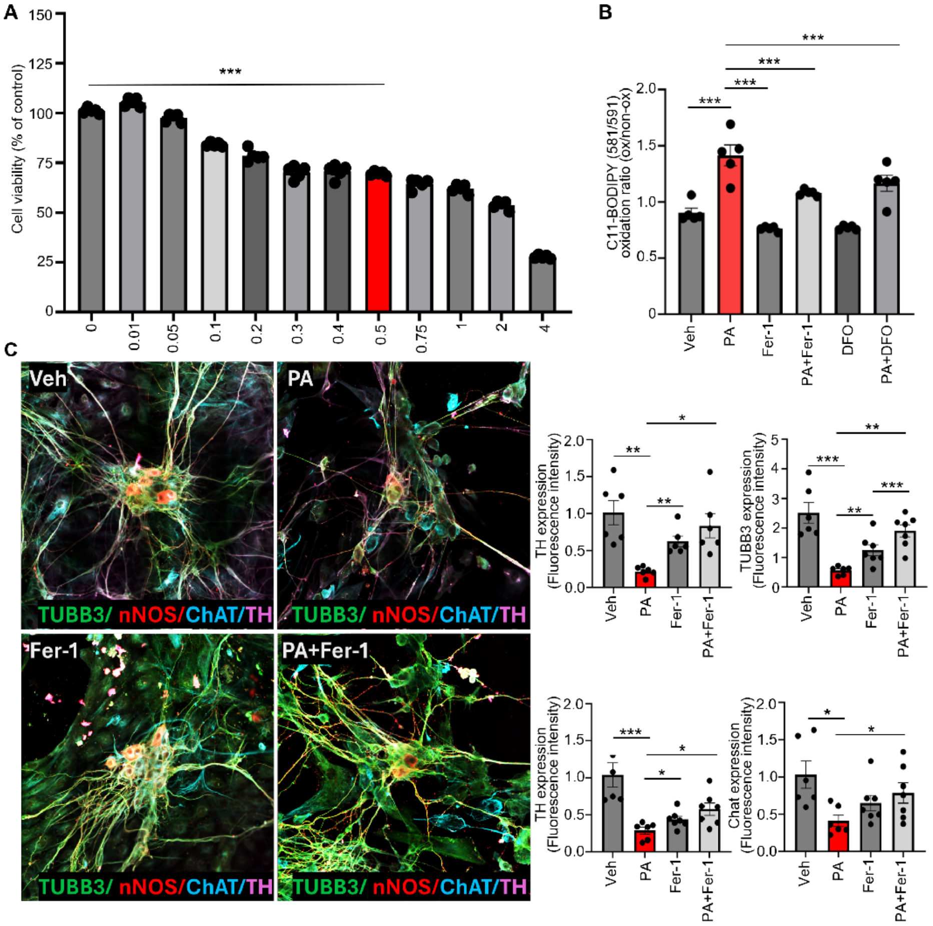

Supplementary Figure 8. Palmitate induces lipid peroxidation and ferroptosis-dependent loss of nNOS, ChAT, and TH enteric neurons. (A) Cell viability of IM-FEN enteric neurons treated for 24 h with increasing concentrations of palmitate (PA, 0.01-4 mM) measured by CellTiter-Glo. 0.5 mM PA (red bar) was chosen for subsequent experiments as it reduced viability without causing overt cell loss. (B) C11-BODIPY fluorescence in IM-FEN cells treated for 24 h with vehicle (Veh), PA (0.5 mM), ferrostatin-1 (Fer-1, 10 μM), PA + Fer-1, deferoxamine (DFO, 50 μM), or PA + DFO. PA increases lipid peroxidation (oxidized C11-BODIPY signal), which is attenuated by Fer-1 and DFO. Histogram shows normalized oxidized/reduced C11-BODIPY fluorescence relative to Veh. (C) Confocal images of primary enteric neuron networks treated with Veh, PA, Fer-1, or PA + Fer-1 for 24 h and stained for TUBB3 (green), nNOS (red), ChAT (cyan), and TH (magenta). Quantification of nNOS-, ChAT-, and TH-expressing neurons demonstrates loss of all three neuronal populations with PA, partially restored by Fer-1. Scale bars: 50 μm. Data represent mean ± SEM from three independent experiments; Values are expressed as mean ± SEM. *P < 0.05, **P < 0.01, ***P < 0.001.

Supplementary Figure 9

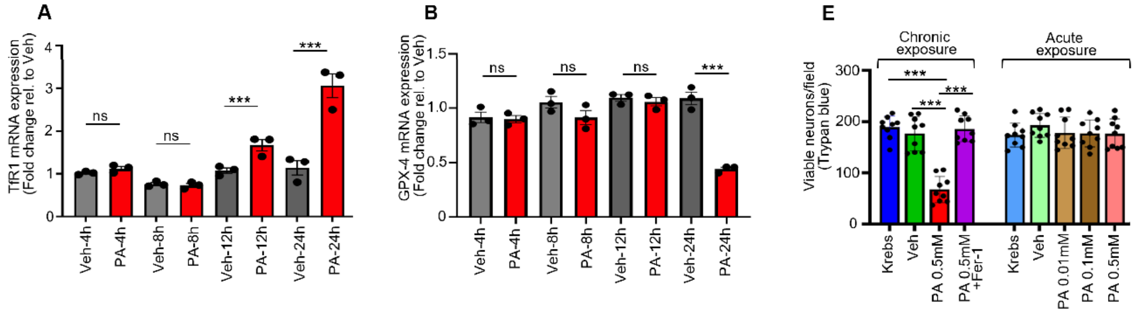

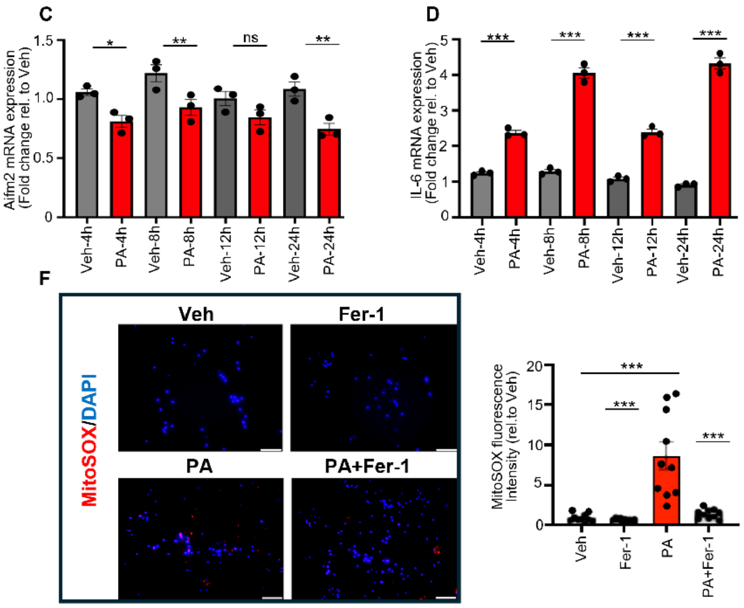

Supplementary Figure 9. Time dependent induction of ferroptosis genes, loss of viability, and mitochondrial ROS during palmitate exposure in enteric neurons. (A-D) qRT-PCR analysis of ferroptosis-related genes transferrin receptor 1 (Tfr1), Il6, Gpx4, and Aifm2 in IM-FEN enteric neurons treated with vehicle (Veh) or palmitate (PA, 0.5 mM) for 0, 4, 12, and 24 h. Data are expressed as fold change in mRNA relative to time-matched Veh. (E) Trypan Blue cell viability assay of IM-FEN enteric neurons showing that acute PA exposure does not significantly reduce viability, whereas chronic PA markedly decreases viable neurons, an effect prevented by chronic Fer-1 co-treatment. (F) Representative images of MitoSOX Red (mitochondrial superoxide) and DAPI in IM-FEN neurons treated with Veh or PA for 24 h, with quantification of MitoSOX fluorescence intensity normalized to Veh, demonstrating increased mitochondrial ROS after PA exposure. Scale bars: 50 µm. Data represent mean ± SEM from three independent experiments; Values are expressed as mean ± SEM. *P < 0.05, **P < 0.01, ***P < 0.001; ns, not significant.

Supplementary Figure 10

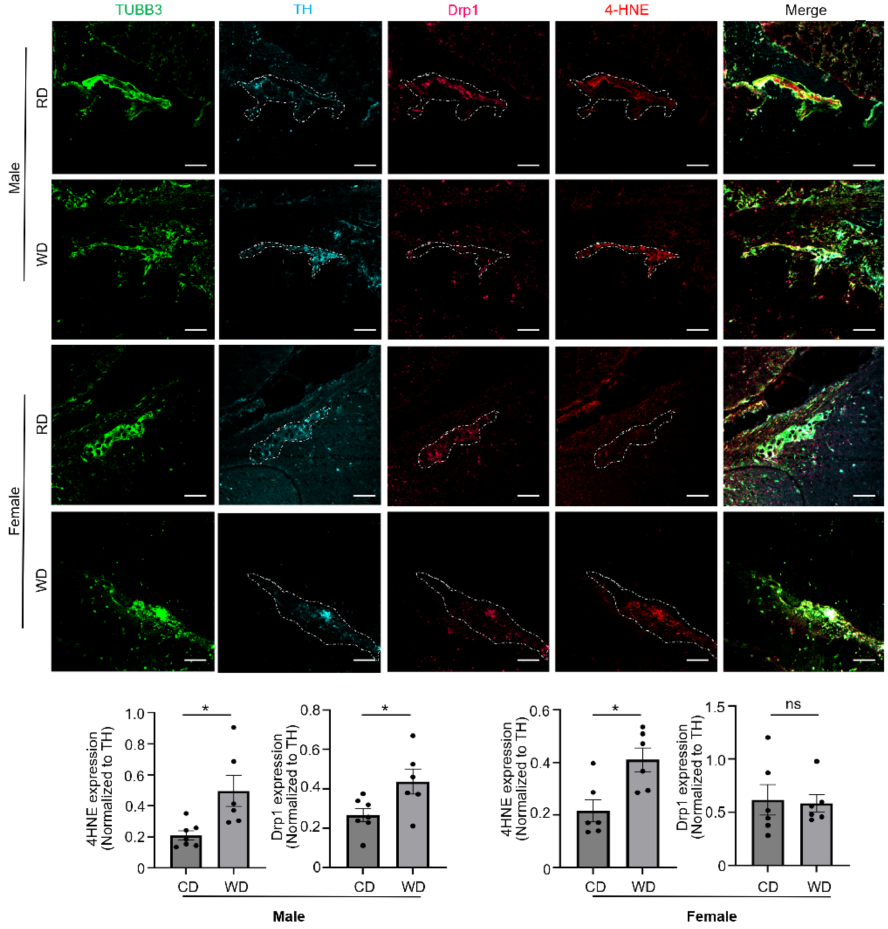

Supplementary Figure 10. Western diet increases Drp1 and 4-HNE in TH-positive myenteric neurons *in vivo*. Representative confocal images of distal colon myenteric plexus from mice fed control diet (CD) or Western diet (WD) for 12 weeks, stained for the pan-neuronal marker TUBB3 (green), tyrosine hydroxylase (TH, cyan), the mitochondrial fission protein Drp1 (magenta), and the lipid peroxidation marker 4-HNE (red). Dashed lines outline TH-positive neuronal cell bodies and processes. Merged images show increased Drp1 and 4-HNE within TH-positive neurons in Western diet exposed mice compared with controls, mapping mitochondrial stress and lipid peroxidation in this catecholaminergic subset. Bar graphs quantify TH-positive neuron density and Drp1 and 4-HNE fluorescence intensity within TH-positive neurons. Scale bars: 20 µm. Data represent mean ± SEM from n = 4 mice per group; Data represent mean ± SEM from three independent experiments; Values are expressed as mean ± SEM. *P < 0.05, **P < 0.01, ***P < 0.001; ns, not significant.

Supplementary Figure 11

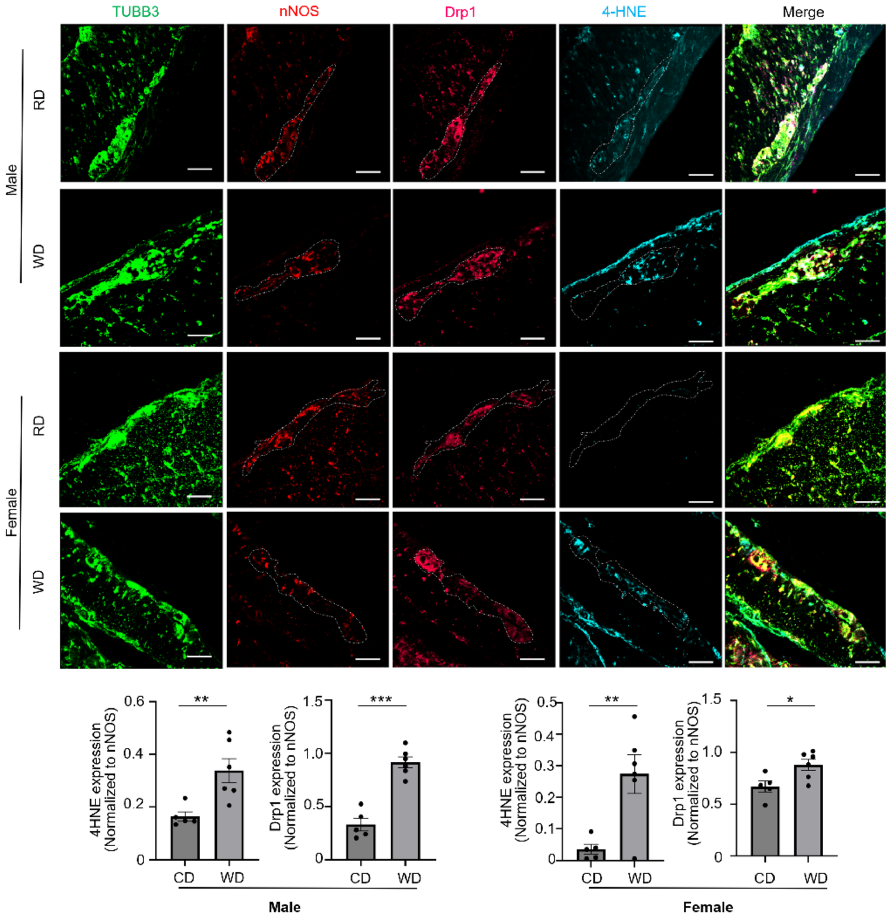

Supplementary Figure 11. Western diet increases Drp1 and 4-HNE in nNOS-positive myenteric neurons in vivo. Representative confocal images of distal colon myenteric plexus from mice fed control diet (CD) or Western diet (WD) for 12 weeks, stained for the pan-neuronal marker TUBB3 (green), neuronal nitric oxide synthase (nNOS, red), the mitochondrial fission protein Drp1 (magenta), and the lipid peroxidation marker 4-HNE (cyan). Dashed lines outline nNOS-positive neuronal cell bodies and processes. Merged images show increased Drp1 and 4-HNE signal within nNOS-positive neurons in Western diet exposed mice compared with controls, mapping mitochondrial stress and lipid peroxidation in this nitrergic subset. Bar graphs quantify nNOS-positive neuron density and Drp1 and 4-HNE fluorescence intensity within nNOS-positive neurons. Scale bars: 20 µm. Data represent mean ± SEM from n = 4 mice per group; Data represent mean ± SEM from three independent experiments; Values are expressed as mean ± SEM. *P < 0.05, **P < 0.01, ***P < 0.001.

Supplementary Figure 12

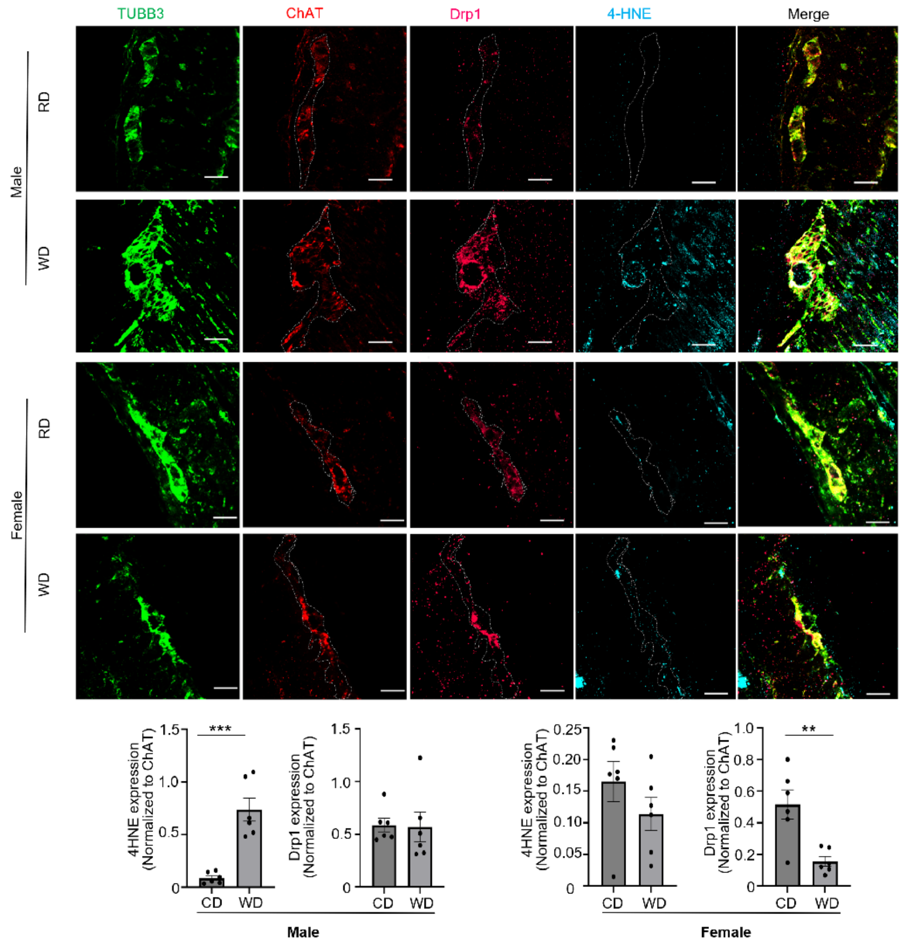

Supplementary Figure 12. Western diet alters Drp1 and 4-HNE in ChAT-positive myenteric neurons in a sex-dependent manner. Representative confocal images of distal colon myenteric plexus from mice fed control diet (CD) or Western diet (WD) for 12 weeks, stained for the pan-neuronal marker TUBB3 (green), choline acetyltransferase (ChAT, red), the mitochondrial fission protein Drp1 (magenta), and the lipid peroxidation marker 4-HNE (cyan). Dashed lines outline ChAT-positive neuronal cell bodies and processes. Merged images show increased 4-HNE signal within ChAT-positive neurons in Western diet exposed mice compared with controls, consistent with enhanced lipid peroxidation. Quantification demonstrates that Drp1 intensity within ChAT-positive neurons is unchanged in males and reduced in females, whereas ChAT-positive neuron density in females is not significantly altered. Scale bars: 20 µm. Data represent mean ± SEM from three independent experiments; Values are expressed as mean ± SEM. *P < 0.05, **P < 0.01, ***P < 0.001.

Supplementary Figure 13

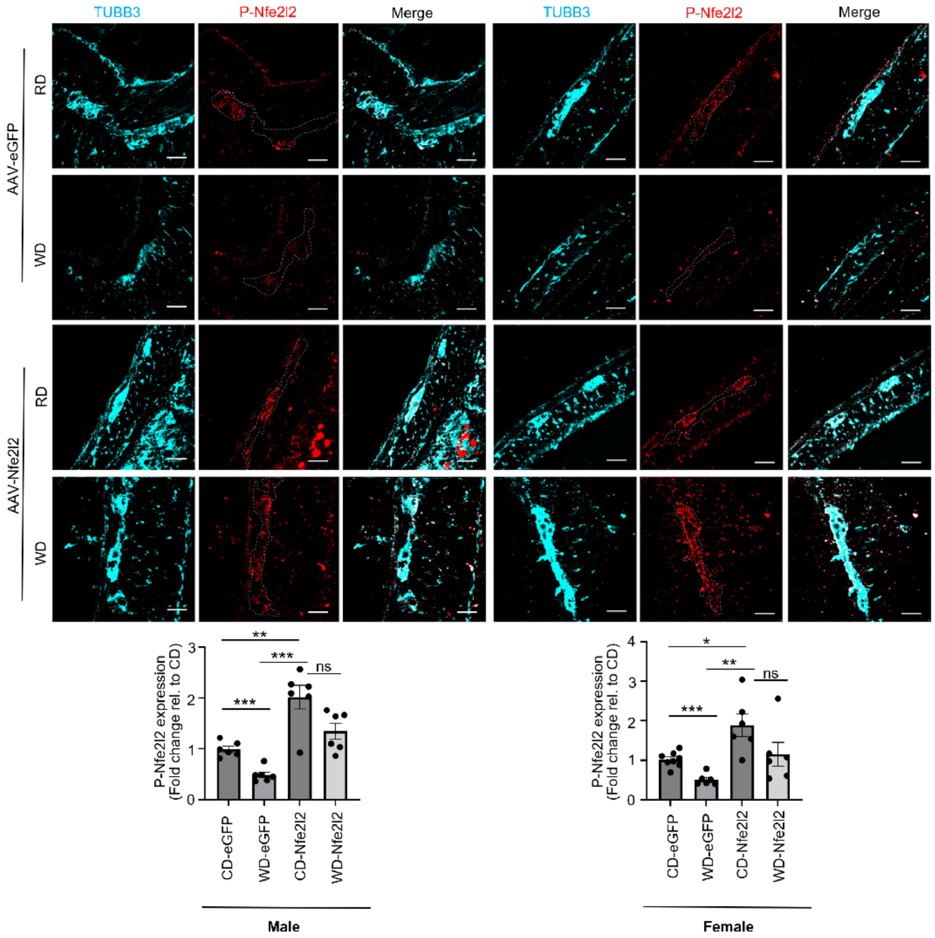

Supplementary Figure 13. Western diet suppresses phospho-Nfe2l2 in myenteric neurons and AAV Nfe2l2 restores antioxidant signaling. Immunofluorescence staining of distal colon sections from male and female mice fed control diet or Western diet and treated with AAV-eGFP or AAV-Nfe2l2, co-stained for TUBB3 (cyan) and phospho Nfe2l2 (P-Nfe2l2, red). Merged images show reduced neuronal P Nfe2l2 signal in Western diet AAV eGFP groups and enhanced P-Nfe2l2 expression within TUBB3$^+$ neurons in Western diet AAV Nfe2l2 treate mice. Histograms show fold change in TUBB3$^+$ neuron density and P-Nfe2l2 fluorescence intensity within TUBB3$^+$ neurons, normalized to the control diet AAV eGFP group. Scale bars: 50 μm. Data represent mean ± SEM from n = 3-4 mice per group. Statistical analysis was performed using two-way ANOVA. *P < 0.05; **P < 0.01; ***P < 0.001; ns, not significant.

Supplementary Figure 14

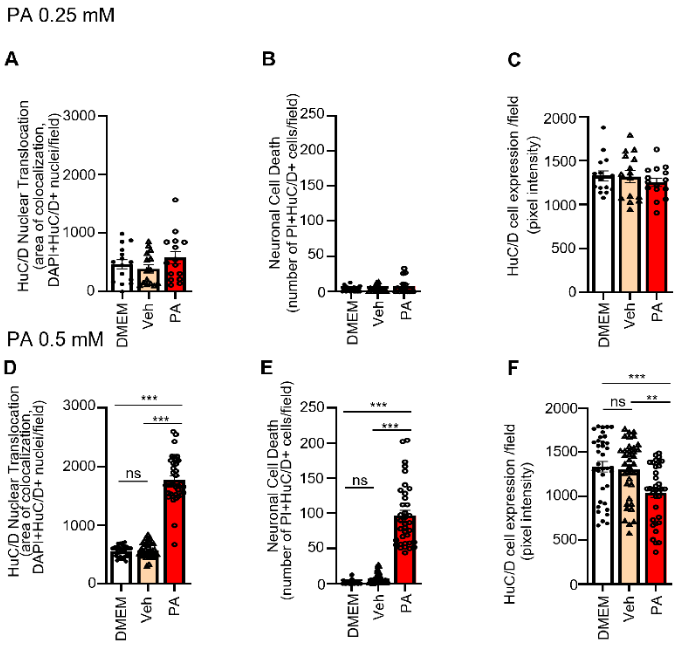

Supplementary Figure 14. Concentration-dependent effect of palmitic acid on induction of neuronal cell death in nhMPG networks. Networks of human myenteric ganglia (nhMPG) isolated from GI surgical specimens were exposed in vitro to vehicle (Veh, DMEM, 0.25mM or 0.5mM PA concentration for 24h). PA 0.25mM consistently mirrored vehicle responses and therefore did not trigger ferroptotic injury. PA had no effect on (A) HuC/D nuclear translocation (stress response in neurons), (B) neuronal cell death, PI nuclear staining) and (C) HuC/D cell expression. (D-F) In contrast, PA 0.5mM elicited robust cell-death and stress-response signals. Statistical comparisons were performed by one-way ANOVA followed by Tukey's post hoc test. Data are presented as mean ± SEM; $*P < 0.05$, $**P < 0.01$, $***P < 0.001$; ns, not significant. Quantification of cell death and HuC/D translocation was conducted using Nikon NIS-Elements colocalization tools in 18-μm confocal z-stacks acquired at 0.5-μm intervals. A total of 36 ganglionic networks per treatment group were analyzed. Human colonic tissue was obtained from 3 human subjects for each treatment, with 12 z-stacks from distinct nhMPG networks per human subject used for all colocalization and statistical analyses.

Supplementary Figure 15

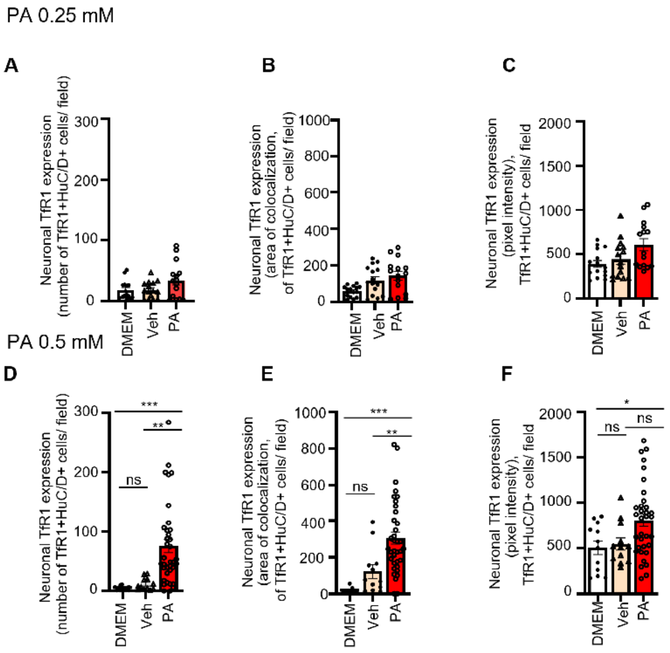

Supplementary Figure 15. Concentration-dependent increase in neuronal TfR1 activation by palmitic acid (PA) in nhMPG networks. The effect of palmitic acid was restricted to 0.5mM concentration. A lower concentration of 0.25mM had no effect on TfR1 activation. Networks of human myenteric ganglia (nhMPG) isolated from colectomy specimens were exposed ex vivo to Veh, DMEM, PA 0.25mM or PA 0.5mM. PA (0.25mM) did not increase TfR1 expression in (A) the number of HuC/D$^+$TfR1$^+$ neurons, (B) TfR1$^+$ area of colocalization with HuC/D$^+$, and (C) the pixel intensity of neuronal TfR1 immunoreactivity. In contrast, PA 0.5mM produced consistent evidence of ferroptotic signaling, with (D) significant increases in HuC/D$^+$TfR1$^+$ neuronal counts, (E) TfR1$^+$ area per neuron and (F) neuronal TfR1 fluorescence intensity. Statistical comparisons were performed using one-way ANOVA followed by Tukey's post hoc test. Data are reported as mean ± SEM; $^*P < 0.05$, $^{**}P < 0.01$, $^{***}P < 0.001$; ns, not significant. Quantification of TfR1 expression was performed using Nikon NIS-Elements colocalization tools in 18-μm confocal z-stacks collected at 0.5-μm optical intervals. A total of 36 ganglionic networks per treatment group were analyzed. Human colonic tissue was obtained from 3 GI surgical cases, with 12 z-stacks from distinct nhMPG networks per human subject included in colocalization and statistical analyses.

Supplementary Figure 16

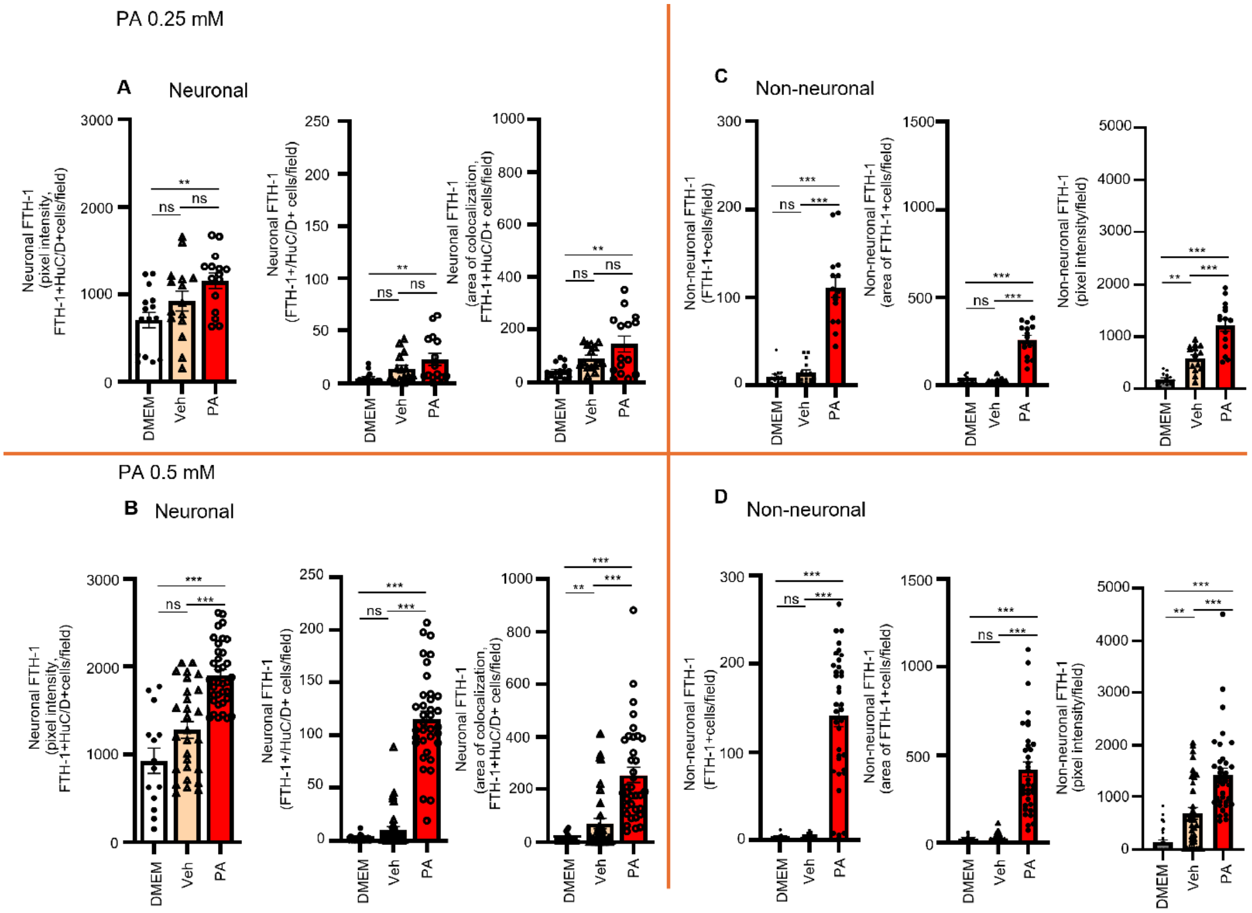

Supplementary Figure 16. Concentration-dependent Ferritin (FTH-1) ferroptotic responses to palmitic acid in human myenteric ganglia, with neuronal susceptibility occurring mainly at 0.5mM PA. Networks of human myenteric ganglia (nhMPG) isolated from colectomy specimens were treated in vitro with vehicle, DMEM, PA 0.25mM or PA 0.5mM concentration. Ferritin (FTH-1) activation in neuronal (HuC/D$^+$) and non-neuronal (HuC/D$^-$) cell populations was analyzed for complementary quantitative parameters. (A) PA 0.25mM did not increase neuronal Ferritin in nhMPG networks compared to Veh, whereas upregulation occurred in comparison to the DMEM group. (B) In contrast, PA 0.5mM triggered a ferroptotic response, with significant increases in Ferritin (FTH-1) pixel intensity in HuC/D$^+$ neurons, number of Ferritin$^+$HuC/D$^+$ neurons, and Ferritin-HuC/D colocalized area, all reflecting robust neuronal FTH-1 upregulation. (C, D) In non-neuronal cells, PA 0.25mM or PA 0.5mM both elicited ferroptotic changes in comparison to Veh across all metrics: number of Ferritin$^+$ non-neuronal cells, non-neuronal area, and pixel intensity in non-neuronal Ferritin$^+$cells. Statistical comparisons were performed by one-way ANOVA followed by Tukey's post hoc test. Values are presented as mean ± SEM; $^*P<0.05$, $^{**}P < 0.01$, $^{***}P < 0.001$; ns, not significant. Quantification of Ferritin expression and colocalization was performed using Nikon NIS-Elements on 18-μm confocal z-stacks acquired at 0.5-μm optical intervals. Thirty-six ganglionic networks per treatment group were analyzed. Colonic tissue was collected from 3 human donors, with 12 z-stacks from distinct nhMPG networks per human subject specimen included in all analyses.
