# Supplementary figures and images for: Western diet induces iron-dependent enteric neurodegeneration via ferroptosis
Source: J Clin Invest. 2026 Apr 21;136(11):e196113. doi: 10.1172/JCI196113 (PMC13221235; doi:10.1172/JCI196113)

Figure 1C

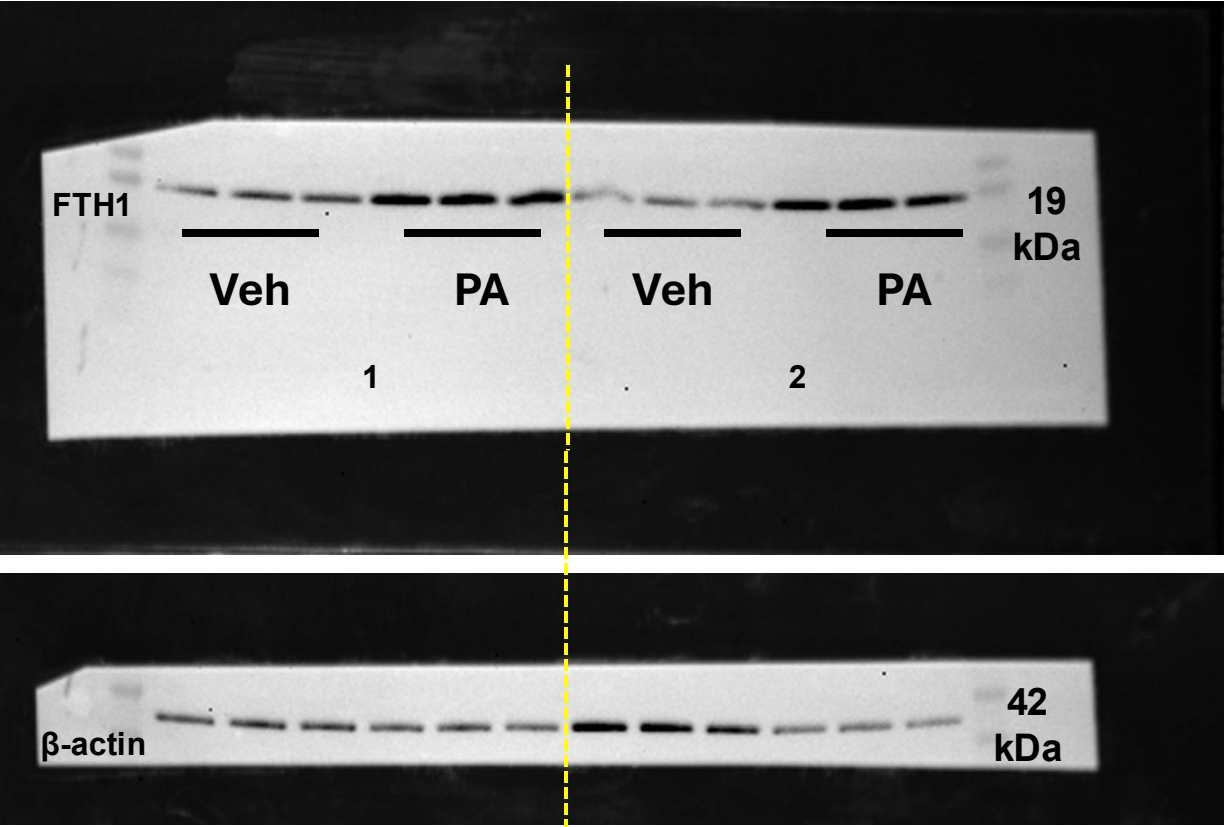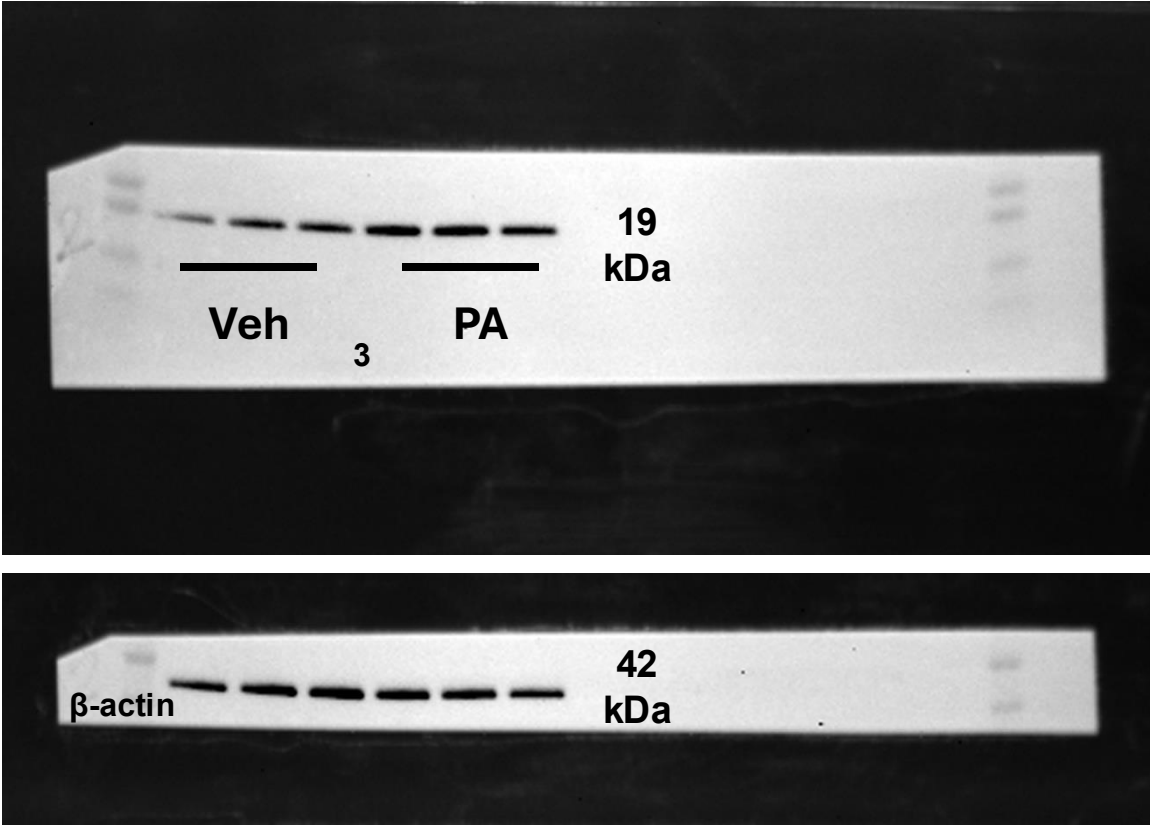

**Figure 1D**

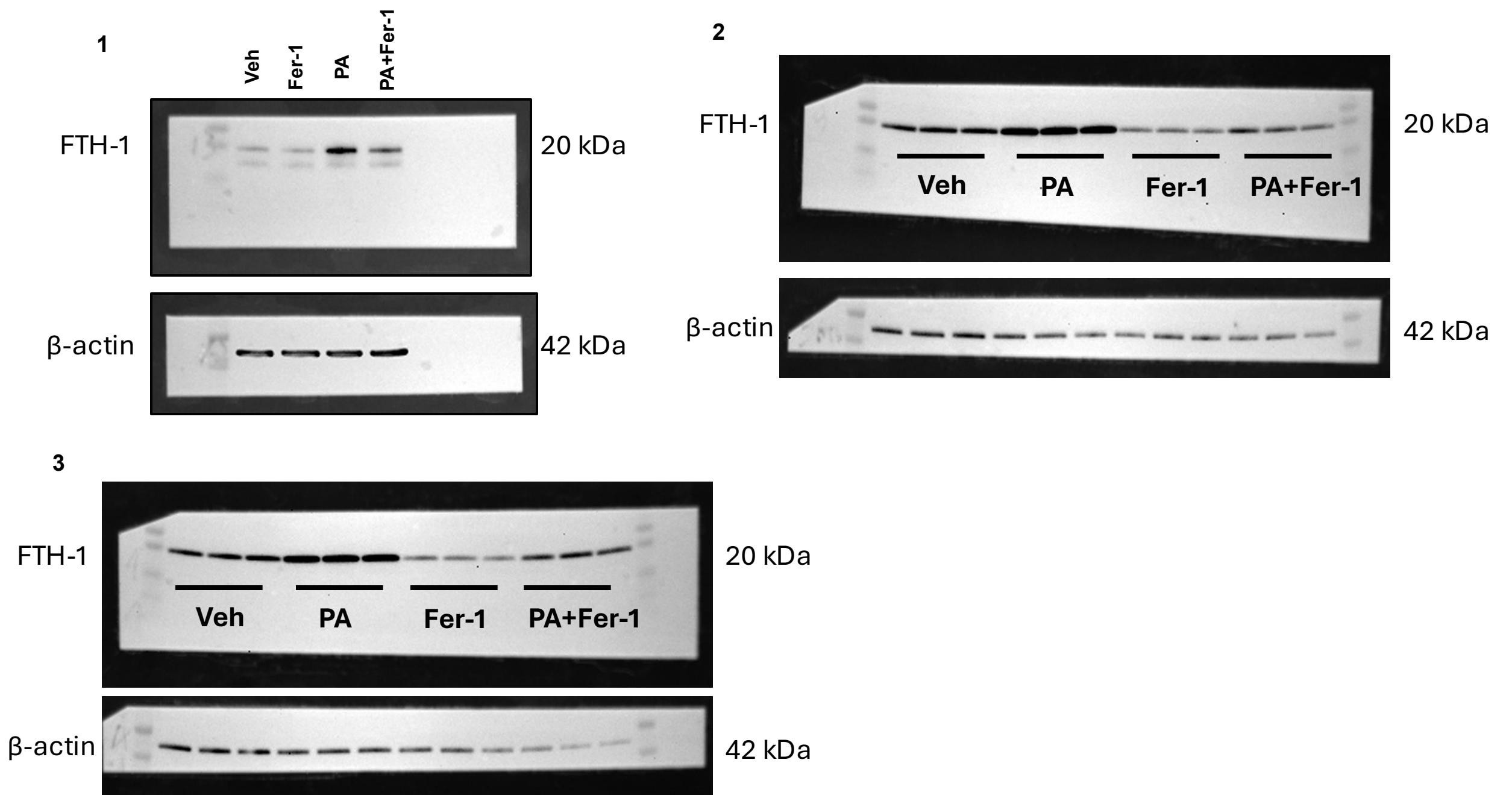

Supplement: Unedited blot and gel images [file jci-136-196113-s207.pdf]
